# Robust inhibitory glycinergic transmission and the effect of bafilomycin, folimycin and EIPA: lessons from the auditory brainstem

**DOI:** 10.3389/fncel.2025.1625868

**Published:** 2025-10-15

**Authors:** Erika Pizzi, Lina N. Hofmann, Abhyudai Singh, Eckhard Friauf

**Affiliations:** 1 Animal Physiology Group, Department of Biology, University of Kaiserslautern-Landau, Kaiserslautern, Germany; 2 Department of Electrical and Computer Engineering, Biomedical Engineering, Mathematical Sciences, Interdisciplinary Neuroscience Program, University of Delaware, Newark, DE, United States

**Keywords:** synaptic vesicle cycle, patch-clamp recordings, V-ATPase, Na^+^/H^+^ exchanger, inhibitory synapses

## Abstract

Sustained synaptic transmission requires the continuous replenishment of released synaptic vesicles (SVs). This process is particularly critical in neuronal circuits that operate at high rates and with high temporal precision, such as those in the auditory brainstem. Here, we investigated the effect of SV (re-)filling on inhibitory synapses between the medial nucleus of the trapezoid body (MNTB) and the lateral superior olive (LSO). These synapses transmit information with high speed and fidelity, properties essential for auditory computations such as sound localization. We specifically examined the role of the vacuolar ATPase (V-ATPase), a proton pump that acidifies the SV lumen to enable neurotransmitter loading. Using patch-clamp recordings in acute mouse slices, we assessed synaptic function under control conditions and during continuous V-ATPase inhibition with bafilomycin or folimycin. Contrary to our initial hypothesis, pharmacological inhibition caused only moderate impairment of sustained transmission. Even under high drug concentrations and intense stimulation (e.g., 100 Hz for 4 min), steady-state responses declined only to ~33% of control. Similar reductions were observed in the replenishment rate, the size of the readily releasable pool, and the cumulative eIPSC amplitude. Quantal size decreased gradually, reaching ~70% of control. Recovery from synaptic depression persisted in the presence of V-ATPase blockade, although it was less efficient. Together, these findings indicate that MNTB-LSO synapses are relatively resistant to V-ATPase inhibition, suggesting that SV replenishment does not rely solely on V-ATPase activity. Alternative acidification mechanisms may contribute, and among potential candidates, the Na^+^/H^+^ exchanger isoform NHE6 showed strong immunoreactivity in glycinergic MNTB axon terminals contacting LSO somata. This identifies NHE6 as a promising target for future investigation.

## Introduction

Synaptic transmission, the primary means of interneuronal information transfer, is a complex process involving several pre- and postsynaptic components. On the presynaptic side, dozens to thousands of spherical synaptic vesicles (SVs) are packed into axon terminals. Only a fraction of these is morphologically docked and functionally primed, thus forming the readily releasable pool (RRP). The remaining SVs form the reserve pool and the recycling pool (Rizzoli and Betz, 2005; Alabi and Tsien, 2012; Neher, 2015).

An action potential (AP) entering an axon terminal provides the depolarization necessary to open voltage-gated Ca^2+^ channels, triggering a rapid rise in local [Ca^2+^]_i_. As a result, a fraction of release-competent SVs fuse with the plasma membrane and release their transmitter cargo into the synaptic cleft via regulated rapid exocytosis (Katz, 1969; Südhof, 2013; Silva et al., 2021). The outer and inner diameters of most SVs are ~40 nm and ~25 nm, respectively (Schikorski and Stevens, 1997; Neef et al., 2007); for further references see (Clements, 1996). For the intravesicular volume, values of ~10^−5^ μm^3^ (10^−20^ L; Schikorski and Stevens, 1997), ~40 zL (4*10^−20^ L; Clarkson et al., 2016) and ~2*10^−20^ L (Takamori et al., 2006) have been reported. At excitatory axon terminals, the intravesicular glutamate concentrations are 60–100 mM (Burger et al., 1989; Bruns and Jahn, 1995), corresponding to 3,000–6,000 transmitter molecules per SV (Danbolt, 2001; Freche et al., 2011). Inhibitory synapses have been much less studied than their excitatory counterparts.

**Figure 1:**
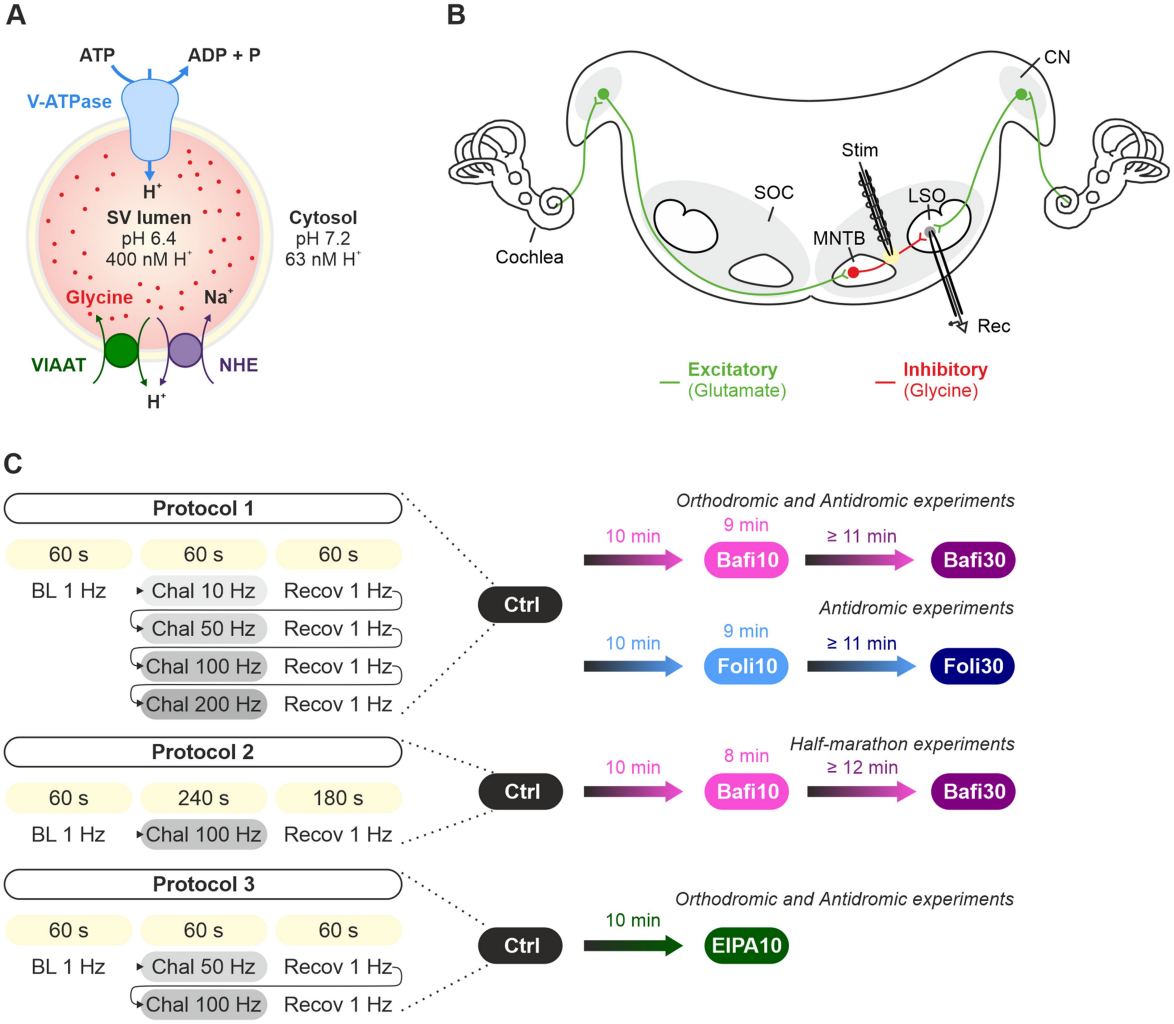
Analysis of synaptic transmission at glycinergic MNTB-LSO inputs under control conditions and upon pharmacological treatment with the V-ATPase blockers Bafi and Foli and the NHE blocker EIPA. **(A)** Schematic of the vacuolar transporters involved in proton pumping and thus in VIAAT-mediated filling of SVs with the inhibitory neurotransmitter glycine. **(B)** Schematic of the MNTB-LSO circuit in a mouse coronal brainstem slice. **(C)** Schematic of the three stimulation protocols used. All protocols started with a 1-Hz|60-s stimulation period to establish a baseline (BL, average peak amplitude = 100%). Protocol 1 included four challenge periods at 10, 50, 100 and 200 Hz, each followed by a recovery period at 1 Hz. Protocol 1 was used to assess the effect of Bafi (2 μM), Foli (1 μM) and EtOH (0.1%; sham control experiments, Supplementary Figures S1–S4). Protocol 2 (half-marathon experiments) consisted of a 100-Hz challenge period lasting 240 s which was followed by a 180-s recovery period; it was used to evaluate Bafi effects at a concentration of 5 μM. Protocol 3 consisted of two challenge periods (50 and 100 Hz) and two recovery periods; it was used to evaluate the effects of the NHE inhibitor EIPA (100 μM) on synaptic transmission and on AP fidelity in antidromic stimulation experiments. A 6-s long pause was introduced after the BL and each 60-s recovery.

Both the *de novo* filling of SVs and the reloading of recycled SVs with transmitter molecules are achieved by a cascade of active transport processes. The acidification of the SV lumen is generated by a multi-subunit, primary active vacuolar (H^+^)-ATPase (H^+^ pump) in the SV membrane (V-ATPase; Figure 1A). This V-ATPase couples the hydrolysis of ATP with the transport of H^+^ into the vesicle lumen. Results from the *Torpedo marmorata* electric organ and glutamatergic neurons show that V-ATPase activity generates a pH value in the SV lumen that is 1.4–1.7 units more acidic than the pH of ~7.2 in the cytosol (Harlos et al., 1984; Chaudhry et al., 2008; Ruffin et al., 2014). Scant evidence from GABAergic SVs suggests a less acidic luminal pH of ~6.4 (Egashira et al., 2016). The electrochemical H^+^ gradient generated by the V-ATPase is used by secondary-active neurotransmitter transporters to drive the transport of transmitter molecules from the cytosol into the SV lumen via an exchange mechanism (Edwards, 2007; Anne and Gasnier, 2014). While the cytosolic transmitter concentration is ~10 mM, the luminal concentration is ~10-fold higher.

In the case of GABA and glycine, the two major inhibitory transmitters in the CNS, the secondary-active transporter is VIAAT (vesicular inhibitory amino acid transporter, also known as vesicular GABA transporter VGAT) (McIntire et al., 1997; Sagné et al., 1997; Wojcik et al., 2006; Zafra and Giménez, 2008; Figure 1A). Glycine, the transmitter at fast inhibitory synapses, is primarily supplied to VIAAT by the glycine transporter GlyT2 via reuptake of released glycine from the extracellular space (Aubrey et al., 2007; Rousseau et al., 2008; Apostolides and Trussell, 2013; Brill et al., 2021). Endogenous glycine concentrations in presynaptic boutons were reported at 20–40 mM or at ~5 mM (Supplisson and Roux, 2002; Apostolides and Trussell, 2013). Together, the reliable and sustained cooperativity of multiple transporters is required to ensure proper SV (re-)filling and to dynamically regulate inhibitory synaptic strength.

In the auditory system, especially in the brainstem nuclei, synaptic transmission occurs with remarkable robustness, even during sustained high-frequency stimulation at 100 Hz and above (Lujan and von Gersdorff, 2017). A microcircuit and an established model system for the analysis of inhibitory synapses is formed by glycinergic neurons in the medial nucleus of the trapezoid body and postsynaptic neurons in the lateral superior olive (MNTB and LSO; Brill et al., 2019; Figure 1B). The high resilience, reliability and temporal precision of the MNTB-LSO connection is achieved through a high quantal content (*m*, number of SVs released by an AP) and effective replenishment mechanisms (Krächan et al., 2017). During sustained 100-Hz trains, 6,000 release events can occur within 60 s, involving the exocytosis of ~24,000 SVs from the axon terminals of a single MNTB neuron terminating on an LSO neuron (minimal stimulation; young adult mice; Müller et al., 2022). At the end of the 60-s challenge period (s_50-60_), when synaptic depression is maximal, steady-state release is still ~350 SVs/s. One must hypothesize that the synaptic indefatigability requires efficient replenishment, as all SVs, including those from the reserve pool, are used in this severe challenge conditions (Rizzoli, 2014). At steady state, SV fusion and SV replenishment are in equilibrium.

To assess the efficacy of SV replenishment and the underlying molecular mechanisms at a high-fidelity inhibitory synapse, we here performed electrophysiological experiments in mouse brain slices and generated presynaptic AP trains at the MNTB-LSO inputs to deplete the RRP and to assess sustained neurotransmission. Long-lasting and high-frequency stimulation was used (up to 4 min and 200 Hz; Figure 1C). We pharmacologically interfered with SV transmitter loading by adding the plecomacrolide antibiotic bafilomycin (Bafi), a high-affinity inhibitor of V-ATPase-mediated H^+^ transport (Bowman et al., 1988; Dröse and Altendorf, 1997; Zhou et al., 2000; Forgac, 2007). Bafi blocks the reacidification of newly endocytosed SVs. In complementary experiments, we used folimycin (Foli; Muroi et al., 1993; Sara et al., 2005; Groemer and Klingauf, 2007). Unexpectedly, the pharmacological treatments had only moderate effects on SV replenishment and did not abolish synaptic transmission, despite the high drug concentrations and very intense stimulation conditions used. The resistance to pharmacological treatment suggests effective SV (re-)filling mechanisms at MNTB-LSO synapses that are not solely dependent on V-ATPase activity. Rather, another mechanism appears to coexist that provides an H^+^ source for VIAAT and thus helps to generate SVs with a sufficiently high glycine content. In the Discussion section, we provide some thoughts on the nature of this source.

## Materials and methods

### Animals and ethical approval

Animal breeding and experiments were approved by the regional councils of Rhineland-Palatinate according to the German Animal Welfare Act (TSchG §4/3) and followed the guidelines for the protection and welfare of laboratory animals. C57BL/6 J mice were bred in the animal facilities of the University of Kaiserslautern-Landau, and both sexes were analyzed at postnatal days (P) 11 ± 1.

### Electrophysiology

Coronal brainstem slices containing the MNTB and the LSO were prepared in accordance with the methodology previously described by Hirtz et al. (2012). Slices were stored at room temperature (RT) in carbonated artificial cerebrospinal fluid (ACSF), which consisted of the following components (in mM): 125 NaCl, 2.5 KCl, 1.25 NaH_2_PO_4_, 2 sodium pyruvate, 3 myo–inositol, 0.44 L (+) ascorbic acid, 25 NaHCO_3_, 10 glucose, 1 MgCl_2_, 2 CaCl_2_. The solution had an osmolality of 290 ± 10 mOsm/L and was bubbled with carbogen to achieve a pH of 7.4. Individual slices were transferred into the recording chamber on an upright microscope (Nikon Eclipse E60FN, Minato, Japan) equipped with infrared-differential interference contrast optics (Nikon 4x CFI Achromat, 0.1 ∞; 60x CFI Fluor W, 1.00 W ∞; Zeiss Fluar CFI 5x/0.25 ∞/0.17; Olympus LUMPlanFL N 60x/1.00 W ∞/0/FN26.5) and an Orca-05G camera (Hamamatsu, Hamamatsu City, Japan). Whole-cell recordings were performed at 35 ± 1°C (Slice Mini Chamber I equipped with temperature controller TC07, Luigs & Neumann, Ratingen, Germany). LSO principal neurons were identified by their fusiform somata, and this identification was subsequently confirmed through an examination of their electrophysiological properties (Sterenborg et al., 2010). Patch pipettes and stimulation pipettes (GB150F-8P with filament, Science Products, Hofheim am Taunus, Germany) were constructed with a P-1000 horizontal puller (Sutter Instruments, Novato, CA, United States). The input resistance of the patch pipettes ranged from 2.5 to 4.5 MΩ when filled with an internal solution containing the following components (in mM): 140 K-gluconate, 10 HEPES, 5 EGTA, 1 MgCl, 2 CaCl_2_, 2 Na_2_ATP, 0.3 Na_2_GTP. The pH was 7.3 and the osmolality was 280 ± 10 mOsm/L. The recordings were conducted using an EPC10 amplifier and controlled with PatchMaster software (HEKA, Lambrecht/Pfalz, Germany). Recordings were sampled at 20 kHz and low-pass filtered at 2.9 kHz. The liquid junction potential (15.4 mV) was corrected online. Series resistance amounted up to 20 MΩ and compensated between 40 and 60%. If the input resistance changed by >30%, the recording was discarded. Stimulation pipettes (tip diameter ~10 μm), filled with ACSF and connected to a stimulus isolator (STG4002; MultiChannel Systems; Reutlingen; Germany), were placed either at the lateral edge of the MNTB (orthodromic stimulation) or at the medial edge of the LSO (antidromic stimulation). In both cases, 100-μs long electrical current pulses were applied to evoke inhibitory postsynaptic currents (eIPSCs) or APs, respectively. At a holding potential of −70 mV, eIPSCs were outward currents. Stimulus amplitudes were adjusted to values which yielded stable maximal eIPSCs during low-frequency stimulation (0.5 Hz to avoid synaptic depression). For APs, once the rheobase was reached, the stimulus amplitude was doubled. We employed three different stimulation protocols (Figure 1): Protocol 1 started with a 1-Hz|60-s stimulation period to establish a baseline value (BL, average eIPSC peak amplitude = 100%). Thereafter, MNTB-LSO synapses were electrically stimulated for 60-s at various frequencies in ascending order (Challenge period: Chal 1-4; 10, 50, 100, 200-Hz). Each challenge period was immediately followed by a 60-s period at 1-Hz (Recovery period: Recov1-4). A total of 21,900 stimuli were applied. A 6-s pause was introduced for data storing after BL and between the last stimulus of the recovery period and the 1st stimulus of the subsequent challenge period. Protocol 2 started with a 1-Hz|60-s BL, followed by a 4-min | 100-Hz stimulation (Chal 1-4). The challenge period was followed by a 180-s period at 1-Hz (Recov1-3). A total of 24,240 stimuli were applied. A 6-s pause was introduced for data storing after BL and between the last stimulus of the recovery period and the 1st stimulus of the subsequent challenge period. Protocol 3 consisted of a 1-Hz|60-s period to assess the BL, followed by 60-s challenging periods at 50-Hz and 100-Hz stimulation (Chal1-2). Each challenge period was followed by a 60-s period at 1-Hz to assess recovery from depression (Recov1-2). Thus, a total of 9,180 stimuli were applied. A 6-s pause was introduced for data storing after the BL period and between the last stimulus pulse of Recov1 and the 1st stimulus pulse of Chal2.

### Immunohistochemistry

Lethally anesthetized mice (10% ketamine, 220 μg/g body weight plus 2% xylazine, 24 μg/g body weight, intraperitoneal) were transcardially perfused with 100 mM phosphate-buffered saline (PBS, pH 7.4) at RT, followed by ice-cold 4% paraformaldehyde (PFA) for 20 min (Ecoline VC-360 pump, IsmaTec, Chicago, United States). Brains were removed from the skull, postfixed for 1 h in 4% PFA (4°C) and stored in 30% sucrose-PBS at 6°C overnight. 30-μm-thick coronal brainstem slices were cut with a sliding microtome (HM 400 R, MICROM) and transferred to 15% sucrose-PBS, followed by three rinses in PBS (30 min, RT). For NHE6, a heat-induced epitope retrieval protocol was used prior to antibody treatment. For this, slices were immersed in 10 mM sodium citrate buffer (pH 6.0) plus 0.05% Tween 20 (Roth, Karlsruhe, Germany) and heated at 95°C for 20–40 min. They were then returned to RT, followed by three rinses in PBS (5 min, RT). Slides were applied free-floating at 4°C for 1 in blocking solution (0.3% Triton-X-100, 10% goat serum, 2% bovine serum albumin (BSA) in PBS). Antibodies against GlyT2 (1:200, host: mouse, Synaptic Systems, Göttingen, Germany) and NHE6 (1:200, host: rabbit, Sigma Aldrich, St. Louis, United States) were applied free-floating at 4°C overnight in carrier solution (0.3% Triton-X-100, 1% goat serum, 1% BSA in PBS), followed by three rinses in PBS (30 min, RT). Slices were then incubated in the dark for 2 h in carrier solution and secondary antibody (1,1,000; goat-anti-mouse, Alexa Fluor 488; goat-anti-rabbit, Alexa Fluor 647, Thermo Fisher Scientific, Waltham, United States), followed by three rinses in PBS (30 min, RT). Slices were mounted on gelatin-coated glass slides and covered with mounting medium containing 2.5% DABCO (1,4-diazabicyclo[2.2.2]octane; Sigma-Aldrich, St. Louis, MO, United States). Images were acquired with a TCS SP5 X confocal microscope equipped with an HCX PL APO 63 *×* oil objective (Leica Microsystems, Wetzlar, Germany). For quantitative analysis, intensity profiles at inhibitory synapses were analyzed using Fiji ImageJ 1.54 as described previously (Fischer et al., 2019). Line scans were drawn from the center of the soma across membrane-associated GlyT2 signals. Eight intensity profiles were obtained per cell. In total, two P11 animals, 16 cells and 128 intensity profiles were determined. The position of maximum GlyT2 signal was set to zero, with negative and positive scan positions representing intracellular and extracellular locations, respectively. A rolling ball background subtraction (radius 20) was applied to images in Fiji ImageJ 1.54 (Schindelin et al., 2012), followed by brightness adjustment. To assess colocalization of the immunosignals, Fiji’s Coloc2 plugin was used to determine the Pearson correlation coefficient and the Costes’ test (P_Costes_), a measure of reliability (Costes et al., 2004). P_Costes_ = 1 indicates >95% certainty that colocalization exists.

### Pharmacology

#### Application of bafilomycin or folimycin to inhibit V-ATPase

Bafilomycin A1 (Bafi; Abcam, Cambridge, United Kingdom or Thermo Fisher Scientific, Waltham, United States) was dissolved in ethanol (EtOH) to reach a stock concentration of 2 mM. Folimycin (Foli; a.k.a. concanamycin A) was dissolved in dimethyl sulfoxide (DMSO) to a stock concentration of 2 mM (Abcam, Cambridge, United Kingdom). Notably, the IC_50_ of Bafi and the K_i_ of Foli are in the (sub-)nanomolar range. The stock solutions were stored at −20°C. On the day of the experiment, the stock solutions were diluted in ACSF such that the final concentration was 2 or 5 μM for Bafi and 1 μM for Foli. The pharmacological analysis began after a 10-min wash-in period (referred to as Bafi10 and Foli10). A third recording session was conducted after waiting at least 11 and up to 41 min (referred to as Bafi30 or Foli30). To maximize their effects, the drugs were continuously perfused during the recording sessions. This strategy differed drastically from the 3-min Bafi exposure period used in a study on glutamatergic calyx of Held synapses (Hori and Takahashi, 2012). It also differed considerably from the <2-min application via a picospritzer used in a study on isolated and cultured hippocampal neurons that had formed autapses (Ikeda and Bekkers, 2009).

#### Application of EIPA to inhibit Na^+^/H^+^ exchangers (NHEs)

The amiloride derivative EIPA was dissolved in DMSO at 100 mM and stored at −20°C (ethylisopropylamiloride; Tocris, Bristol, United Kingdom). Because EIPA is hydrophobic, it permeates cells. EIPA reportedly blocks NHEs, the electroneutral Na^+^/H^+^ exchangers (Alexander et al., 2011; Li et al., 2020). On the day of the experiment, EIPA was diluted in ACSF to a final concentration of 100 μM (same concentration as in Goh et al., 2011; Huang and Trussell, 2014; Li et al., 2020) and bath-applied for 10 min before recording commenced (referred to as EIPA10).

### Data analysis

Foot-to-peak eIPSC amplitudes were analyzed with a custom-written plugin (Dr. Alexander Fischer, University of Kaiserslautern-Landau) implemented in IGOR Pro 6.37 or 9.01 (WaveMetrics Inc., Lake Oswego, OR, United States). Negative eIPSC amplitudes were set to zero. Data were then normalized to BL (mean eIPSC peak amplitude = 100%; Galarreta and Hestrin, 1998).

Recovery behavior was calculated by three means: (a) RecovA: last 10 eIPSCs recovery vs. BL (= 100%); (b) RecovB: last 10 eIPSC recovery vs. last 10 eIPSC of previous challenge; (c) Fractional recovery (FR):


$$\mathrm{FR}=\frac{\text{last}\;10\;\text{eIPSC recovery}-\text{last}\;10\;\text{eIPSC challenge}}{\text{baseline}\;(=100\%)-\text{last}\;10\;\text{eIPSC challenge}}$$

sIPSCs were analyzed during 1-Hz|60-s stimulus periods (BL and recovery periods) and manually detected using Clampfit 10.2.0.14 (Molecular Devices, CA, United States). Histograms of the sIPSCs peak amplitudes were generated and fitted with Gaussian curves. The quantal size (*q*) was determined by a Gaussian fit from 0 pA to the bin after the first local maximum (see Krächan et al., 2017; bin width 5 pA, in some cases 3 pA). The cumulative current evoked through complete release of the *RRP* (*I_RRP_*) was assessed by forward extrapolation of a linear fit through eIPSC_1-5_ obtained at 100 Hz (Elmqvist and Quastel, 1965). The number of SVs in the *RRP (N_RRP_)* was determined as the ratio *I_RRP_/q*. The quantal content (*m*) was obtained as eIPSC_1_/*q_Recov2_*. The release probability (*Pv*) was calculated as *I_RRP_/eIPSC_1_*. The transfer rate (*TR*) was evaluated as described (Müller et al.). Briefly, *TR* was assessed from the slope of a line fit through the last 10 s (s_50-60_) of the cumulative eIPSC curve in a challenge period.

### Statistics

Statistical analysis was performed with GraphPad Prism 9 (GraphPad Software). Data are presented as mean ± SEM. For distribution analysis, the Kolmógorov–Smirnov test was used. Normally distributed data were compared using paired or unpaired two-tailed *t*-tests. If the distribution was not normal, the Wilcoxon rank-sum test or the Mann–Whitney U test was used. Details on the specific tests used are provided in the Supplementary Tables S1–S12. The *post hoc* tests were corrected using Bonferroni’s adjustments where applicable (*α*_adjusted_ = α/k, where α is the significance level and k the number of comparisons). Since k was maximally 2, the adjusted significance levels are as follows:

**Table:** 

α	α_adjusted_	Notation *p*-value < α or α_adjusted_
0.05	0.025	*
0.01	0.005	**
0.001	0.0005	***

## Results

The stimulus regime for the first series of experiments (named “Protocol 1”) consisted of an initial 60-s|1-Hz period to assess the baseline level (BL) of eIPSC peak amplitudes, followed by four 60-s challenge interposed by 60-s recovery periods. Challenge frequencies were offered in ascending order (10, 50, 100, 200 Hz; Figure 1C). In total, Protocol 1 lasted 9 min and included 21,900 stimulus pulses. In a first round, the synaptic behavior was assessed in ASCF (Ctrl). Then the V-ATPase inhibitor Bafi (2 μM) was bath-applied for 10 min before a second round was performed on the same neuron (Bafi10). Finally, another neuron was patch clamped in the same slice and analyzed in a third round (≥ 30 min drug treatment named “Bafi30”).

**Figure 2:**
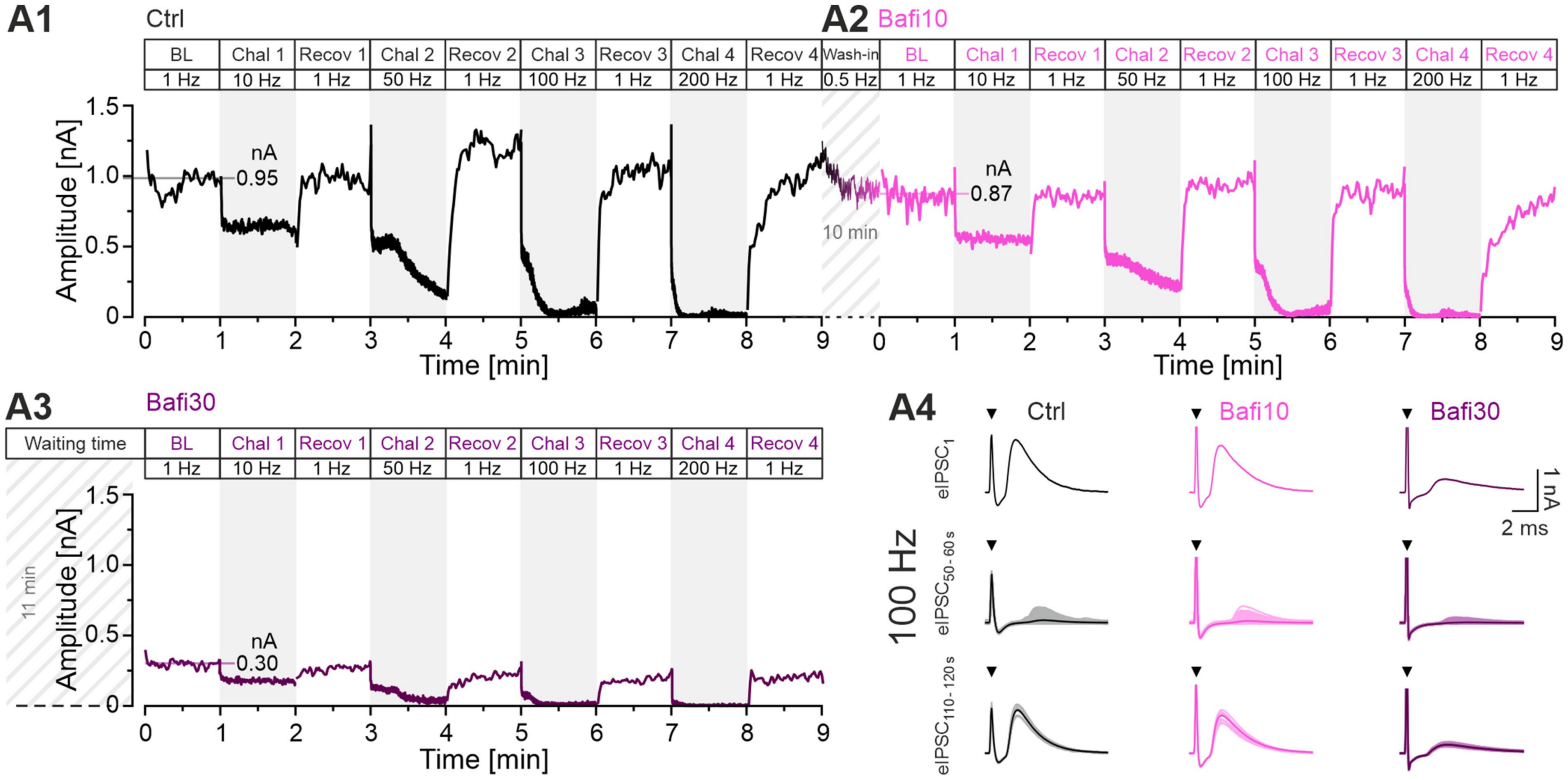
MNTB-LSO inputs function reliably during sustained high-frequency stimulation and recover robustly and precisely from synaptic depression. Inhibition of V-ATPase activity with 2 μM Bafi does not completely abolish information transfer. **(A1)** Time course of absolute peak eIPSC amplitudes obtained with Protocol 1 from a P11 LSO neuron under Ctrl conditions (black, ACSF): BL (0–1 min; dotted horizontal line indicates mean peak amplitude), four periods of 60-s challenge (Chal; gray regions) and 1 Hz|60-s recovery (Recov; white regions; 1–9 min), four challenge frequencies in ascending order (10, 50, 100, 200 Hz, see Figure 1C) **(A2,A3)**. Same as **(A1)**, but for Bafi10 (2 μM, 10-min wash-in, magenta) **(A2)** and Bafi30 (2 μM, ≥ 30-min treatment, purple) **(A3)**. A 6-s pause was introduced after the BL and each 60-s recovery period. Diagonally striped regions indicate perfusion periods of 2 μM Bafi (time indicated in the figure; after perfusion, the drug was maintained in the bath throughout the entire recording period). Time courses are simple moving averages over three (1 Hz) or nine (10–200 Hz) data points to smooth out short-term fluctuations and highlight longer-term trends. Data in **(A1,A2)** are from the same neuron, whereas **(A3)** is from a different neuron. The number of stimulus pulses in each panel is 21,900. **(A4)** Representative eIPSCs for the three cohorts during Chal 3 and Recov 3, showing eIPSC_1_ and the overlay of individual eIPSCs and graphical means for eIPSC_s50-60_ and eIPSC_s110-120_ (lightly and darkly shaded). Arrows indicate stimulation artifacts. Note transmission failures in the 100-Hz traces for eIPSCs during s_50-60_ (for AP fidelity aspects, see Figures 10, 11; Müller et al., 2022).

The synaptic performance of an example neuron in the Ctrl situation is illustrated in Figure 2A1. The synaptic depression, the sustained performance, and the recovery behavior are evident, and the results are consistent with previous findings (e.g., Kramer et al., 2014; Krächan et al., 2017). The mean BL eIPSC peak amplitude was calculated to be 951 pA, and BL eIPSC_1_ was 1,189 pA, thus showing an overshoot of 25%. We have previously referred to such an overshooting *in vitro* behavior as “manic.” It occurs after “long” rest intervals of several seconds (Friauf et al., 2015). During the 10-Hz challenge, the peak amplitudes decreased to 629 pA. During the subsequent recovery period, they quickly and reliably returned to BL (942 pA or 99%). As expected, synaptic depression was greater during the 50-Hz challenge, reaching a final level of 178 pA. Recovery was again rapid and robust, even exceeding BL (1,199 pA = 126%). During the 100-Hz challenge, amplitudes dropped massively to 63 pA. Again, recovery was robust, with the mean amplitude of the last 10 s (s_50-60_) reaching 1,023 pA (108% of BL). The final 200-Hz challenge resulted in virtually complete abolition of transmission (13 pA), but recovery returned values to 1,080 pA (114% of BL).

During the subsequent 10-min wash-in of Bafi, a new BL of 865 pA was achieved (Figure 2A2). This value was 9% lower than the Ctrl value. A decrease in steady-state amplitudes (s_50-60_) was evident during 10-Hz and 50-Hz challenge (550 and 229 pA, respectively). At 100-Hz, there was a plateau after the initial rapid decline, as in Ctrl, and amplitudes were 49 pA during s_50-60_. The final 200-Hz challenge resulted in a massive depression to 12 pA, again implying virtually completely abolished transmission. As in the Ctrl scenario, amplitudes returned very close to BL levels during recovery periods (10 Hz: 867 pA = 100%; 50 Hz: 977 pA = 113%, 100 Hz: 899 pA = 104%; 200 Hz: 827 pA = 96%).

The ability to recover from depression after the severe challenge during Protocol 1 suggests that the 10-min|2-μM Bafi treatment may have been insufficient to strongly inhibit transmission at MNTB-LSO synapses. To address this issue, we assessed the synaptic performance in a second neuron after Bafi was washed in for ≥ 30 min to increase the chance of efficient V-ATPase inhibition. The example neuron showed drastically smaller eIPSCs than the Ctrl/Bafi10 neuron (Figure 2A3). For example, the BL was 300 pA, only 32% of the Ctrl. This implies a smaller RRP and/or a lower q, most likely due to efficient V-ATPase inhibition. s_50-60_ depression levels during the four challenge periods were 175, 37, 13 and 3 pA, respectively. Although these values indicate a complete collapse of synaptic transmission at the two highest frequencies, especially at 200 Hz, Bafi30 synapses managed to recover partially in each case (Figure 2A4 shows individual eIPSCs at 100 Hz). Recovery after the 100-Hz and 200-Hz challenge reached 206 and 221 pA, respectively, (69 and 74% of the 300 pA BL). These single-cell results provide some preliminary evidence that V-ATPase inhibition was incomplete or that there may be another source of H^+^ ions fueling VIAAT.

### Cohort data confirm that sustained transmission is not affected upon 10-min wash-in of bafilomycin (Bafi10), yet it is reduced after 30-min treatment (Bafi30)

**Figure 3:**
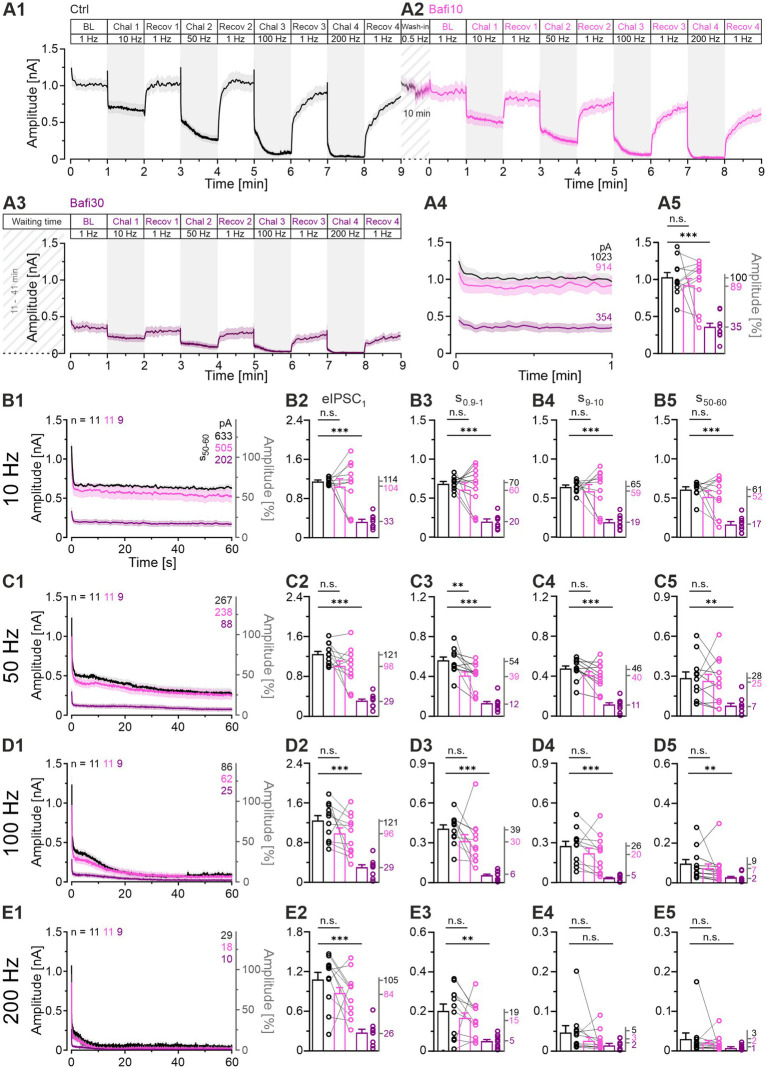
Ongoing synaptic transmission is not affected by 10-min wash-in of 2 μM Bafi (Bafi10) at MNTB-LSO inputs. In contrast, treatment for ≥30 min (Bafi30) leads to reduced amplitudes. **(A1,A2)** Time course of eIPSC mean ± SEM peak amplitudes for Ctrl (black) and Bafi10 (magenta) obtained from paired recordings with Protocol 1 (see Figure 1C). **(A3)** Time course for Bafi30 (purple). Data in **(A3)** are from a different neuron than in **(A1,A2)**. The BL mean of the Ctrl was set to 100%. Diagonally striped regions indicate perfusion periods of 2 μM Bafi. A 6-s pause was introduced after the BL and each 60-s recovery period. **(A4)** Close-up of the BL traces. Mean values are shown on the right. **(A5)** Statistics for BL analysis (mean: Ctrl: 1,023 pA = 100%, Bafi10: 914 pA = 89%; Bafi30: 354 pA = 35%). **(B1)** Time course of absolute and normalized eIPSC peak amplitudes during 10-Hz|60-s challenge. Upper right numbers indicate the mean amplitudes during s_50-60_. **(B2–B5)** Statistics at four time points (eIPSC_1_, s_0.9-1_, s_9-10_, s_50-60_). Mean percentages are shown on the right y-axis. **(C–E)** Same as B, but for 50 Hz **(C1–C5)**, 100 Hz **(D1–D5)** and 200 Hz **(E1–E5)**. Note the different amplitude scaling in **(B2–E5)**; the variables on the left and right y-axes are the same as in **(B1)**. Time courses are simple moving averages over three (1 Hz) or nine (10–200 Hz) data points, with SEM lightly shaded. Ctrl and Bafi10, *n* = 11; Bafi30, *n* = 9. See Supplementary Table S1 for details, including statistics.

To corroborate the anecdotal evidence derived from the two example neurons shown in Figure 2, we proceeded to examine the cohort behavior (*n* = 11, 11, 9; Figure 3). The BL values were found to be similar between the Ctrl and Bafi10 cohort (mean: 1,023 vs. 914 pA or 100:89; Figures 3A1,A2,A4,A5). In contrast, Bafi30 values were significantly reduced, reaching only 35% of the Ctrl (354 pA; Figure 3A3). Furthermore, the time courses of depression and subsequent recovery to the four challenge frequencies were in accordance with those observed for the example neurons (Figures 3A1–A3; compare with Figure 2).

Subsequently, statistical analyses were conducted across the four 60-s challenge periods (eIPSC_1_, s_0.9-1_, s_9-10_, s_50-60_; Figures 3B2–E5; Supplementary Table S1). Only one of the 17 Ctrl_vs_Bafi10 comparisons yielded a statistically distinguishable result (Figures 3A4,B2–B4,C2–C4,D2–D4,E2–E4). In contrast, Bafi30 amplitudes were observed to be significantly lower than those of the Ctrl in 15 out of the 17 comparisons. For example, at s_50-60_ of the 50-Hz challenge, Ctrl amplitudes were ~3-fold higher than Bafi30 amplitudes (267 vs. 88 pA, Figures 3C1,C5). With the harshest 200-Hz challenge, peak amplitudes declined markedly in each of the three cohorts (Figures 3E1–E5). By s_50-60_, they were down to 29, 18 and 10 pA (equivalent to 2.8, 1.8 and 1.0%; Figure 3E5), indicating a near-complete depletion of transmitter release, particularly in the presence of the drug. This may explain the lack of statistical significance shown in panels E4 and E5. To further assess the near-complete collapse of synaptic transmission during the 200-Hz challenge in the two Bafi cohorts, the latest time at which the response amplitude reached ≥ 100 pA/pulse was determined. The values were 4.45, 3.58 and 0.05 s for Ctrl, Bafi10 and Bafi30, respectively (Figure 3E1). This further highlights the drastic difference observed in the Ctrl_vs_Bafi30 comparison, but not for Ctrl_vs_Bafi10 (numbers refer to 890, 716 and 10 stimulus events, respectively). To evaluate the resilience of the synapses, it is noteworthy that each cohort could maintain synaptic transmission for a considerably longer duration during the 100-Hz challenge. The last response exceeding 100 pA was observed at 59.27, 23.78 and 5.75 s for the Ctrl, Bafi10 and Bafi30 cohort, respectively (Figures 3D1–D5).

The sample results confirm that treating the brainstem slices with 2 μM Bafi for 10 min is insufficient to substantially reduce transmission at MNTB-LSO synapses. The postulated drug effect becomes evident only after a longer application time of ≥ 30 min. As a caveat it is also necessary to consider the possibility of a more severe stimulation history and/or rundown effects after this time. The issue is addressed below (see Supplementary Figures S1, S2A1–A3). In any case, the results obtained with Protocol 1 demonstrate that synaptic transmission persists for thousands of stimulus pulses, even in the Bafi30 scenario. It can thus be argued that the pharmacological blockade of V-ATPase, as conducted here, results in an incomplete cessation of SV (re)filling, thus leading to only partial synaptic fatigue. As demonstrated in the section “Recovery from depression is reduced but not abolished by Bafi treatment,” the resilience of the MNTB-LSO synapses is also evidenced by their ability to recover from depression, even in the presence of Bafi.

### Sustained neurotransmission capacity is reduced by bafilomycin but persists

**Figure 4:**
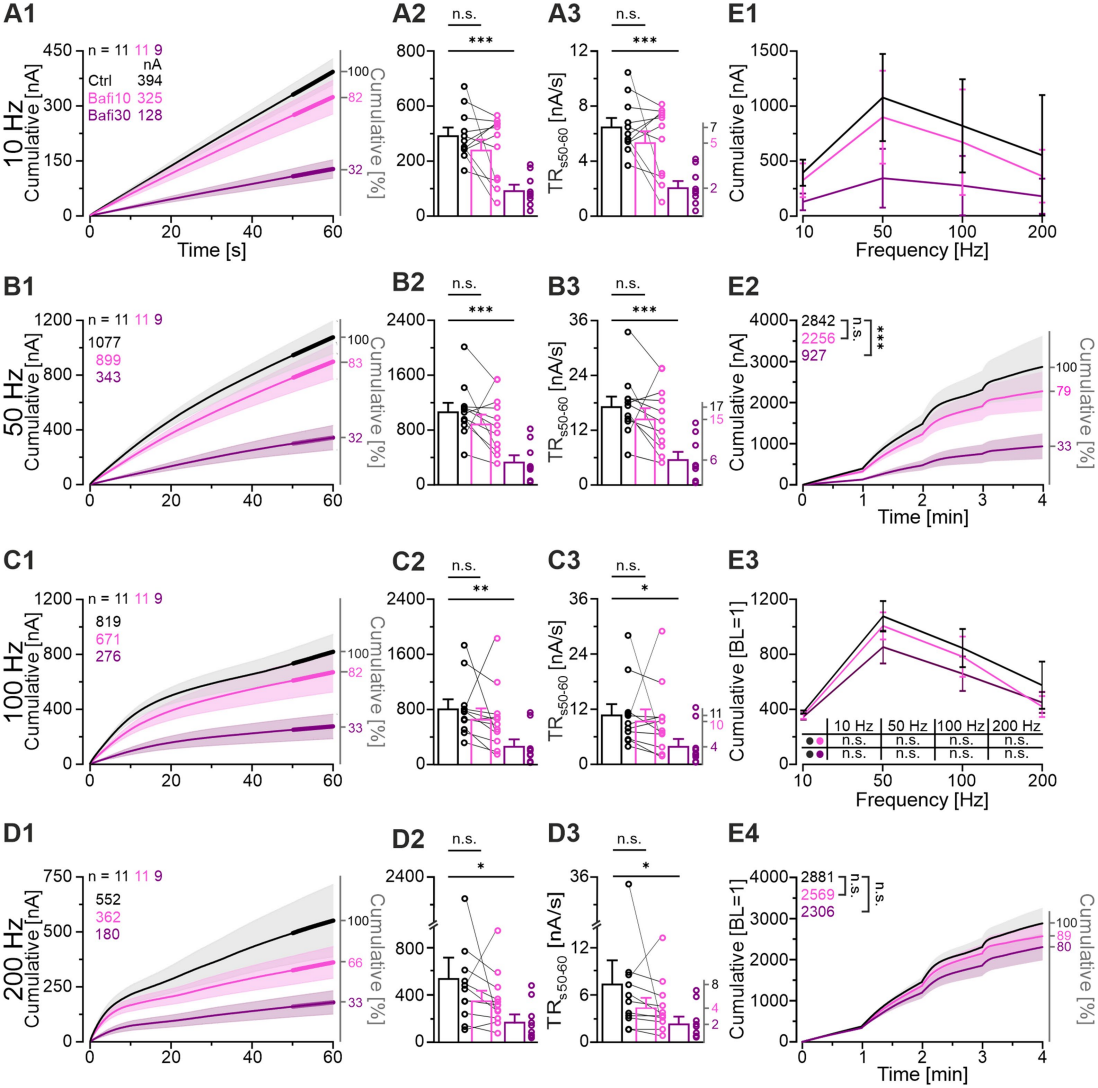
Bafi30 reduces transmission capacity by >60% at MNTB-LSO synapses, but transmission kinetics remain similar. **(A1–D1)** Cumulative current integrals as a function of time at the four challenge frequencies in Ctrl (black), Bafi10 (magenta) and Bafi30 (purple) during Protocol 1 (see Figure 1C). Traces show the incremental increase, and a linear slope corresponds to a steady-state release of constant synaptic strength. Thick lines mark periods when the transmission rate (TR) was quantified (s_50-60_). Upper left numbers indicate the cumulative current amplitudes at eIPSC_60s_. **(A2–D2)** Statistics of eIPSC_60s_ at the four challenge frequencies. Variables on the left y-axis are the same as in **(A1)**. **(A3–D3)** TR during s_50-60_. Mean values are shown on right y-axis. **(E1)** Comparison of the transmission performance between the three cohorts over the four challenge frequencies. **(E2)** Cumulative current integrals summarized over the entire 4 min of challenge (21,600 stimulus pulses). The numbers in the upper left indicate the cumulative absolute current amplitudes at 4 min. **(E3)** As **(E1)**, but normalized so that the BL of each neuron equals 100% (BL = 1). **(E4)** As **(E2)**, but after normalization. Time courses are mean cumulative amplitudes (SEM lightly shaded). Ctrl and Bafi10, *n* = 11; Bafi30, *n* = 9. See Supplementary Table S2 for details, including statistics.

The above analysis included only some aspects of the synaptic performance, namely windows with a total duration of 11.1 s. To assess the transmission capacity during the entire 60 s for each challenge period, we determined the cumulative eIPSC amplitude (Müller et al., 2022). Each cohort showed the highest value at 50 Hz, indicating that this is the “best transmission frequency” (Figures 4A1,B1,C1,D1,E1,E3). Ctrl synapses generated a cumulative amplitude of 1,077 nA at this frequency, whereas Bafi10 and Bafi30 values were 899 and 343 nA, respectively (83 and 32% of Ctrl; Figures 4B1,B2). No statistically significant difference occurred in any of the four Ctrl_vs_Bafi10 comparisons, whereas significance was evident in each Ctrl_vs_Bafi30 comparison (Figures 4A2,B2,C2,D2; Supplementary Table S2). Notably, synapses treated with Bafi30 performed at ~1/3 of the Ctrl level at each challenge frequency.

To quantify neurotransmission during the steady-state phase of the challenge periods, and thus the continuous replenishment rate, we analyzed the transmission rate during the last 10 s of the challenge period (TR_50-60_, [nA/s]). The results confirmed those obtained for the entire 60-s periods, namely a statistically significant difference for Ctrl_vs_Bafi30 at each frequency, but no difference for Ctrl_vs_Bafi10 (Figures 4A3,B3,C3,D3). For example, the Ctrl had a mean TR_50-60_ of 17.3 nA/s at 50-Hz challenge, compared to Bafi10 and Bafi30 values of 14.6 and 5.7 nA/s, respectively (ratios: 100:84:33; Figure 4B3). Very similar ratios were obtained at the three other challenging frequencies (10 Hz: 100:82:32; 100 Hz: 100:88:34; 200 Hz: 100:60:32).

We also analyzed the overall transmission by determining the cumulative eIPSC amplitude over all four challenge periods, thereby assessing the response strength to 21,600 pulses. On average, the Ctrl cohort was able to elicit 2,842 nA (on average 132 pA/event), whereas the Bafi10 and Bafi30 values were reduced to 79 and 33%, respectively (2,256 and 927 nA; Figure 4E2). Again, the difference was statistically significant for Ctrl_vs_Bafi30, but not for Ctrl_vs_Bafi10.

Taken together, the results obtained from the analysis of overall transmission further support our conclusion that synaptic transmission at MNTB-LSO synapses is substantially reduced only after prolonged Bafi application (≥ 30 min).

### Normalized data show similar performance across cohorts

Since the Bafi30 BL was ~3-fold lower than the Ctrl BL (354 vs. 1,023 pA; Figures 3A4,A5), we wondered whether this initial difference alone, present before the first challenge period, could explain the transmission differences described so far. To address this question, we set the BL of each neuron to 100% (BL = 1), allowing us to assess its relative performance. For the 10-Hz challenge, the mean final cumulative value was 381 for the Ctrl, very close to the Bafi10 and Bafi30 values of 348 and 344, respectively (Figure 4E3). Thus, the relative performance was remarkably similar between cohorts (100: 91: 90). Values for the 50-Hz challenge were 1,079, 1,009 and 854 BL (100: 94: 79), and confirming results were obtained at 100 Hz (847, 783 and 659 BL; 100: 92: 78) and 200 Hz (575, 419 and 449 BL; 100: 73: 78). None of the eight comparisons reached statistical significance (Figure 4E3). Finally, the normalized current integrals across 4-min challenging also showed statistically indistinguishable performances (Figure 4E4). Together, these results imply a weaker transmission strength with prolonged Bafi treatment (Bafi30), but similar transmission kinetics.

### Recovery from depression is reduced but not abolished by Bafi treatment

**Figure 5:**
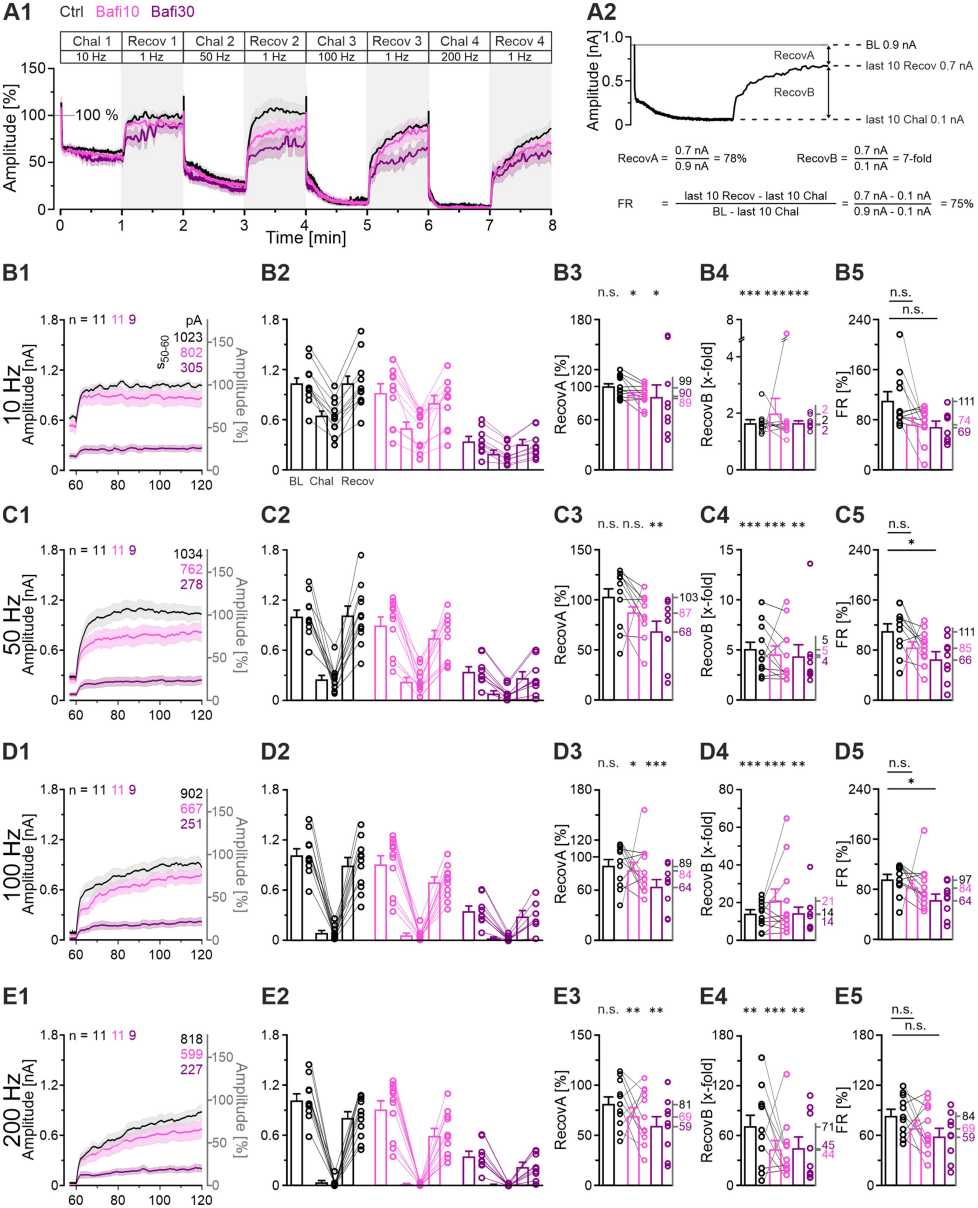
Recovery from depression is very efficient at Ctrl and Bafi10 synapses but reduced in Bafi30. **(A1)** Time course of normalized eIPSC amplitudes (mean ± SEM) in Ctrl (black), Bafi10 (magenta) and Bafi30 (purple) during Protocol 1 (see Figure 1C). The BL mean of the Ctrl, Bafi10 and Bafi30 was set to 100% (gray horizontal line). A 6-s pause was introduced after BL and after each 60-s recovery period for data storage. **(A2)** Recovery behavior was evaluated using three methods: (1) RecovA: last 10 eIPSCs of recovery vs. BL (= 100%); (2) RecovB: last 10 eIPSCs of recovery vs. last 10 eIPSCs of previous challenge; (3) FR:$\frac{\text{last}\;10\;\text{eIPSCs Recov}-\text{last}\;10\;\text{eIPSCs Chal}}{\mathrm{BL}\;(=100\%)-\text{last}\;10\;\text{eIPSCs Chal}}$. Example calculations are provided for clarity. **(B1)** Time course of absolute and normalized amplitudes during 1-Hz|60-s recovery after 10-Hz challenge. Numbers in the upper right indicate absolute mean amplitudes during s_50-60_. **(B2)** Quantification at the end of the BL, challenge, and recovery periods (last 10 eIPSCs). Variables on the left y-axis are the same as in **(B1)**. **(B3–B5)** Statistics for RecovA **(B3)**, RecovB **(B4)**, and FR **(B5)**. Means are shown on the right y-axis. **(C–E)** Same as **(B)**, but for recovery after 50-Hz **(C1–C5)**, 100-Hz **(D1–D5)**, and 200-Hz challenge **(E1–E5)**. Variables on the left y-axis in **(C2–E2)** are the same as in **(C1)**. Note the different amplitude scaling in **(B3–E5)**. Time courses are simple moving averages over three (1 Hz) or nine (10–200 Hz) data points (SEM lightly shaded). Ctrl and Bafi10, *n* = 11; Bafi30, *n* = 9. See Supplementary Table S3 for details, including statistics.

Effective SV (re-)filling is of great importance, not only for the robustness of neurotransmission during ongoing activation, but also for the recovery from synaptic depression. Accordingly, we examined the recovery behavior in the context of V-ATPase inhibition and compared it to the Ctrl situation (Figure 5; Protocol 1). After normalization of the eIPSC peak amplitudes of each neuron to its own baseline (BL = 100%), considerable overlap of the challenge-related traces was observed across cohorts, whereas the recovery traces showed greater divergence (Figure 5A1). In particular, the Bafi30 cohort exhibited lower values (Figures 5B1,C1,D1,E1).

Recovery was quantified in three ways (Figures 5A2,B2,C2,D2,E2; cf. Brill et al., 2019; Brill et al., 2021). The *RecovA* metric assesses the ability of the system to accurately return to the BL level. The Ctrls were able to do so after each challenge period (Figures 5B3,C3,D3,E3; end values 99, 103, 89 and 81%). Bafi10 was successful in three of four instances (Figures 5B3,C3,D3) and thus only slightly inferior. In contrast, Bafi30 was unsuccessful in three instances (Figures 5C3,D3,E3), implying replenishment impairment.

The *RecovB* metric focuses on the fold increase from the steady-state depression level (the last 10 challenge events), and thus does not consider the BL. For each cohort, the *RecovB* behavior was statistically significant (Figures 5B4,C4,D4,E4). The results demonstrate the ability of MNTB-LSO synapses to recover from depression, even after a ≥ 30-min treatment with the V-ATPase blocker.

We also determined the fractional recovery (*FR*). The Bafi30 cohort showed significantly lower *FR* values than the Ctrl at two frequencies (Figures 5C5,D5; Supplementary Table S3). In contrast, no statistically significant difference was observed between Bafi10 and Ctrl at any frequency (Figures 5B5–E5). Collectively, these findings provide further evidence that the effects of 2 μM Bafi were relatively subtle and became effective only after prolonged treatment (≥ 30 min) and challenge frequencies ≥ 50 Hz.

As a final step, we compared the time courses of recovery between cohorts. For this purpose, we calculated the weighted time constant τ_w_ after fitting the curves with a biexponential function. During recovery after the 10-Hz challenge, τ_w_ values were low, implying fast replenishment (Ctrl: 2.1, Bafi10: 2.1, Bafi30: 2.1 s; ratios 100:100:100). They were substantially higher for the recovery after 100-Hz (Ctrl: 23.7, Bafi10: 23.8, Bafi30: 34.4 s; ratios 100:100:145) and 200-Hz challenge (Ctrl: 35.2, Bafi10: 34.9, Bafi30: 39.5 s; ratios 100:99:112). The observation that the ratios differed less than 1.5-fold in each comparison suggests that blocking the V-ATPase activity did not affect the recovery kinetics.

### Delayed and gradual reduction of the quantal size (*q*) in the presence of Bafi

**Figure 6:**
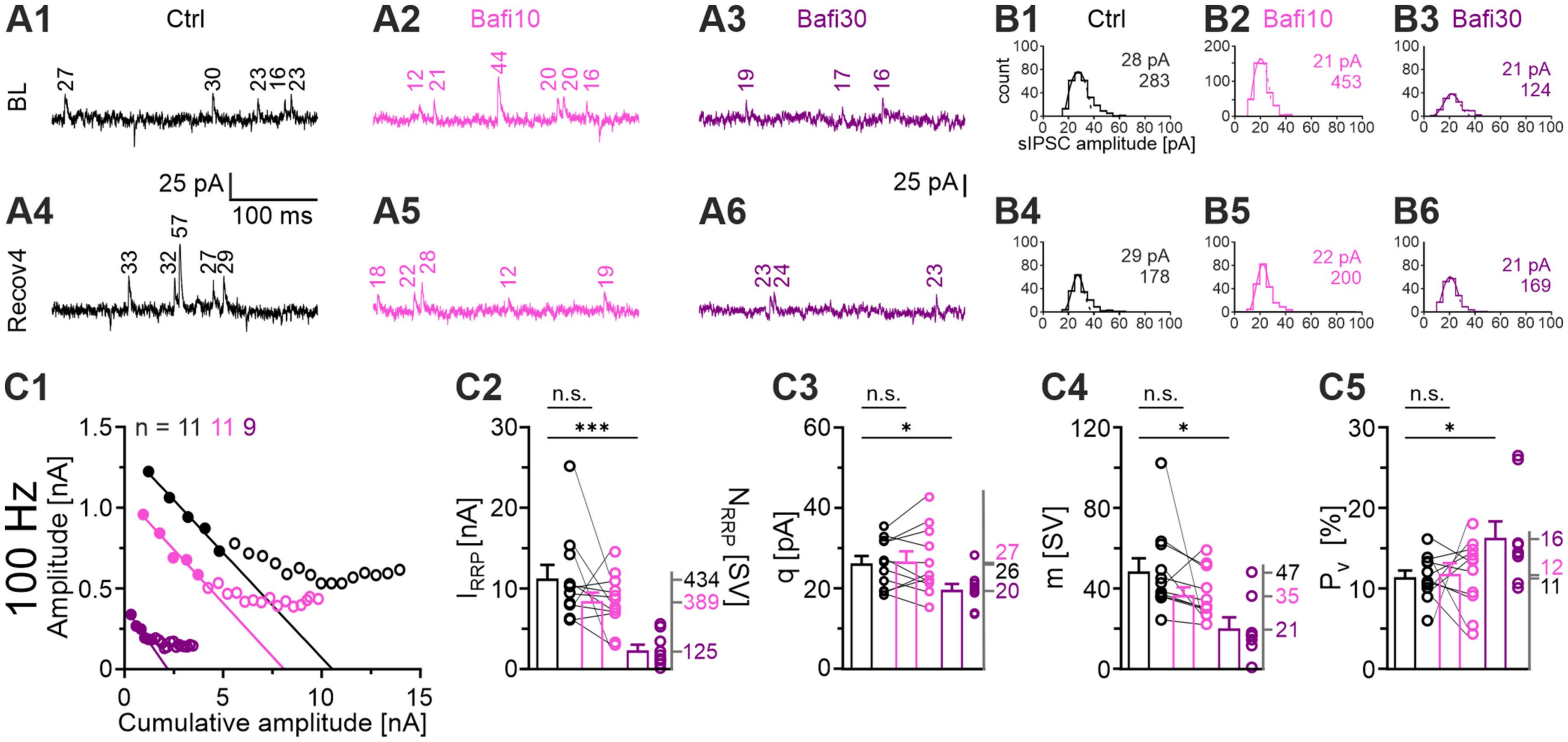
Synaptic parameters are altered by Bafi30: *q*, *I_RRP_*, and *m* are reduced, whereas *P_v_* is increased. **(A)** Original sIPSC traces for Ctrl (black), Bafi10 (magenta), and Bafi30 (purple). **(A1)** sIPSC amplitudes during BL **(A1)** and Recov4 **(A4)** for Ctrl. Same as **(A1,A4)**, but for Bafi10 **(A2,A5)**, and Bafi30 **(A3,A6)**. Numbers indicate the sIPSC amplitudes. **(B)** Estimation of *q*. **(B1,B4)** Distribution histograms of sIPSC amplitudes from current traces in **(A)** during BL **(B1)** and Recov4 **(B4)** for Ctrl. **(B2–B6)** Same as **(B1,B4)**, but for Bafi10 **(B2,B5)** and Bafi30, respectively **(B3,B6)**. A Gaussian curve was fitted from 0 pA to the bin after the first local maximum (dashed curves). Numbers in panels represent *q* values and number of sIPSCs for BL **(B1–B3)** and Recov4 **(B4–B6)** (see Materials and Methods for details). Data for Ctrl and Bafi10 are from one neuron, while data for Bafi30 are from another neuron. **(C)** Analysis of synaptic parameters. **(C1)** Elmqvist & Quastel plots used to determine *I_RRP_*. The stimulation frequency was 100 Hz and a linear fit was applied to eIPSCs_1-5_ of the cohort mean values (see Materials and Methods). **(C2)** Statistics for *I_RRP_*. Means of *N_RRP_* on the right y-axis. **(C3)** Statistics for *q* (obtained from a 300 s period over BL and Recov1-Recov4). Means on the right y-axis. Statistics for *m* **(C4)**, and *P_v_* **(C5)**. Mean values on right y-axis. Note that *N_RRP_* = *I_RRP_*/*q.* Variables on the left y-axis in **(B2–B6)** are the same as in **(B1)**. Ctrl and Bafi10, *n* = 11; Bafi30, *n* = 9. See Supplementary Table S4 for details, including statistics.

Impaired (re-)filling of SVs upon V-ATPase blockade should result in a smaller quantal size (*q*). A complete absence of (re-)filling should even abolish the transmission completely. To test these hypotheses, we determined the peak amplitude of spontaneous IPSCs (sIPSCs) during the baseline and the four recovery periods after challenge (BL and *Recov1*-*Recov4*). Example current traces for the three cohorts during the BL and the *Recov4* periods are shown in Figures 6A1–A6. For each neuron, *q* was determined from the Gaussian distribution of its sIPSC peak amplitudes (Figures 6B1–B6; see Krächan et al., 2017). In the Ctrl_vs_Bafi10 comparison, we found no significant change in *q* (Figure 6C3; Supplementary Table S4). In contrast, a significantly lower *q* value was observed in the Ctrl_vs_Bafi30 comparison (Figure 6C3). The *q* values averaged 26 pA in Ctrl, 27 pA in Bafi10 and 20 pA in Bafi30, corresponding to ratios of 100:104:77 (Figure 6C3). We consider the 23% reduction in Bafi30 to be moderate. The frequency of sIPSCs was determined across the BL and *Recov1-4* periods. Values were 5.7, 5.4 and 3.4 events per second for the Ctrl, Bafi10 and Bafi30 cohorts, respectively (not shown). They correspond to ratios of 100:95:63 and provide further evidence that Bafi treatment did not abolish the (re-)filling process.

After determining the *q* values, we reanalyzed the TR_50-60_ results (cf. Figures 4A3,B3,C3,D3) in order to obtain a vesicle-oriented value of the replenishment rate (SV fusion and SV replenishment are balanced in steady state). At the end of the 50-Hz challenge period, 642 SVs per second were replenished in the Ctrl. The corresponding values for Bafi10 and Bafi30 were 542 and 302 SVs/s, respectively. These results refer to ratios of 100:84:47, and similar ratios were obtained at the other challenge frequencies (10 Hz: 100:82:42; 100 Hz: 100:91:51; 200 Hz: 100:64:44).

Taken together, these results show a delayed and gradual appearance of incompletely filled SVs upon V-ATPase blockade. The moderate reductions in *q* and replenishment rate demonstrate that MNTB axon terminals still maintain a pool of readily releasable SVs whose glycine content is high enough to evoke a postsynaptic current of ~20 pA per SV, i.e., about 70% of the Ctrl level. Thus, refilling of SVs is maintained despite inhibition of V-ATPase activity.

### Bafi application results in reduced readily releasable pool (*RRP*), reduced quantal content (*m*) and increased release probability (*P_v_*)

To analyze synaptic parameters in more detail, we examined *RRP*, *m* and *P_v_*. The forward extrapolation method was used to determine the RRP (Elmqvist and Quastel, 1965) and the 100-Hz challenge period was analyzed (Figure 6D1). Mean values for *I_RRP_* were 11.3, 8.5 and 2.4 nA for Ctrl, Bafi10, and Bafi30, respectively (Figure 6D2). With the *q* values shown above, these numbers correspond to an *N_RRP_* of 434, 389, and 125 SVs, respectively (100:90:29), thus demonstrating a 3.5-fold and statistically significant reduction in Bafi30.

We also determined the quantal content *m* for eIPSC_1_ of the 100-Hz challenge period (*m_1_* = eIPSC_1_/*q; q* was determined from the preceding *Recov2* period). Again, the Ctrl_vs_Bafi30 comparison showed a statistically significant difference (47 vs. 21 SVs, i.e., ratio of 100:45; Figure 6D3). Finally, we observed a statistically significant difference for the release probability *P_v_* in the Ctrl_vs_Bafi30 comparison (*P_v_* = m_1_/I_RRP_). While *P_v_* was 11% in the Ctrl, it was significantly increased to 16% in Bafi30 (~1.5-fold; Figure 6D4).

The 3.5-fold reduction in *N_RRP_* was confirmed by means of a fluctuation analysis assuming a binomial release of SVs (not shown). We have recently used this analysis to evaluate statistical fluctuations of *m* at MNTB-LSO synapses (Vahdat et al., 2025) and found a *P_v_* value of 93%, which is considerably higher than the value found in the present study.

Collectively, the reduction in *q* and *m* values (down to 70 and 45%, respectively; in combination down to 32%) underlies the >3-fold variation in eIPSC_1_ values in the Ctrl_vs_Bafi30 comparison at the onset of the 100-Hz challenge (cf. Figure 3D2).

### “Half-marathon” experiments (4-min|100-Hz challenge; 5 μM Bafi) confirm staggering emergence of pharmacological V-ATPase inhibition

**Figure 7:**
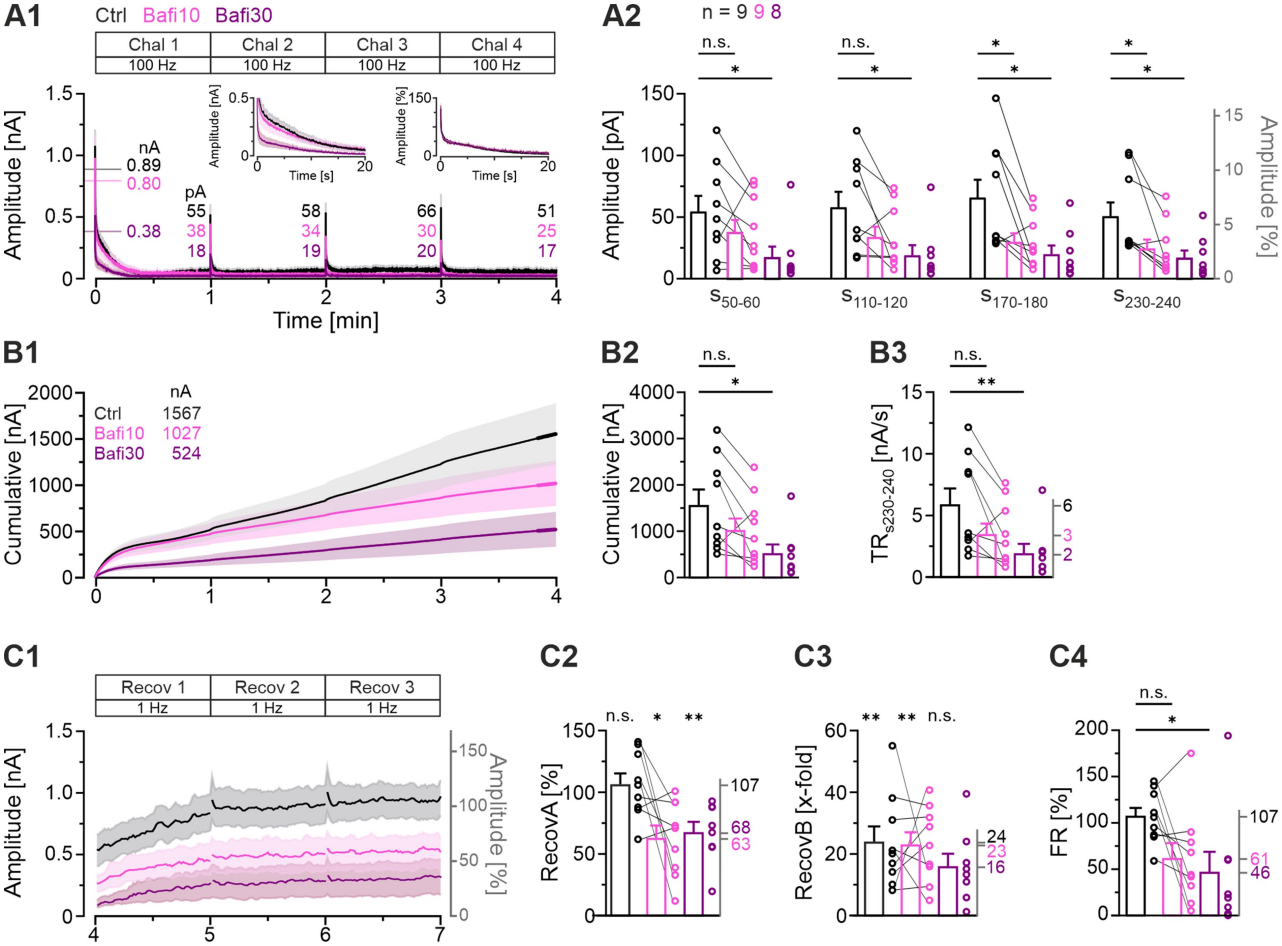
A higher Bafi concentration (5 μM) and harsher stimulation conditions further reduce synaptic strength. **(A1)** Time course of absolute eIPSC amplitudes (mean ± SEM) obtained during the 100-Hz|240-s challenge (“half-marathon”; Protocol 2; see Figure 1C) for Ctrl (black), Bafi10 (5 μM, magenta), and Bafi30 (5 μM, purple). The Ctrl and Bafi10 data are from a different neuron than the Bafi30 data. A 6-s pause was introduced after each challenge period for data storage. The BL mean of the Ctrl was set to 100%. Horizontal lines indicate the mean BL amplitude for each cohort (numbers are also given). Numbers indicate absolute mean amplitudes during s_50-60_. The left inset shows a close-up of the first 20 s; the right inset shows the same, but for normalized amplitudes. **(A2)** Statistics at four different time intervals (s_50-60_, s_110-120_, s_170-180_, s_230-240_). **(B1)** Time course of cumulative amplitudes (same x-axis as in **A1**). Thick lines mark the 10-s periods in which TR was quantified (s_230-240_). Numbers in the upper left indicate the cumulative current after 4 min. **(B2)** Statistics for cumulative amplitudes. **(B3)** Statistics for TR. Mean values on right y-axis. **(C1)** Time course of absolute and normalized eIPSC amplitudes during the 1-Hz|180-s recovery after the 4-min challenge. A 6-s pause was introduced after each 60-s period for data storage. **(C2–C4)** Statistics for RecovA **(C2)**, RecovB **(C3)** and FR **(C4)**. Mean percentages on the right y-axis. Time courses are simple moving averages over three (1 Hz) or nine (100 Hz) data points (SEM lightly shaded). Ctrl and Bafi10, *n* = 9; Bafi30, *n* = 8. See Supplementary Table S5 for details, including statistics.

The above results demonstrated diminished yet persistent activity at MNTB-LSO synapses even after prolonged treatment with 2 μM Bafi (≥30 min; Figures 2–5). We thus sought to determine whether a higher Bafi concentration (5 μM) and more intense stimulation conditions would result in a greater reduction in neurotransmission, or even its complete abolition. The underlying procedure, designated “Protocol 2” or “half-marathon” (Figure 1C) comprised a Ctrl episode (4-min|100-Hz challenge followed by 3-min|1-Hz recovery, totaling 24,180 stimulus pulses) which was followed by a Bafi10 episode (paired recordings) and a final Bafi30 episode (from a different neuron). The stimulus conditions for each Bafi episode were identical to those employed for the Ctrl episode. The results are summarized in Figure 7, in which panel A1 illustrates the time course of the eIPSC amplitudes for the three cohorts. From the third challenge period onwards, that is not before 12,000 – 18,000 stimulus pulses, a significant reduction in steady-state eIPSC amplitudes was observed in the Bafi10 cohort (Figure 7A2, s_170-180_). This finding differs from the results obtained with Protocol 1 (see Figure 3). By the conclusion of the 4-min challenge period (s_230-240_), the steady-state amplitudes had decreased to 51, 25, and 17 pA (Figures 7A1,A2). The ~2-fold lower performance in Bafi10 and the 3-fold lower performance in Bafi30, in both cases statistically proven, unambiguously demonstrate the presence of a drug effect.

A more detailed examination of the time course of eIPSCs revealed that the largest amplitude differences occurred during the initial 20 s between the Ctrl and Bafi10 cohorts on one hand and the Bafi30 cohort on the other (left inset in Figure 7A1). This suggests that Bafi30 may experience significantly accelerated synaptic fatigue, whereas Ctrl and Bafi10 can draw from an intact RRP. However, the kinetics of depression remain largely indistinguishable (right inset in Figure 7A1).

Analysis of the cumulative eIPSC amplitudes corroborated the observation that a higher drug concentration and a more severe stimulation condition yielded had a greater effect on synaptic transmission (Figure 7B1). The Ctrl cohort was observed to elicit 1,567 nA, whereas the Bafi10 and the Bafi30 cohorts achieved only 66 and 33%, respectively (1,027 and 524 nA). For the Ctrl_vs_Bafi30 comparison, a statistically significant difference was observed (Figure 7B2; Supplementary Table S5). The results indicate that the inhibition of V-ATPase considerably impacts SV (re)filling when three conditions are met: first, a highly concentrated solution of Bafi is administered; second, the application time is considerable; third, the stimulation conditions are harsh. The results for the TRs_230-240_ corroborate this conclusion (Figure 7B3; Ctrl: 6; Bafi10: 3; Bafi30: 2 nA/s; ratio 100:50:33).

Our subsequent investigation focused on the recovery behavior following the half-marathon challenge, with particular attention paid to s_230-240_ (Figures 7C1–C4). Regarding *RecovA*, the Ctrl cohort exhibited complete recovery, reaching 107% of BL. In contrast, the Bafi10 and Bafi30 cohorts showed incomplete recovery, reaching only 63 and 68% of the BL level, respectively, and these values were significantly different from the BL (Figure 7C2). The ability of each cohort to reach higher eIPSC amplitudes after exhibiting very low levels at the end of the 4-min|100-Hz challenge was evaluated by assessing *RecovB*. The Ctrl and Bafi10 cohorts exhibited significantly higher amplitudes (24- and 23-fold, respectively; Figure 7C3, see also panel A2, s_230-240_). In contrast, *RecovB* did not reach statistical significance for Bafi30 (16-fold). Finally, there was a significant difference in *FR* between the Ctrl and Bafi30 cohorts, but not between the Ctrl and the Bafi10 cohorts (Figure 7C4; Ctrl: 107%; Bafi10: 61%; Bafi30: 46%).

During the harsh 4-min|100-Hz challenge, we made an intriguing observation following the 6-s pauses required for data storage at minutes 1, 2, and 3, namely short-term recovery (Figure 7A1; NB: a similar observation was made by Qiu et al. (2015) at Calyx-of Held – MNTB synapses). As the stimulation was terminated during these three pauses, we were able to assess and quantify this short-term recovery without interference. To achieve this, we applied a variant of the *FR* formula ($\frac{\text{eIPS}C_{1}\;\text{of Chal}2-\text{mean amplitud}e_{s50-60}\text{of Chal}1}{\mathrm{BL}-\text{mean amplitud}e_{s50-60}\text{of Chal}1}$ etc.) and found lower values in the Bafi cohorts compared to Ctrl, but still ongoing recovery (Figure 7A1). The Ctrl: Bafi10: Bafi30 ratios were: min_1_: 100:95:89; min_2_: 100:71:64; min_3_: 100:59:48. This implies that the presence of Bafi did not abolish SV replenishment, even when a brief time domain of just a few seconds and the harsh half-marathon conditions are considered. The results confirm those obtained for the recovery periods lasting 60 s.

Taken together, the results from the half-marathon experiments show that a 10-min application time of Bafi at an extraordinarily high concentration (5 μM) is sufficient to elicit blocking effects on synaptic transmission. However, the effects do not become evident before at least 12,000 stimulus pulses are given within ~2 min. Moreover, they still enable eIPSC amplitudes of sufficient height (~50% of Ctrl) during sustained activation, resulting in statistically indistinguishable cumulative eIPSC amplitudes. Recovery from depression is impaired but still reaches about two third of the BL level, implying ongoing SV (re)filling. As expected, the Bafi30 effects are generally more severe.

### Reduction of synaptic strength is drug-specific and not time-dependent (sham control experiments)

One potential pitfall of the experiments discussed above is that the observed decrease in eIPSC amplitudes cannot be unambiguously attributed to a drug effect. Rather, the decrease may be time-dependent. To address this concern, we performed sham control experiments essentially identical to Protocol 1 (see Figure 3), except that only the solvent for Bafi was washed in (0.1% EtOH). We observed an almost perfect superposition of the current traces (Supplementary Figures S1A1–A3,B1,C1,D1,E1; Supplementary Table S6). In essence, these results impressively demonstrate that there was no time dependence. In both EtOH10 and EtOH30, the synaptic performance was statistically indistinguishable from Ctrl (in ACSF) for BL analysis (Supplementary Figures S1A4,A5), for challenge periods (Supplementary Figures S1B1,B2,C1,C2,D1,D2,E1,E2), and for robust recovery (Supplementary Figures S1F1–F3,G1–G3,H1–H3,I1–I3).

Taken together, the data show no effect of EtOH on synaptic transmission, suggesting that the impairment is solely due to the drug (Bafi) and not due to the solvent or the time.

We also compared EtOH effects with Bafi effects (Supplementary Figure S2; Supplementary Table S7; see also Figure 3) and found significantly impaired transmission in Bafi30_vs_EtOH30 (Supplementary Figures S2C1–C3) but neither in Ctrl_Bafi__vs_Ctrl_EtOH_ (Supplementary Figures S2A1–A3) nor in Bafi10_vs_EtOH10 (Supplementary Figures S2B1–B3). Impaired transmission was evident during the last 10 s at each challenge frequency (Supplementary Figure S2C2) and during each recovery period (Supplementary Figure S2C3). Collectively, the results from the sham control experiments confirm that the reduced synaptic strength observed in the presence of bafilomycin is caused by V-ATPase inhibition. Time-dependent or solvent-dependent effects can be excluded.

### Bafi effects are reproduced by folimycin

In a complementary set of experiments, we assessed the effects of inhibited V-ATPase activity by repeating the experiments with Protocol 1, except that Bafi was replaced by folimycin (Foli, 1 μM). The results are shown in Supplementary Figures S3, S4. Foli essentially recapitulated the effects of Bafi in direction and magnitude (Supplementary Tables S8, S9). For 86% (67/78) of the parameters that we quantified in both the Bafi and Foli cohorts, the statistics were congruent, thus demonstrating that both drugs affected V-ATPase activity in a very similar manner (Supplementary Table S9). For example, the BL was significantly reduced in Foli30 compared to the Ctrl (49%; 633 vs. 1,321 pA), but not in the Foli10 (87%; 1,151 pA; Supplementary Figures S3A4,A5). A consistent observation was made for the Bafi cohort, namely 38 and 89%, respectively (Supplementary Table S9; cf. Figures 3A4,A5). Furthermore, the Foli30 cohort showed increased synaptic depression compared to the Ctrl at each challenge frequency and in each time window (16 parameters), whereas the Foli10 cohort was statistically indistinguishable from the Ctrl for each of those 16 parameters (Supplementary Figures S3B1–B5,C1–C5,D1–D5,E1–E5). A similar scenario had been found for Bafi (Supplementary Table S9; cf. Figure 3). Finally, application of Foli did not result in significantly lower *q* values in Foli10, but it did so in Foli30 (Supplementary Figure S4E3), consistent with the previous findings on Bafi.

Treatment with Foli30 also resulted in impaired recovery after each challenge period (Supplementary Figures S4A1,A2,B1,B2,C1,C2,D1,D2; Supplementary Table S10). For Foli10, however, such an impairment was only detectable after a 200-Hz exposure (Supplementary Figures S4A2,B2,C2,D2). *RecovB* reached statistical significance in each case (Supplementary Figures S4A3,B3,C3,D3), suggesting that refilling mechanisms are still functional, albeit significantly reduced. *FR* values showed a significant difference in each comparison of Ctrl_vs_Foli30 (Supplementary Figures S4A4,B4,C4,D4).

The *I_RRP_* of Foli10 was statistically indistinguishable from the Ctrl, but it was drastically reduced in Ctrl_vs_Foli30 (~4-fold; Supplementary Figures S4E1,E2). The *N_RRP_* in the Ctrl, Foli10, and Foli30 contained 832, 620 and 374 SVs, respectively (Supplementary Figure S4E2). Thus, the *N_RRP_* differed only 2.2-fold between Ctrl and Foli30. The lower *N_RRP_* ratio compared to *I_RRP_* is explained by the finding that *q* values were almost 2-fold lower in Foli30 than in Ctrl (13 vs. 23 pA; Supplementary Figure S4E3). Notably, this reduction was greater than that shown for Bafi30 in Figure 6C3. The Foli30 cohort also showed a statistically significant reduction in *m*, similar to the Bafi30 results (64 SVs/38 SVs = 1.7-fold; Supplementary Figure S4E4). In contrast, *P_v_* values were not altered by Foli (10, 11, 9%; Supplementary Figure S4E5; different from Bafi30).

The similar results obtained with Bafi or Foli, when considered together, underscore the need for prolonged drug treatment to impair synaptic transmission. Statistically significant differences occurred in 39 out of 46 Ctrl_vs_Bafi30 comparisons and in 39 out of 39 Ctrl_vs_Foli30 comparisons, representing 85 and 97%, respectively (Supplementary Table S9). In contrast, with shorter drug treatment, only 3 out of 46 comparisons in Ctrl_vs_Bafi10 and 0 out of 39 in Ctrl_vs_Foli10 showed statistical significance (7 and 0%, respectively). These results impressively highlight the delayed onset of impaired transmission in the presence of V-ATPase blockers. In conclusion, the effects of Foli essentially recapitulated those of Bafi in direction and magnitude. They provide consistent evidence that V-ATPase activity is necessary to generate glycine-filled SVs and ensure robust synaptic transmission at MNTB-LSO synapses. However, SV (re)filling does not appear to depend on V-ATPase activity alone.

### The Na^+^/H^+^ exchanger NHE6 is highly abundant in presynaptic axon terminals around LSO neurons and in MNTB neurons

Because our Bafi and Foli analyses had revealed an only incomplete blockade of transmission, despite severe treatment conditions, we wondered whether there might be another proton pump besides the V-ATPase that could acidify the SVs, thereby creating a favorable proton gradient for VIAAT-mediated glycine import. One candidate is the Na^+^/H^+^ exchanger (NHE; see Figure 1C). Thirteen isoforms have been identified (Pedersen and Counillon, 2019), of which NHE6-NHE9 are located in the membranes of intracellular organelles (Alexander et al., 2017).

In order to assess the distribution of NHE proteins in the LSO and other superior olivary complex (SOC) nuclei, we used immunohistochemistry, thereby focusing on the NHE6 isoform. Our approach was motivated by mRNA sequencing results obtained in our group from laser-dissected tissues (Maraslioglu-Sperber, 2021). These results demonstrated high expression of *Slc9A6*, the gene encoding NHE6, in the LSO and MNTB of juvenile mice, among nine NHE isoforms analyzed. *Slc9A6* expression was the most pronounced, with a level of 11 transcripts per million in the LSO and 12 in the MNTB. Interestingly, NHE6 belongs to the subset of Na^+^/H^+^ exchangers that are found in recycling endosomes (Brett et al., 2002; Masereel et al., 2003; Orlowski and Grinstein, 2004; Nakamura et al., 2005) and secretory organelles (Miyazaki et al., 2001; Kondapalli et al., 2014). Consistent with this, NHE6 has been identified in SVs (Preobraschenski et al., 2014; Taoufiq et al., 2020). Finally, NHE6 has been implicated in luminal pH regulation (Ohgaki et al., 2010; Ohgaki et al., 2011). Taken together, these findings identified NHE6 as an interesting candidate for our MNTB-LSO study.

**Figure 8:**
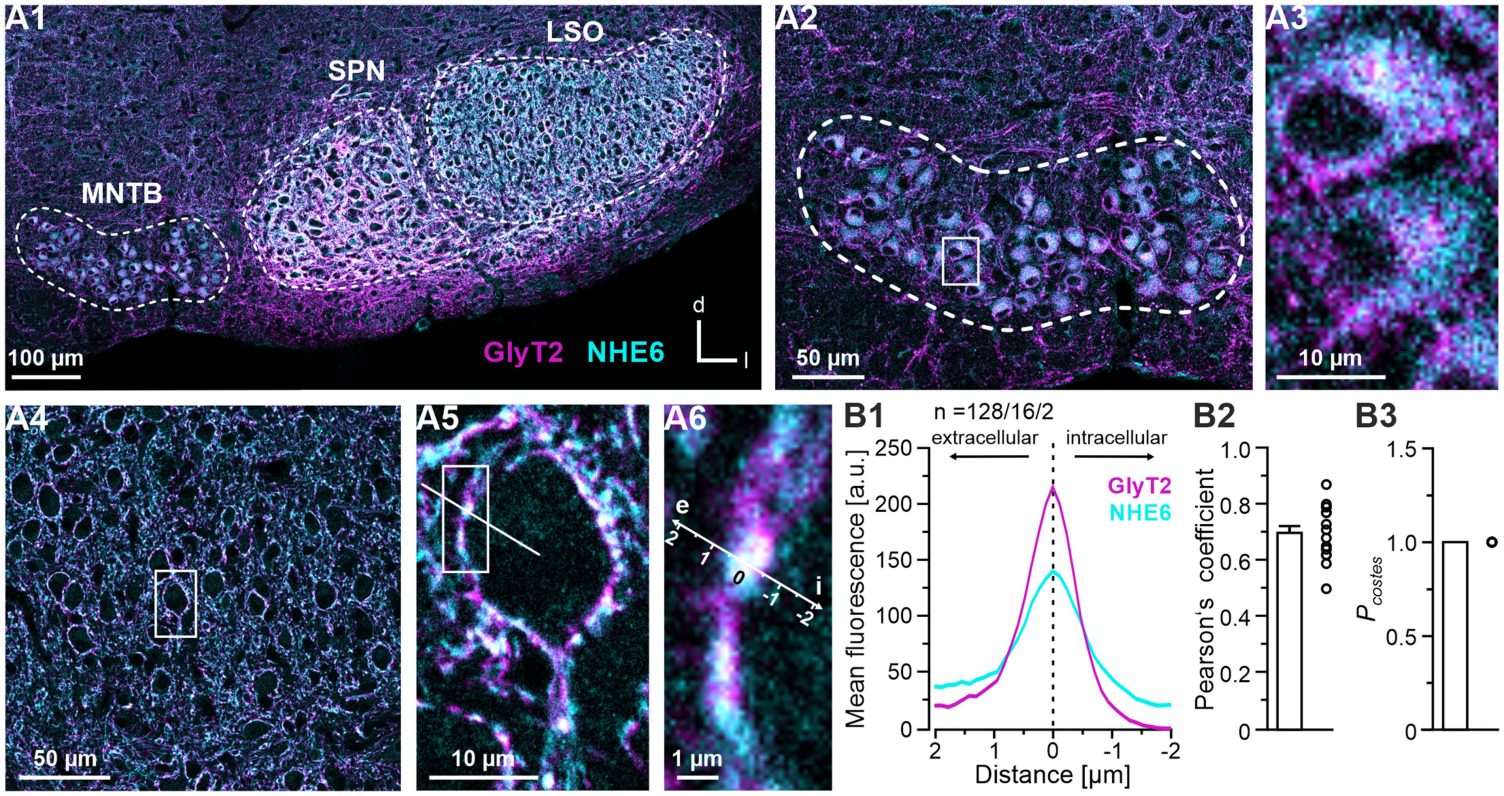
Co-distribution of NHE6 and GlyT2 in the superior olivary complex (SOC). **(A)** Immunohistochemical analysis of NHE6 and GlyT2 in a coronal brainstem section at P11. **(A1)** Double-labeling for NHE6 (cyan) and GlyT2 (magenta) in the SOC. MNTB, SPN and LSO are outlined by dotted lines. **(A2)** NHE6 and GlyT2 immunosignals in the MNTB. **(A3)** Close-up of two representative MNTB neurons from the framed area in **(A2)**. **(A4)** Double-labeling in the LSO. **(A5)** Close-up of a representative LSO neuron in the framed area in **(A4)**. A representative line scan is shown. **(A6)** Close-up of the framed area in **(A5)**. The spatial axis is also used in **(B1)**. **(B)** Quantification of NHE6 and GlyT2 co-distribution. **(B1)** Averaged intensity profiles for NHE6 (magenta) and GlyT2 (cyan). Prior to averaging, each profile was aligned to the position of the maximum GlyT2 signal. See Materials and Methods for details. Negative and positive μm values indicate intracellular and extracellular signals, respectively. *n*, number of profiles/cells/animals. **(B2)** Pearson’s coefficients and **(B3)** corresponding *P_Costes_* values. The Pearson’s coefficient was 0.70 ± 0.02 (16 cells) and the *P_Costes_* value was 1.00 ± 0.00 (16 cells; NB: co-distribution is indicated by *P_Costes_* > 0.95). Optical thickness: 1 μm. d, dorsal; e, extracellular; i, intracellular; l, lateral; LSO, lateral superior olive; MNTB, medial nucleus of the trapezoid body; SPN, superior paraolivary nucleus.

To assign NHE6 signals to glycinergic neurons, our immunohistochemical analysis included a double-labeling strategy for NHE6 and GlyT2, which is a marker protein for glycinergic neurons (Friauf et al., 1999; Eulenburg et al., 2005). We found strong NHE6 immunofluorescence in the MNTB, the SPN and the LSO (Figure 8A1). At the cellular level, the cytoplasm of virtually every glycinergic MNTB soma was NHE6 positive (Figures 8A2,A3). This pattern stood in contrast to that observed in the LSO, where neuronal somata exhibited almost complete absence of NHE immunoreactivity, yet were decorated with double-labeled puncta in a perisomatic fashion (Figures 8A4–A6). We attribute these puncta to axon terminals of MNTB neurons. Quantification of NHE6 and GlyT2 co-localization confirmed the close proximity of the immunosignals (Figures 8B1–B3). Taken together, these results suggest that terminals of MNTB axons that contact LSO somata contain substantial amounts of NHE6.

### Application of EIPA to brainstem slices with the aim to block Na^+^/H^+^ exchangers results in severely impaired transmission, yet recovery still occurs

No selective antagonist for NHE6 has been identified to our knowledge. Nevertheless, NHE-mediated Na^+^/H^+^ exchange in general is reportedly suppressed by the “broadband” antagonist ethyl-isopropyl-amiloride (EIPA; Vigne et al., 1983; Masereel et al., 2003; Hori and Takamori, 2021). We therefore characterized transmission at MNTB-LSO synapses in the presence of EIPA (100 μM). The procedure is illustrated in Figure 1C and outlined in Protocol 3. It consists of a Ctrl episode performed in ACSF and comprised a 1-min|50-Hz challenge, a 1-min|100-Hz challenge and two 1-min|1-Hz recovery periods, corresponding to 9,120 stimulus pulses in total. Subsequently, an EIPA10 episode was initiated that was identical in structure to the preceding Ctrl episode (paired recordings).

**Figure 9:**
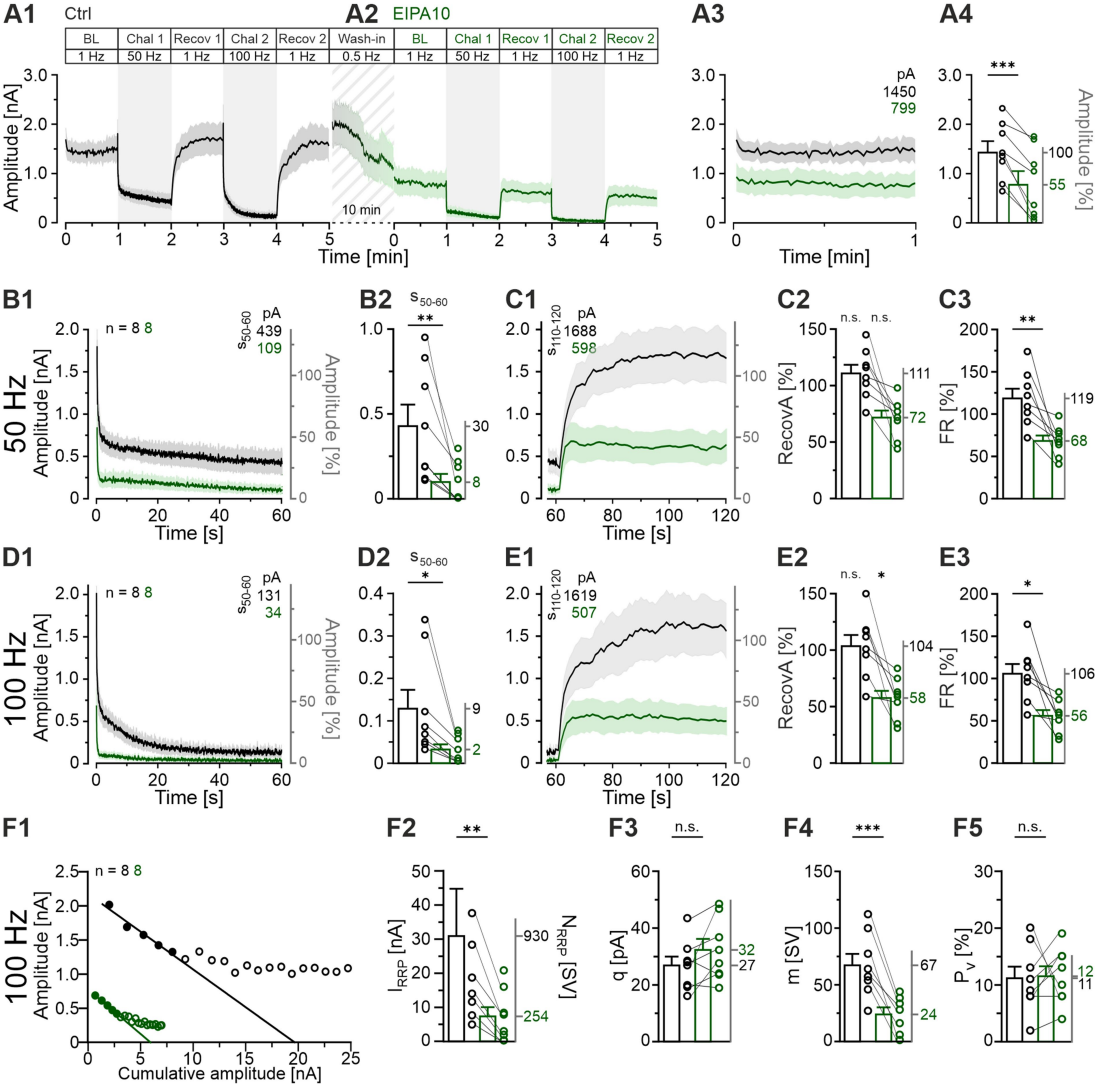
Application of EIPA with the intention of blocking NHE activity results in a markedly reduced sustained transmission but does not prevent recovery. **(A1,A2)** Time course of eIPSC amplitudes (mean ± SEM) for Ctrl (black) and EIPA10 (100 μM, green), obtained from paired recordings (Protocol 3, see Figure 1C). Diagonally striped region represents the wash-in period for EIPA. **(A3)** Close-up of the BL traces. The numbers in the upper right indicate the absolute mean amplitudes. **(A4)** Statistical analysis for the BL periods (mean Ctrl = 100%, EIPA10: 55%). **(B1)** Time course of absolute and normalized eIPSC peak amplitudes during the 50-Hz|60-s challenge. The numbers in the upper right indicate the absolute mean amplitudes during s_50-60_. **(B2)** Statistical analysis during s_50-60_. **(C1)** Time course of absolute and normalized eIPSC peak amplitudes during a 1-Hz|60-s recovery period following the 50-Hz challenge. The numbers in the upper left indicate the mean amplitudes during s_110-120_. **(C2,C3)** Statistical analysis for RecovA **(C2)** and FR **(C3)**. Mean percentages on the right y-axis. **(D1–E3)** Same as **(B1–E3)**, but for 100 Hz. **(F)** Analysis of synaptic parameters. **(F1)** Elmqvist and Quastel plots were used to determine *I_RRP_;* forward extrapolation was applied to the linear fit through eIPSCs_1-5_ evoked at 100 Hz. Cohort values (see Materials and Methods for details). **(F2)** Statistical analysis for *I_RRP._* Mean values of *N_RRP_* on the right y-axis. **(F3–F5)** Statistics for *q* (obtained from a 300 s period over BL and Recov1-Recov4). **(F3)**, *m* **(F4)**, and *P_v_* **(F5)**. Means on the right y-axis. Variables on the left y-axis in **(B2–D2)** are the same as in **(B1)**. Note the different amplitude scaling in **(B2,D2)**. Time courses are simple moving averages over three (1 Hz) or nine (50-100 Hz) data points (SEM lightly shaded). Ctrl, *n* = 8; EIPA10, *n* = 8. See Supplementary Table S11 for details, including statistics.

In the Ctrl, the synaptic depression during each challenge period, along with the subsequent recovery, occurred in a manner consistent with our expectations (Figure 9A1). However, we made an unanticipated observation during the 10-min wash-in period of EIPA, during which MNTB axons were stimulated at a low frequency of 0.5 Hz. The eIPSC amplitudes decreased from 1,619 pA at the end Ctrl-Recov2 to 799 pA at the beginning of EIPA-BL (Figures 9A1,A2). This resulted in a markedly reduced BL of 55% (Figures 9A3,A4), suggesting an initial inhibitory effect of EIPA on transmission that cannot be attributed to challenge-induced SV depletion. During each challenge period, the transmission in EIPA10 exhibited a ~ 4-fold reduction compared to the Ctrl (Figures 9B1,B2,D1,D2; Supplementary Table S11). *RecovA* showed no statistically significant difference from the preceding BL in each cohort following 50-Hz challenge (Figures 9C1,C2), indicating that the efficacy of recovery remained unchanged. However, after 100-Hz challenge, *RecovA* was insufficient (Figures 9E1,E2). Furthermore, the *FR* was significantly lower than that for the Ctrl after each challenge period (Figures 9C3,E3; 50 Hz: 119 vs. 68%, 100 Hz: 106 vs. 56%), suggesting that EIPA may indeed inhibit SV (re)filling. The observation that the synaptic strength exceeded 500 pA during each recovery period, however, indicates that SV (re)filling is not completely abolished by the drug (Figures 9C1,E1).

As before, we determined the readily releasable pool (*I_RRP_*) at the begin of the 100-Hz challenge period (Figure 9F1). We found a severe reduction in the presence of EIPA10 (~4-fold, from 30.9 to 7.3 nA; Figure 9F2). These *I_RRP_* values relate to *N_RRP_* values of 930 and 254 SVs, respectively. The quantal size *q* remained unaltered (Figure 9F3), while the quantal content *m* was almost 3-fold lower (Figure 9F4), in line with the diminished *N_RRP_*. Finally, the release probability *Pv* was found to be unaltered (Figure 9F5). In summary, the EIPA10 experiments yielded some surprising results, including the extraordinary time course of eIPSC amplitudes during the drug wash-in period (Figure 9A2), which ultimately resulted in an almost 2-fold lower BL compared to the Ctrl (Figure 9A3). Therefore, a word of caution is warranted regarding the interpretation of the EIPA10 results. This issue will be further discussed in the following section.

### EIPA has adverse effects on AP generation that require careful interpretation of the results on synaptic effects

**Figure 10:**
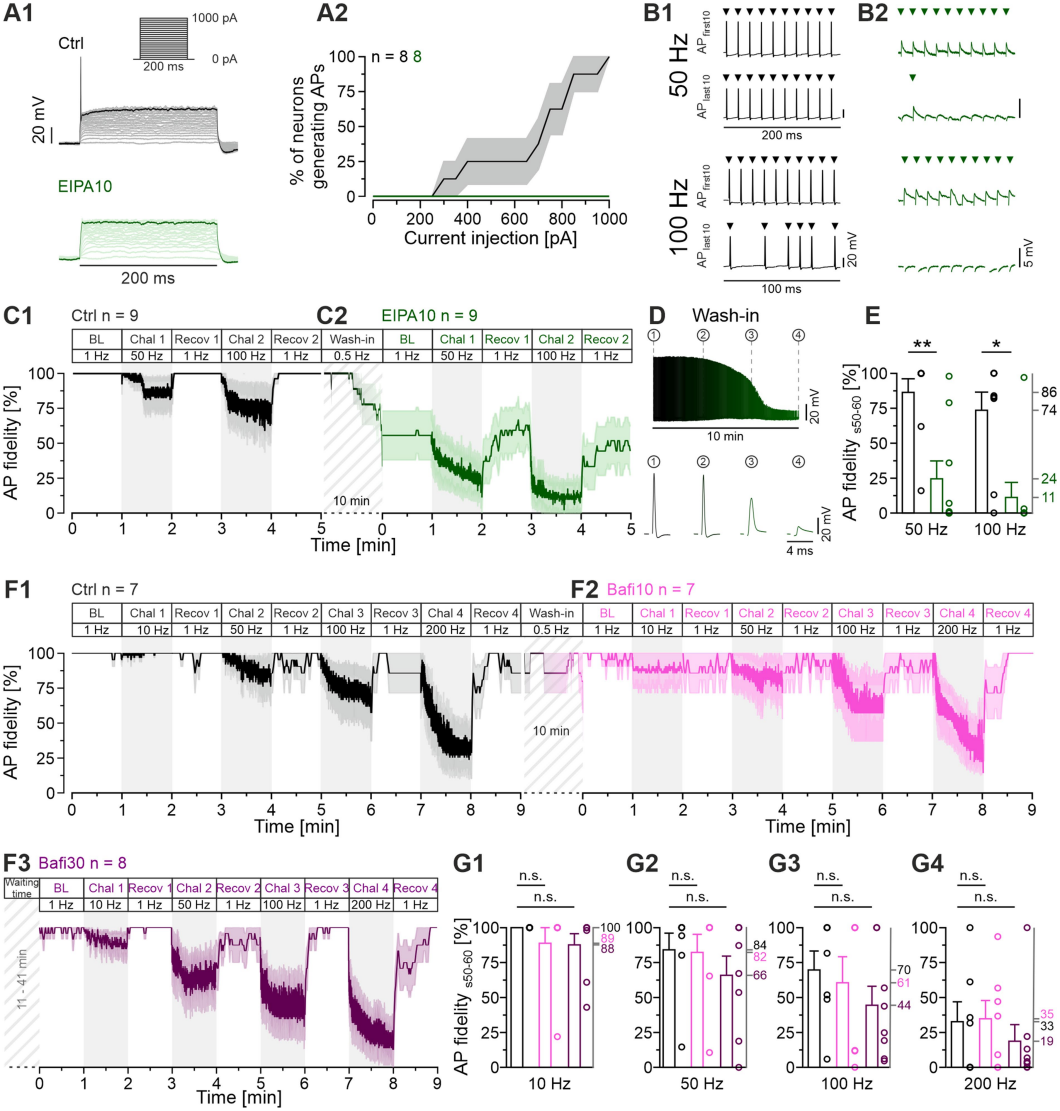
Application of EIPA reduces the reliability of AP firing, whereas Bafi does not. **(A1)** Representative voltage traces from a Ctrl MNTB neuron (black, top) and after application of EIPA10 (100 μM; green, bottom) at rheobase 850 pA. The traces show voltage responses to rectangular current pulses (inset: 0 to 1,000 pA; 50 pA increments; 200 ms duration). **(A2)** Percentage of neurons generating APs (mean ± SEM) as a function of the current amplitude. Note the absence of APs upon EIPA treatment. Same neurons as shown in Figure 9. **(B)** Representative antidromic APs during Chal 1 and Chal 2 periods, showing the first and last 10 APs for Ctrl **(B1)** and EIPA10 **(B2)**. Each arrow marks an AP. Note the different amplitude scaling in **(B1,B2)**. **(C1,C2)** Time course of AP fidelity (mean ± SEM) for Ctrl **(C1)** and EIPA10 **(C2)** obtained with protocol 3 (see Figure 1C, antidromic stimulation). Diagonally striped regions represent the wash-in period of EIPA. A 6-s long pause was introduced after the BL and each 60-s recovery period. Ctrl, *n* = 9; EIPA10, *n* = 9. **(D)** AP amplitudes during the 10-min wash-in period (top), and magnification of four representative APs at different time points (bottom). **(E)** Statistical analysis for AP fidelity during s_50-60_. **(F)** Time course of AP fidelity (mean ± SEM) for Ctrl (black) **(F1)** and Bafi10 (magenta) **(F2)** obtained from paired recordings with protocol 1 (see Figure 1C, antidromic stimulation). **(F3)** Time course for Bafi30 (purple). Data in **(F3)** are from a different neuron than in **(F1,F2)**. Diagonally striped regions indicate perfusion periods of 2 μM Bafi. A 6-s pause was introduced after the BL and each 60-s recovery period. **(G)** Statistics for AP fidelity during s_50-60_ for 10 Hz **(G1)**, 50 Hz **(G2)**, 100 Hz **(G3)**, and 200 Hz **(G4)**. Variables on the left y-axis in **(G2–G4)** are the same as in **(G1)**. Mean percentages on the right y-axis. Ctrl, *n* = 7; Bafi10, *n* = 7; Bafi30, *n* = 8. See Supplementary Table S12 for details, including statistics.

While performing the EIPA experiments, we noticed that the drug affected the AP behavior of LSO neurons. In the absence of EIPA, Ctrl neurons fired an onset AP in response to a 200-ms depolarizing current pulse, which is typical for LSO principal neurons (Sterenborg et al., 2010; Friauf et al., 2019). This onset AP was not elicited in the presence of EIPA (Figure 10A1), where each of the eight neurons analyzed exhibited a complete cessation in firing (Figure 10A2). This unanticipated outcome prompted us to be concerned about the activation efficiency of the MNTB neurons in the synaptic stimulation experiments. To address this concern, we performed antidromic stimulation experiments on MNTB neurons as previously described (Müller et al., 2022). The stimulation regime was according to Protocol 3, and APs with peak amplitudes <1 mV were considered as failures. The Ctrl MNTB neurons showed mild failure behavior at the end of the 50-Hz and the 100-Hz challenge period, but the amplitudes of their APs remained largely unaltered (example traces in Figure 10B1 and the 60-s time courses for the cohort in Figure 10C1). In contrast, EIPA10 MNTB neurons exhibited a high failure rate and a drastic reduction in AP peak amplitude, resulting in the emergence of mini spikes (spikelets) whose amplitude was only ~3 mV (Figure 10B2; time course in panels C2 and D). The AP fidelity of the Ctrl during s_0-10_ was 99 and 89% at 50-Hz and 100-Hz challenge, respectively (Figure 10C1). The corresponding values for EIPA10 were only 45 and 20% (Figure 10C2). By s_50-60_, the fidelity values were 86 and 74% for the Ctrls and 24 and 11% for EIPA10 (Figure 10E). The Ctrls demonstrated the ability to regain perfect fidelity values of 100% during each recovery period. In contrast, the corresponding values for EIPA10 were only 60 and 49% (Figure 10C2). As mentioned above, the AP fidelity was only 45% during s_0-10_ of the 50-Hz challenge (Figures 10B2,C2). It is noteworthy that a similarly low level (56%) was already present during the BL period, and it emerged during the 2nd half of the 10-min wash-in period of EIPA at 0.5-Hz stimulation (Figure 10C2). In sum, the results illustrate considerable and deleterious effects of EIPA10 on AP generation and/or AP conduction.

In a next step, we conducted antidromic stimulation experiments in the presence of the V-ATPase inhibitor Bafi (2 μM) to ascertain whether the drug might also exert any deleterious effects on AP generation, as we had demonstrated for EIPA. Protocol 1 was employed to emulate the synaptic stimulation scenario previously utilized for Bafi (cf. Figures 1, 2). Detailed results are presented in Figures 10F1–F3,G1–G4 and in Supplementary Table S12. For the Ctrl, the s_50-60_ fidelity values were 100, 84, 70 and 33%, respectively, at challenge frequencies of 10, 50, 100 and 200 Hz. The corresponding values for Bafi10 were 89, 82, 61 and 35% (Figures 10G1–G4). None of these values was statistically different from the Ctrl. Finally, the Bafi30 values were 88, 66, 44 and 19%, respectively, and again, no value was statistically different from the Ctrl. Furthermore, the recovery from depression was robust in each case, ranging from 86 to 100% (Figure 10F3). Collectively, these findings imply that Bafi elicits no considerable aversive side effects on AP behavior, in clear contrast to EIPA.

### When corrected for AP failures, the blocking effect of Bafi on SV replenishment is confirmed. In contrast, EIPA does not appear to impair replenishment

**Figure 11:**
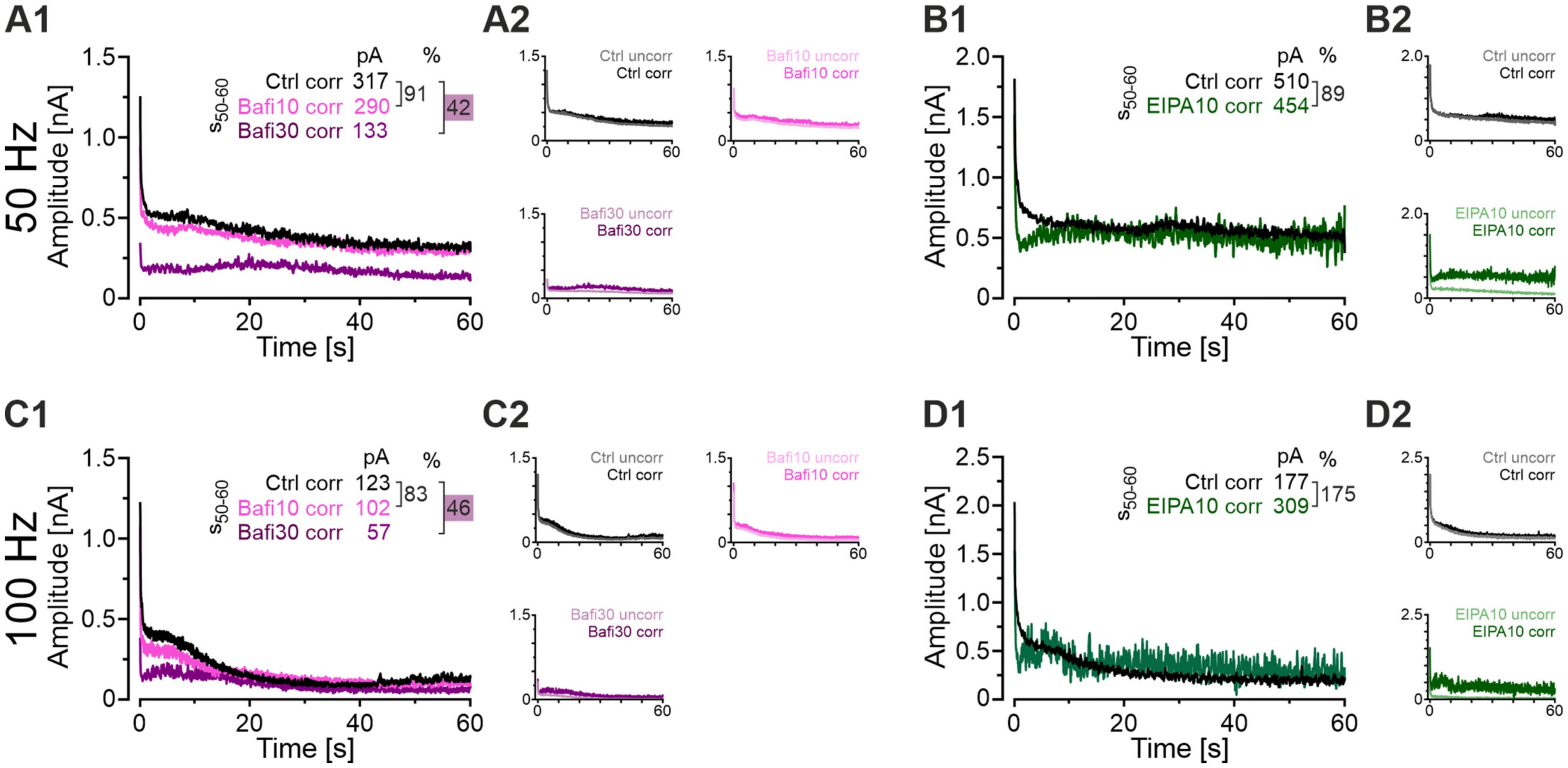
When AP failures are corrected, EIPA no longer affects synaptic transmission. **(A1)** Time course of eIPSC mean peak amplitudes upon AP correction for Ctrl (black), Bafi10 (magenta), and Bafi30 (purple) during 50-Hz|60-s challenge. Upper right numbers indicate the mean corrected amplitudes during s_50-60_ and the percentages (e.g., $\frac{133}{317}\;$= 42%). Ratios < 50% are highlighted in purple. **(A2)** Time course of corrected amplitudes (dark color) and uncorrected amplitudes (light color) for Ctrl (upper left), Bafi10 (upper right), and Bafi30 (lower left). **(B1)** Same as **(A1)**, but for EIPA10 (green). **(B2)** Same as **(A2)**, but for Ctrl (top) and EIPA (bottom). **(C1–D2)** Same as **(A1–B2)**, but for 100 Hz. Variables on the left y-axis in **(A2,B2,C2,D2)** are the same as in **(A1)**. Note the different amplitude scaling in **(B1,B2,D1,D2)**. See Supplementary Table S13 for details.

To consider the influence of AP failures on the overall transmission efficiency of MNTB-LSO synapses, we performed a mathematical analysis. This analysis aimed to separate effects on AP generation and/or AP conduction from those caused by drug-induced synaptic fatigue. For this purpose, mean eIPSC values from the challenge periods were corrected by assuming 100% AP fidelity. This allows to conservatively attribute the changes in eIPSC amplitude to changes in the presynaptic release machinery. The following example illustrates the procedure: If the AP fidelity rate was 40% at a given time point and the corresponding eIPSC amplitude was 210 pA, the latter value was multiplied by 2.5, thereby obtaining a “failure-corrected” value of 525 pA. The procedure was employed to correct the EIPA10, Bafi10 and Bafi30 means presented in Figures 3, 9, respectively. The results from 50- and 100-Hz challenge are shown in Figure 11 and Supplementary Table S13 provides a detailed quantification of the findings for the s_50-60_ periods. Two arbitrary thresholds were set (50 and 200%), and they revealed no effect on synaptic transmission in the Bafi10 experiments upon failure corrections, thus confirming the aforementioned results (Figures 11A1,A2,C1,C2). In contrast, synaptic transmission was affected in Bafi30 upon correction, as described before. However, there was no complete abolishment, because eIPSC amplitudes were only moderately decreased, as documented by $\frac{\text{drug}}{\text{Ctrl}}$ ratios in the range of 42-46%. For the EIPA experiments, our failure-correction revealed that this drug appears to leave SV (re-)filling unaffected (Figures 11B1,B2,D1,D2). An unchanged *q* value upon EIPA treatment (Figure 9F3) provides further evidence that EIPA does not appear to affect SV (re)filling.

### Summary of the major results

The main findings of the present study are as follows: (1) In the absence of any V-ATPase blocker, synaptic transmission at inhibitory MNTB-LSO synapses is robust and remarkably resistant to fatigue, even under severe high-frequency stimulation conditions in the minute range and beyond (the half-marathon experiments included 24,000 stimulus pulses in 4 min; Figure 7). Furthermore, recovery from synaptic depression is rapid and complete, demonstrating great resilience. (2) Interfering with SV refilling by pharmacologically blocking V-ATPase activity with Bafi or Foli reduces steady-state eIPSCs only after prolonged drug application (≥30 min; Figures 3, 4; Supplementary Figures S3, S4). (3) However, the amplitude reduction is only moderate, so that transmission remains at a level of ~30%. (4) In line with the amplitude reduction, the quantal size *q* is reduced, but only to ~70% and only after prolonged blocker use (Figure 6; Supplementary Figure S4E3). (5) The ability to recover from synaptic depression is maintained in the presence of any V-ATPase blocker (Figure 5; Supplementary Figure S4). (6) Even when the drug concentration is increased and the stimulus conditions are most severe (half-marathon), Bafi10 effects are only evident after > 12,000 stimulus pulses (Figure 7). This further demonstrates the resistance of MNTB-LSO synapses to pharmacological treatment. (7) Application of EIPA to block NHE activity greatly reduces AP fidelity and thus affects neurotransmission (Figure 9). However, after correction of the AP failures, the hypothesis that EIPA affects SV refilling cannot be maintained (Figures 10, 11). Collectively, these major results suggest that effective SV refilling at MNTB-LSO synapses, especially under harsh stimulus conditions, does not depend solely on V-ATPase activity. Rather, another mechanism likely coexists that provides an H^+^ source for VIAAT and thus helps to generate SVs with a sufficiently high glycine content.

## Discussion

In the present study, we investigated the robustness of sustained synaptic transmission at inhibitory glycinergic synapses in the auditory brainstem, thereby emphasizing the role of SV refilling. To this end, we applied the drugs Bafi, Foli and EIPA to acute mouse brainstem slices and performed whole-cell patch-clamp experiments with long-lasting high-frequency stimulation (minute range, up to 200 Hz). The major results are summarized at the end of the Results section. They demonstrate an important role for the V-ATPase blockers Bafi and Foli, thereby confirming the importance of this pump in generating the H^+^ gradient that VIAAT utilizes to (re-)fill SVs. However, despite rigorous treatment and stimulation conditions, the blocking effects were only moderate, an unexpected outcome. The incomplete block suggests that proper V-ATPase activity at MNTB-LSO inputs is necessary but not sufficient for maintaining transmission. When examining the effects of EIPA on NHE(6) activity, we observed adverse side effects, namely profound blockade of APs. This indicates that EIPA is unsuitable for specifically targeting vacuolar NHE transport activity. These findings prompt further investigation into the potential contribution of other H^+^ transporters, beyond the V-ATPase, in supplying H^+^ ions to fuel VIAAT and thereby ensuring adequate loading of SVs with neurotransmitter molecules.

### Incomplete blocking of V-ATPase is unlikely to explain the moderate impairment at MNTB-LSO synapses

We argue that an incomplete pharmacological block of V-ATPase activity does not satisfactorily explain the persistence of transmission at MNTB-LSO synapses. Persistence was evident from substantial steady-state amplitudes, preserved quantal size, and recovery from depression. Notably, Bafi had to be applied for >30 min before its effect reached statistical significance (Figures 3–6; Protocol 1). Furthermore, relatively high concentrations of Bafi were required to elicit an effect (2 μM in Protocol 1; 5 μM in Protocol 2). Similar results were obtained with Foli (1 μM in Protocol 1; Supplementary Figure S3). The available literature supports this view. *In vitro* studies on dissociated cells, including primary neuron cultures, have used Bafi concentrations ranging from 0.25–4 μM (Granseth and Lagnado, 2008; Li et al., 2011; Peng et al., 2012; Ratnayaka et al., 2012; Santos et al., 2013; Siksou et al., 2013; Vargas et al., 2014; Di Giovanni and Sheng, 2015; Tang et al., 2015; Tagliatti et al., 2020). Comparable concentrations were used in organotypic culture studies (0.2–2 μM; Rose et al., 2013; Allain et al., 2016), purified synaptosomes (1 μM; Geerlings et al., 2001), acute brain slices (1–6 μM; Zhou et al., 2000; Harrison and Jahr, 2003; Satake et al., 2012; Muller et al., 2013; Song et al., 2013), an *ex vivo* optic nerve preparation (4 μM; Micu et al., 2016), and whole-larvae Zebra fish neuroblasts (1 μM; Einhorn et al., 2012). These concentrations are ~1,000 times higher than the K_i_ values for Bafi, which lie in the (sub-)nanomolar range (reviewed in Dröse and Altendorf, 1997). In most of the 20 cited studies, pretreatment with Bafi lasted only 30 s or 1–10 min (when specified). The five acute brain slice studies reported longer incubation times of 1 to ≥2.5 h. Thus, our drug application protocols appear reasonably consistent with established experimental parameters. Further support for complete V-ATPase block by Bafi comes from preliminary results demonstrating a gradual decline of eIPSC amplitudes when MNTB-LSO synapses are continuously stimulated at 1 Hz for ~40 min. These results predict an eventual abolishment of transmission and complete exhaustion of the releasable SV pool after ~51 min (see Supplementary Information).

### How do our results compare with those from other synaptic systems?

A comparison of our drug-related results with those obtained in other synaptic systems reveals both similarities and striking differences (Table 1). Regarding the effects of V-ATPase inhibition on spontaneously released SVs, only one study reported a complete loss of events (Song et al., 2013), whereas most found moderately reduced *q* values. This suggests that across synaptic systems, transmitter-filled SVs remain available when V-ATPase activity is inhibited. Notably, drug-treated MNTB-LSO synapses displayed much smaller effects on sPSC frequency than other synapse types.

**Table 1:** Synaptic transmission upon pharmacological blockade of the H^+^ pump V-ATPase with Bafi or Foli.

Synaptic system	Drug conditions	Analysis of spontaneous events		Analysis of evoked responses					Reference
		q (mPSC amplitude)	sPSC frequency	Stimulus conditions	Steady-state ePSC amplitude	TR (transmission rate)	FR (fractional recovery)	N_RRP_ (readily releasable pool)	
**MNTB – LSO** acute slices	2 μM Bafi, > 30 min	**100:77** Ctrl 26 pA, Drug 20 pA	**100:60** Ctrl 5.7 s^−1^, Drug 3.4 s^−1^	50 Hz | 60 s	**100:25** Ctrl 267 pA, Drug 88 pA	**100:35** Ctrl 17 nA/s, Drug 6 nA/s	**100:87** Ctrl 111%, Drug 66%	**100:29** Ctrl 434 SV, Drug 125 SV	Current study
**MNTB – LSO** acute slices	1 μM Foli, > 30 min	**100:54** Ctrl 24 pA, Drug 13 pA	**100:85** Ctrl 5.9 s^−1^, Drug 5.0 s^−1^	50 Hz | 60 s	**100:26** Ctrl 504 pA, Drug 126 pA	**100:29** Ctrl 29 nA/s, Drug 8 nA/s	**100:39** Ctrl 103%, Drug 40%	**100:45** Ctrl 832 SV, Drug 374 SV	Current study
**Hippocampal neurons** acute slices	4 μM Bafi, ≥ 2.5 h	**100:0** Ctrl 3 pA, Drug 0 pA	**100:0** Ctrl 1 s^−1^, Drug 0 s^−1^						Song et al. (2013)
**Cerebellar neurons** acute slices	1 μM Bafi, > 1 h	**100:55** Ctrl 55 pA, Drug 30 pA							Satake et al. (2012)
**Cerebellar Purkinje cells** acute slices	2 μM Bafi, ≥ 1 h		**100:9** Ctrl 4.9 s^−1^, Drug 0.43 s^−1^						Harrison and Jahr (2003)
**Calyx of Held – MNTB** acute slices	2 μM Foli, > 2.5 h			20 Hz | 400 s	**100:10** Ctrl 971 pA, Drug 99 pA	Ctrl 860 SVs/s Drug not available		Ctrl 2,196 SVs Drug not available	Qiu et al. (2015)
**Hippocampal neurons** primary cell culture	67 nM Foli, 15-20 min 2 min in 46 mM K^+^	**100:92** Ctrl 26 pA, Drug 24 pA	**100:10** Ctrl 7.8 s^−1^, Drug 0.8 s^−1^						Groemer and Klingauf (2007)
**Hippocampal neurons** acute slices	1 μM Bafi, > 2 h	**100:80** Ctrl 13.7 pA, Drug 11.0 pA	**100:6** Ctrl 1.7 s^−1^, Drug 0.1 s^−1^						Zhou et al. (2000)
**Hippocampal neurons** autapses	1 μM Bafi, 1 h	**100:59** Ctrl 21.0 pA, Drug 12.3 pA	**100:11** Ctrl 12.7 s^−1^, Drug 1.4 s^−1^						
**Hippocampal neurons** dissociated cultures	67 nM Foli, 10 min	**100:80** Ctrl 13 pA, Drug 10 pA	**100:17** Ctrl 6 s^−1^, Drug 1 s^−1^	low frequency no details	**100:75** Ctrl 1.6 nA, Drug 1.2 nA				Sara et al. (2005)

The table confronts the results of the present study to those of seven previous reports. All parameters (*n* = 7) were assessed via electrophysiological recordings. Notice the paucity of evoked response analyses in the literature. For simplicity reasons, data presentation for MNTB-LSO synapses is restricted to 50 Hz | 60 s and to long drug treatment (> 30 min). Ratios (in bold, Ctrl = 100%) are given to facilitate direct comparisons.

Far fewer studies have investigated evoked transmission during sustained high-frequency stimulation. At calyx of Held-MNTB synapses, V-ATPase inhibition did not completely abolish responses but reduced them to 10% of control during intense stimulation at 20 Hz for 400 s (Qiu et al., 2015). Thus, it appears that these auditory synapses also exhibit partial resistance to V-ATPase blockade, though less so than MNTB-LSO synapses, where steady-state transmission remained at ~25%. Taken together, we conclude that SV replenishment relies on both common and system-specific mechanisms. The persistence of transmission at both auditory synapse types, even under strong stimulation, points to an additional mechanism that ensures reliable SV (re-)filling at these high-fidelity synapses.

### MNTB-LSO synapses are unlikely to use a large reserve pool to efficiently replenish the RRP

During Protocol 1, we delivered 21,600 stimulus pulses. In the absence of Bafi, MNTB-LSO synapses reached a cumulative eIPSC amplitude of 2,842 nA (Figure 4E2). Combined with a mean *q* value of 26 pA (Figure 6C3), this corresponds to ~109,000 released SVs. Given that N_RRP_ comprised 434 SVs (Figure 6D2), the RRP was turned over ~250 times. Analysis of the Foli experiments yielded similar results, with ~190,000 released SVs and ~230 RRP turnovers. Sustaining neurotransmission at such levels requires highly efficient replenishment mechanisms, with candidate resources being the recycling pool and the reserve pool. The recycling pool is generally thought to contain ~5–20% of all SVs, whereas the reserve pool forms the largest reservoir (~80–90%; Rizzoli and Betz, 2005). The RRP typically accounts for only ~1–2%. For MNTB-LSO synapses, specific pool sizes have not been determined. However, estimates from calyx of Held-MNTB synapses suggest ~180,000 SVs in the reserve pool, ~7,000 in the recycling pool, and ~3,000 in the RRP (95, 3.5, and 1.5% of the total pool, respectively; Rizzoli and Betz, 2005). If a similar distribution applies to MNTB-LSO synapses, the total SV pool would contain only ~29,000 SVs. Consequently, the release of 109,000 or more SVs cannot be explained by consumption of a pre-existing reserve. Instead, replenishment must exceed the size of the total pool nearly 4-fold during the four minutes. In this context, it is noteworthy that the entire recycling pool of hippocampal synapses is exocytosed within ~20 s during 10-Hz stimulation (200 stimulus pulses; Fernández-Alfonso and Ryan, 2004), and ~40% of the total SV pool participates in recycling (Fernández-Alfonso and Ryan, 2006).

In our Bafi and Foli experiments, we aimed to block V-ATPase to prevent the reacidification of SVs in the recycling pool. In this scenario, endocytosed SVs remain trapped in an alkaline state, thereby preventing neurotransmitter reloading and rendering them unable to replenishment the RRP (Sankaranarayanan and Ryan, 2001). Results from the Bafi30 experiments showed a cumulative eIPSC amplitude of 927 nA, 33% of the Ctrl value (Figure 4E2). With a *q* value of 19 pA and an N_RRP_ of 125 SVs (Figures 6C1–C5,D2), this corresponds to ~49,000 released SVs and a 392-fold RRP turnover (compared to a 250-fold turnover in the Ctrl). Again, we conclude that such robust replenishment cannot be explained by recruitment from the reserve pool alone, nor by *de novo* synthesis. Transport of SVs from the soma to the axon terminal would occur on a time scale of hours, far too slow to sustain transmission under acute synaptic stress. In the following section, we will discuss an alternative scenario.

### An additional H^+^ pump system besides the V-ATPase is likely at MNTB-LSO synapses

**Figure 12:**
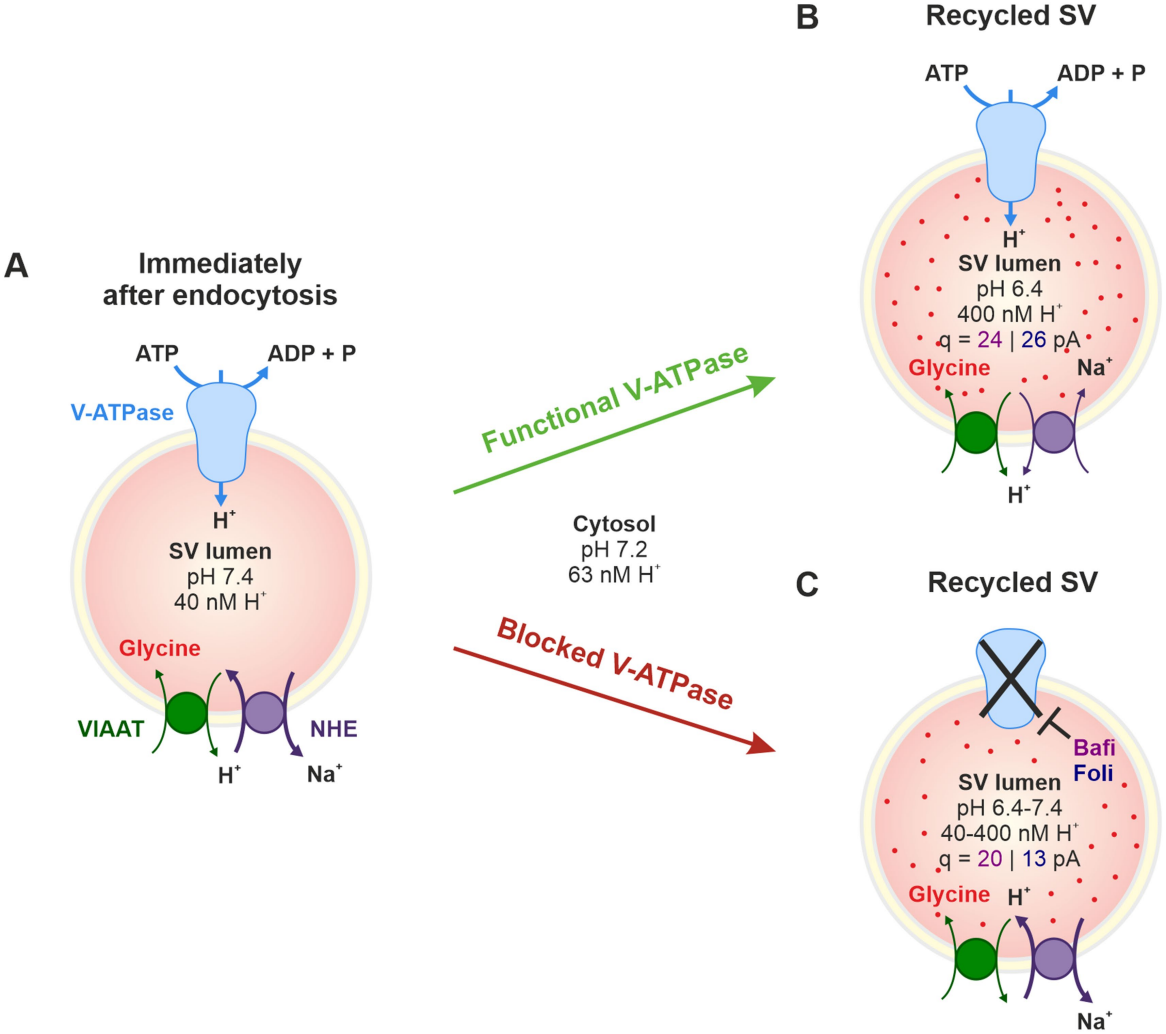
Schematic of the SV acidification mechanism proposed in the present study. **(A)** Immediately after endocytosis, the lumen of a recycling SV is filled with extracellular fluid (~150 mM Na^+^; pH = 7.4, corresponding to 40 nM H^+^). In addition, the SV is virtually free of glycine (no red dots). Key components for SV acidification and neurotransmitter filling are the V-ATPase (blue) and the VIAAT (green). By pumping H^+^ into the lumen, the V-ATPase generates an electrochemical gradient across the SV membrane which provides the driving force that is used by the VIAAT to load the SV with glycine, which is a zwitter ion. To regulate the electrochemical gradient, additional transport mechanisms are involved, such as H^+^/Na^+^ exchangers (NHE, purple). An interesting candidate is NHE6, which is highly immunopositive in glycinergic MNTB axon terminals (see Figure 8). We propose that NHE(6) operates in reverse mode immediately after endocytosis. Expelling Na^+^ from the SV while importing H^+^ promotes H^+^-coupled glycine antiport by VIAAT and thus glycine loading. **(B)** Under normal SV refilling conditions, V-ATPase-mediated H^+^ pumping results in an intraluminal pH of 6.4 (400 nM H^+^, Egashira et al., 2016). The present study revealed quantal sizes *q* of 24 pA in the Ctrl_Bafi_ cohort and of 26 pA in the Ctrl_Foli_ cohort (see Figure 6C3; Supplementary Figure S4E3). **(C)** The present study also revealed that blockade of V-ATPase activity with Bafi or Foli reduces, but does not abolish, sustained high-frequency transmission at MNTB-LSO synapses. Therefore, SV replenishment is not solely dependent on V-ATPase. V-ATPase blockade resulted in a ~ 20% lower *q* value in Bafi30 ($\frac{20\;\mathrm{pA}\;\text{Bafi}30}{24\;\mathrm{pA}\;\mathrm{Ctr}l_{\text{Bafi}}}$ = 83%; see Figure 6C3) and a 50% lower *q* value in Foli30 ($\frac{13\;\mathrm{pA}\;\text{Foli}30}{26\;\mathrm{pA}\;\mathrm{Ctr}l_{\text{Foli}}}$ = 50%; see Supplementary Figure S4E3). The scenario evaluates a hypothetical role for NHE(6) in intraluminal H^+^ transport. It provides an alternative mechanism for SV acidification that partially compensates for the blockade of V-ATPase function.

We propose that SVs are reacidified by additional H^+^ transporters besides the V-ATPases, and we suggest that NHEs are strong candidates (Figure 12). *Slc9a6*, the gene encoding NHE6, exhibited the highest expression level, similarly high as that of *Slc32a1* (VIAAT). Prominent immunoreactivity in MNTB axon terminals targeting LSO somata provides further support (Figure 8). Since NHE2–NHE5 expression was not detected in either region, NHE6 appears to be the most likely candidate. However, pharmacological attempts to block NHE activity with the antagonist EIPA were confounded by adverse effects on AP generation, preventing specific assessment. We acknowledge that functional evidence for NHE6’s role in SV acidification is lacking, and our immunohistochemical and gene expression data alone are insufficient to confirm its involvement.

We therefore propose the following hypothetical scenario, which aligns with existing literature across various synaptic systems (Kononenko and Haucke, 2015). After AP-triggered SV fusion and rapid transmitter release, SV membranes are retrieved and SVs via either classical clathrin-mediated endocytosis or clathrin-independent ultrafast endocytosis (Brockmann and Rosenmund, 2016; Eddings and Watanabe, 2025). The former occurs with a time constant of ~10 s, while the latter can proceed within hundreds of milliseconds (Dittman and Ryan, 2009; Soykan et al., 2017; Watanabe and Boucrot, 2017) or even faster (<100 ms; Watanabe et al., 2013; Delvendahl and Hallermann, 2016). In addition, activity-dependent bulk endocytosis, similar to micropinocytosis, can occur (Camblor-Perujo and Kononenko, 2021). Subsequently, reacidification takes place locally with time constants of 4–40 s (Atluri and Ryan, 2006; Egashira et al., 2015). NHEs could mediate rapid reacidification, particularly during heavy synaptic activity. In hippocampal synapses, a full exo-endocytosis cycle ~30 s (Fernández-Alfonso and Ryan, 2006), but can be as short as 15 s (Ryan and Smith, 1995; Hori and Takahashi, 2012). In the auditory brainstem, it may be faster. A recent article by Gallimore et al. (2025) provides further insight into the complete SV cycle.

As noted above, SVs refilling takes tens of seconds. One might argue that the rapid recovery observed after high-frequency stimulation in Bafi-treated synapses (within a few seconds; Figure 5) is inconsistent with this time course. However, steady-state amplitudes during challenge were determined in the s_50-60_ window. Therefore, SVs endocytosed early (e.g., at s_5-25_) could have recycled within 25–55 s, consistent with published data.

In our proposed dual-transporter model, vacuolar NHEs operate under highly favorable conditions for transporting H^+^ into the SV lumen. Immediately after endocytosis, the lumen contains extracellular fluid. Hence, luminal [Na^+^] (which was 153 mM in our ACSF) is ~30-fold higher than in the cytosol, driving Na^+^ efflux from the SVs. Because NHEs are antiporters, this drives H^+^ influx into the lumen, promoting reacidification. In addition, the luminal pH immediately after endocytosis is 7.4, compared to a cytosolic pH of 7.2, corresponding to a ~ 1.6-fold lower luminal [H^+^] (40 vs. 63 nM). This gradient also favors H^+^ entry into the lumen, an effect even more pronounced when V-ATPase activity is absent. Interestingly and perhaps surprisingly, a luminal pH of 7.4 corresponds to only 0.000275 free H^+^ ions per SV. A single free H^+^ ion in the SV lumen would result in pH 4 (Preobraschenski et al., 2014). However, the SV lumen has a buffer capacity of ~57 mM/pH, provided by vesicle proteins, ATP, and transmitter molecules (Edwards, 2007), which allows storage of ~1,200 H^+^ ions per SV (Egashira et al., 2015).

The equilibrium situation illustrated in Figure 1 is also relevant for NHE-mediated ion transport at rest. At a luminal pH of 6.4 versus a cytosolic pH of 7.2, the H^+^ gradient favors efflux from the SV. NHE activity under these conditions would dissipate the H^+^ gradient, counteract V-ATPase activity, and regulate alkalinization (Ouyang et al., 2013; Takamori, 2016). Further studies are needed to clarify the role of additional H^+^ transporters beyond V-ATPase in supplying protons to VIAAT, thereby ensuring sufficient SV filling, particularly during prolonged high-frequency activity. This issue applies to both inhibitory and excitatory synapses (Goh et al., 2011; Huang and Trussell, 2014; Lee et al., 2021).

Finally, in addition to NHE6, neurotransmitter transporter 4 (NTT4, also known as XT1 or B0AT3), might also contribute to SV refilling with glycine when the proton-motive force generated by the V-ATPase is diminished or absent. NTT4, encoded by *Slc6A17,* is exclusively expressed in the nervous system and associated with neuronal SVs (El Mestikawy et al., 1997; Fischer et al., 1999; Masson et al., 1999; Zaia and Reimer, 2009). Immunoreactivity has been demonstrated in both excitatory and inhibitory neurons (Hägglund et al., 2013). NTT4 catalyzes the transport of neutral amino acids, including glycine (Parra et al., 2008; Nicoli et al., 2025). Importantly, NTT4 is a sodium-dependent symporter that is inhibited by low pH (Zaia and Reimer, 2009), making it ideally suited for the initial stages of SV refilling (see Figure 12).

### Outlook

Several important questions remain open, leaving ample room for future experimentation. First, does sustained high-frequency stimulation after pharmacological inactivation of V-ATPase completely abolish neurotransmission in systems other than the MNTB-LSO synapses described here? Is the resilience in SV refilling unique to the auditory system, or does it extend to other robust, high-frequency synapses such as cerebellar mossy fiber-granule cell synapses (Delvendahl and Hallermann, 2016)? Could it even represent a more general property of synapses? Second, does NHE(6) directly contribute to refilling? This question requires functional evidence from assays employing specific NHE(6) antagonists or gene silencing. Third, are there additional vesicular transporters that maintain transmitter supply during high-frequency synaptic activity? Finally, can the robustness and resilience of neurotransmission at MNTB-LSO synapses be substantiated at the ultrastructural level using functional electron microscopy? Answering this question will require technically demanding “flash and freeze” or “zap and freeze” methods (Vandael and Jonas, 2024; Eddings and Watanabe, 2025).

## Data Availability

The original contributions presented in the study are included in the article/Supplementary material, further inquiries can be directed to the corresponding author.

## Glossary

*α*: significance level
ACSF: artificial cerebrospinal fluid
AP: action potential
Bafi: bafilomycin
BL: baseline
BSA: bovine serum albumin
Chal: challenge
CN: cochlear nucleus
CNS: central nervous system
Ctrl: Control condition (ACSF)
d: dorsal
DABCO: 1,4-diazabicyclo[2.2.2]octane
DMSO: dimethyl sulfoxide
EIPA: ethylisopropylamiloride
EtOH: ethanol
eIPSC_1_: evoked inhibitory postsynaptic current evoked by first stimulus
eIPSC: evoked inhibitory postsynaptic current
Foli: folimycin
*FR*: fractional recovery
GABA: *γ*-aminobutyric acid
GlyT2: glycine transporter 2
*I_RRP_*: cumulative eIPSC amplitude upon complete depletion of RRP
k: number of comparisons
l: lateral
LSO: lateral superior olive
*m*: quantal content
MNTB: medial nucleus of the trapezoid body
NHE: Na^+^/H^+^ exchanger
NTT4: sodium-dependent neutral amino acid transporter
*N_RRP_*: number of SVs in RRP
P: postnatal
PBS: phosphate-buffered saline
PFA: paraformaldehyde
*Pv*: release probability
*q*: quantal size
Rec: recording
Recov: recovery
*RRP*: readily releasable pool
RT: room temperature
SOC: superior olivary complex
SPN: superior paraolivary nucleus
Stim: stimulation
SV: synaptic vesicle
*TR*: transfer rate
V-ATPase: vacuolar (H^+^)-ATPase (H^+^ pump)
VGAT: vesicular GABA transporter
VIAAT: vesicular inhibitory amino acid transporter
