# Supplementary material for: Robust inhibitory glycinergic transmission and the effect of bafilomycin, folimycin and EIPA: lessons from the auditory brainstem
Source: Front Cell Neurosci. 2025 Oct 15;19:1625868. doi: 10.3389/fncel.2025.1625868 (PMC12568497; doi:10.3389/fncel.2025.1625868)
Supplement: Supplementary file 2 [file Data_Sheet_1.PDF]

Supplementary Tables

Supplementary Table S1 (related to Figure 3). Synaptic transmission in the presence of 2 µM Bafi

2 µM Bafi		Ctrl (ACSF)		Bafi10		Bafi30		Ctrl (ACSF)	Bafi10	Bafi30	Ctrl vs. Bafi10	Ctrl vs. Bafi30	
**Stimulation frequency [Hz]**		**mean**	**SEM**	**mean**	**SEM**	**mean**	**SEM**	**Number of cells**			**p-value**		**Figure**
**1**	**Baseline [pA]**	1023	72	914	99	354	56	11	11	9	0.358$^{n.s.}$	<.001***	3A5
**10**	**eIPSC$_1$ [pA]**	1163	77	997	140	392	65	11	11	9	0.490$^{n.s.}$	<.001***	3B2
	**s$_{0.9-1}$ [pA]**	716	72	601	86	242	41	11	11	9	0.378$^{n.s.}$	<.001***	3B3
	**s$_{9-10}$ [pA]**	670	65	583	87	231	41	11	11	9	0.532$^{n.s.}$	<.001***	3B4
	**s$_{50-60}$ [pA]**	633	62	505	70	202	43	11	11	9	0.196$^{n.s.}$	<.001***	3B5
**50**	**eIPSC$_1$ [pA]**	1255	124	940	104	345	60	11	11	9	0.074$^{n.s.}$	<.001***	3C2
	**s$_{0.9-1}$ [pA]**	575	76	432	45	145	30	11	11	9	**0.003**$^{**.}$	<.001***	3C3
	**s$_{9-10}$ [pA]**	475	46	386	39	131	29	11	11	9	**0.067**$^{n.s.}$	<.001***	3C4
	**s$_{50-60}$ [pA]**	267	48	238	46	88	29	11	11	9	0.647$^{n.s.}$	0.003**	3C5
**100**	**eIPSC$_1$ [pA]**	1222	139	918	107	335	75	11	11	9	0.094$^{n.s.}$	<.001***	3D2
	**s$_{0.9-1}$ [pA]**	401	43	288	43	109	29	11	11	9	0.132$^{n.s.}$	<.001***	3D3
	**s$_{9-10}$ [pA]**	99	22	191	36	81	24	11	11	9	0.130$^{n.s.}$	<.001***	3D4
	**s$_{50-60}$ [pA]**	86	21	62	19	25	9	11	11	9	**0.042**$^{n.s.}$	0.002**	3D5
**200**	**eIPSC$_1$ [pA]**	1048	122	791	76	315	76	11	11	9	0.139$^{n.s.}$	<.001***	3E2
	**s$_{0.9-1}$ [pA]**	191	38	154	24	55	14	11	11	9	0.354$^{n.s.}$	0.003**	3E3
	**s$_{9-10}$ [pA]**	47	16	25	7	18	6	11	11	9	**0.147**$^{n.s.}$	0.031$^{n.s.}$	3E4
	**s$_{50-60}$ [pA]**	29	14	18	5	10	3	11	11	9	**0.465**$^{n.s.}$	0.025$^{n.s.}$	3E5

Ctrl vs Bafi10: Paired 2-tailed t-test; Ctrl vs Bafi30: Unpaired 2-tailed t-test. If bold: Wilcoxon test. If underlined: Mann-Whitney test. Bonferroni post hoc correction (k = 2).

Supplementary Table S2 (related to Figure 4). Cumulative current integrals in the presence of 2 µM Bafi

**2 µM Bafi**		**Ctrl (ACSF)**		**Bafi10**		**Bafi30**		**Ctrl (ACSF)**	**Bafi10**	**Bafi30**	**Ctrl vs. Bafi10**	**Ctrl vs. Bafi30**	
**Stimulation frequency [Hz]**		**mean**	**SEM**	**mean**	**SEM**	**mean**	**SEM**	**Number of cells**			**p-value**		**Figure**
**10**	**eIPSCs60 [nA]**	394	36	325	46	128	25	11	11	9	0.189$^{n.s.}$	<.001***	4A2
**50**		1077	120	899	127	343	89	11	11	9	0.174$^{n.s.}$	<.001***	4B2
**100**		819	128	671	145	276	90	11	11	9	**0.054**$^{n.s.}$	0.002**	4C2
**200**		552	165	362	72	180	53	11	11	9	**0.123**$^{n.s.}$	0.012*	4D2
**10**	**TRs50-60 [nA/s]**	7	0.6	5	0.8	2	0.4	11	11	9	0.195$^{n.s.}$	<.001***	4A3
**50**		17	2	15	2	6	2	11	11	9	0.214$^{n.s.}$	<.001***	4B3
**100**		11	2	10	2	4	1	11	11	9	**0.175**$^{n.s.}$	0.007*	4C3
**200**		8	3	4	1	2	1	11	11	9	**0.039**$^{n.s.}$	0.020*	4D3
	**Cumulative 4 min [µA]**	2842	747	2256	473	927	311	11	11	9	0.147$^{n.s.}$	<.001***	4E2
**10**	**Cumulative 4 min [BL=1]**	381	11	358	30	344	21	11	11	9	**0.765**$^{n.s.}$	0.175$^{n.s.}$	4E3
**50**		1079	112	1009	99	854	120	11	11	9	0.561$^{n.s.}$	0.188$^{n.s.}$	
**100**		847	139	783	149	659	127	11	11	9	0.740$^{n.s.}$	0.342$^{n.s.}$	
**200**		575	173	419	77	449	76	11	11	9	**0.638**$^{n.s.}$	>.999$^{n.s.}$	
	**Cumulative 4 min [BL=1]**	2881	377	2569	299	2306	326	11	11	9	0.156$^{n.s.}$	0.275$^{n.s.}$	4E4

Ctrl vs Bafi10: Paired 2-tailed t-test; Ctrl vs Bafi30: Unpaired 2-tailed t-test. If bold: Wilcoxon test. If underlined: Mann-Whitney test. Bonferroni post hoc correction (k = 2).

Supplementary Table S3 (related to Figure 5). Recovery from synaptic depression in the presence of 2 µM Bafi

2 µM Bafi		Ctrl (ACSF)		Bafi10		Bafi30		Ctrl (ACSF)	Bafi10	Bafi30	Ctrl BL vs. Ctrl Recov	Bafi10 BL vs. Bafi10 Recov	Bafi30 BL vs. Bafi30 Recov	
**Stimulation frequency [Hz]**		**mean**	**SEM**	**mean**	**SEM**	**mean**	**SEM**	**Number of cells**			**p-value**			**Figure**
**10**	**RecovA [%]**	99	4	89	4	90	10	11	11	9	$0.996^{n.s.}$	0.029^{*}	0.027^{*}	5B3
**50**		103	9	87	7	68	10	11	11	9	$0.892^{n.s.}$	$0.072^{n.s.}$	0.008^{**}	5C3
**100**		89	8	84	9	64	9	11	11	9	$0.160^{n.s.}$	0.014^{*}	0.001^{***}	5D3
**200**		81	7	69	8	59	9	11	11	9	$0.280^{n.s.}$	0.008^{**}	0.002^{**}	5E3
											**Ctrl Chal vs. Ctrl Recov**	**Bafi10 Chal vs. Bafi10 Recov**	**Bafi30 Chal vs. Bafi30 Recov**	
											**p-value**			
**10**	**RecovB [x-fold]**	2	0.1	2	1	2	0.1	11	11	9	$<0.001^{***}$	**$<0.001^{***}$**	$<0.001^{***}$	5B4
**50**		5	1	5	1	4	1	11	11	9	$<0.001^{***}$	$<0.001^{***}$	**0.004^{**}**	5C4
**100**		14	2	21	6	14	3	11	11	9	$<0.001^{***}$	**$<0.001^{***}$**	**0.004^{**}**	5D4
**200**		71	14	44	11	45	13	11	11	9	**$<0.001^{**}$**	$<0.001^{***}$	**0.004^{**}**	5E4
											**Ctrl vs. Bafi10**	**Ctrl vs. Bafi30**		
											**p-value**			
**10**	**FR [%]**	111	14	74	9	69	9	11	11	8	**$0.054^{n.s.}$**	$\underline{0.033}^{n.s.}$		5B5
**50**		111	11	85	8	66	12	11	11	9	$0.087^{n.s.}$	*0.011^{*}*		5C5
**100**		97	7	84	11	64	9	11	11	9	$0.441^{n.s.}$	*0.009^{*}*		5D5
**200**		84	7	69	9	59	9	11	11	9	$0.227^{n.s.}$	*$0.046^{n.s.}$*		5E5

Paired 2-tailed t-test; unpaired 2-tailed t-test in *italic*. If bold: Wilcoxon test. If underlined: Mann-Whitney test. Bonferroni post hoc correction (F5B5, C5, D5, E5 k = 2).

Supplementary Table S4 (related to Figure 6). Synaptic parameters in the presence of 2 µM Bafi

**2 µM Bafi**		**Ctrl (ACSF)**		**Bafi10**		**Bafi30**		**Ctrl (ACSF)**	**Bafi10**	**Bafi30**	**Ctrl vs Bafi10**	**Ctrl vs Bafi30**	
**Stimulation frequency [Hz]**		**mean**	**SEM**	**mean**	**SEM**	**mean**	**SEM**	**Number of cells**			**p-value**		**Figure**
**100**	**I_{RRP} [nA]**	11.3	2	8.5	1	2.4	1	11	11	9	**0.102**$^{n.s.}$	<0.001***	6C2
	**N_{RRP} [SV]**	434		389		125		11	11	9	0.715$^{n.s.}$	0.014*	6C2
**1**	**q [pA]**	26	2	27	3	20	1	11	11	9	0.188$^{n.s.}$	0.060$^{n.s.}$	6C3
**100**	**m [SV]**	47	7	35	4	21	5	11	11	9	0.126$^{n.s.}$	0.014*	6C4
**100**	**P_v [%]**	11	0.1	12	0.1	16	0.2	11	11	9	0.794$^{n.s.}$	0.025*	6C5

Ctrl vs Bafi10: Paired 2-tailed t-test; Ctrl vs Bafi30: Unpaired 2-tailed t-test. If bold: Wilcoxon test. If underlined: Mann-Whitney test. Bonferroni post hoc correction (k = 2).

Supplementary Table S5 (related to Figure 7). Synaptic transmission in the presence of 5 µM Bafi

**5 µM Bafi**		**Ctrl (ACSF)**		**Bafi10**		**Bafi30**		**Ctrl (ACSF)**	**Bafi10**	**Bafi30**	**Ctrl vs. Bafi10**	**Ctrl vs. Bafi30**		
**Stimulation frequency [Hz]**		**mean**	**SEM**	**mean**	**SEM**	**mean**	**SEM**	**Number of cells**			**p-value**			**Figure**
**100**	**Amplitudes s_{50-60} [pA]**	55	13	38	10	18	9	9	9	8	0.134$^{n.s.}$	0.021*		7A2
	**Amplitudes $s_{110-120}$ [pA]**	58	13	34	8	19	8	9	9	8	0.046$^{n.s.}$	0.008*		
	**Amplitudes $s_{170-180}$ [pA]**	66	15	30	7	20	7	9	9	8	**0.020***	0.011*		
	**Amplitudes $s_{230-240}$ [pA]**	51	11	25	7	17	6	9	9	8	**0.008***	0.011*		
	**Cumulative [µA]**	1.6	0.3	1.0	0.2	0.5	0.2	9	9	8	0.032$^{n.s.}$	*0.020**		7B2
	**TRs$_{230-240}$ [nA/s]**	6	1	3	0.9	2	0.8	9	9	8	**0.027**$^{n.s.}$	0.004**		7B3
											**Ctrl BL vs. Ctrl Recov3**	**Bafi10 BL vs. Bafi10 Recov3**	**Bafi30 BL vs. Bafi30 Recov3**	
											**p-value**			
**100**	**RecovA [%]**	107	9	63	10	68	9	9	9	8	0.705$^{n.s.}$	0.015*	0.001**	7C2
											**Ctrl Chal vs. Ctrl Recov**	**Bafi10 Chal vs. Bafi10 Recov**	**Bafi30 Chal vs. Bafi30 Recov**	
											**p-value**			
**100**	**RecovB [x-fold]**	24	5	23	4	16	4	9	9	8	**0.004****	**0.004****	0.061$^{n.s.}$	7C3
											**p-value**			
**100**	**FR [%]**	107	9	61	17	46	23	9	9	8	0.037$^{n.s.}$	*0.022**		7C4

Paired 2-tailed t-test; unpaired 2-tailed t-test in *italic*. If bold: Wilcoxon test. If underlined: Mann-Whitney test. Bonferroni post hoc correction (k = 2, except of 7C2 k = 1).

Supplementary Table S6 (related to Supplementary Figure S1). Synaptic transmission in the presence of the solvent EtOH

**EtOH**		**Ctrl (ACSF)**		**EtOH10**		**EtOH30**		**Ctrl (ACSF)**	**EtOH10**	**EtOH30**	**Ctrl vs. EtOH10**	**Ctrl vs. EtOH30**		
**Stimulation frequency [Hz]**		**mean**	**SEM**	**mean**	**SEM**	**mean**	**SEM**	**Number of cells**			**p-value**			**Figure**
**1**	**Baseline [pA]**	1029	139	1005	291	936	125	6	6	7	0.153$^{n.s.}$	*0.923*$^{n.s.}$		S1A5
**10**	**s50-60 [pA]**	597	93	683	180	634	91	6	6	7	0.503$^{n.s.}$	*0.784*$^{n.s.}$		S1B2
**50**		257	64	354	127	319	98	6	6	7	0.244$^{n.s.}$	*0.619*$^{n.s.}$		S1C2
**100**		70	11	97	40	110	32	6	6	7	**>0.999**$^{n.s.}$	*0.295*$^{n.s.}$		S1D2
**200**		19	4	20	4	33	9	6	6	7	0.796$^{n.s.}$	*0.207*$^{n.s.}$		S1E2
		**Ctrl (ACSF)**		**EtOH10**		**EtOH30**		**Ctrl (ACSF)**	**EtOH10**	**EtOH30**	**Ctrl BL vs. Ctrl Recov**	**EtOH10 BL vs. EtOH10 Recov**	**EtOH30 BL vs. EtOH30 Recov**	
												**p-value**		
**10**	**RecovA [%]**	93	14	100	11	107	19	6	6	7	**0.438**$^{n.s.}$	**>0.999**$^{n.s.}$	**0.281**$^{n.s.}$	S1F2
**50**		109	24	110	8	120	43	6	6	7	**0.313**$^{n.s.}$	**0.063**$^{n.s.}$	**0.281**$^{n.s.}$	S1G2
**100**		99	28	101	14	110	50	6	6	7	**>0.999**$^{n.s.}$	**>0.999**$^{n.s.}$	**0.547**$^{n.s.}$	S1H2
**200**		87	36	85	25	101	52	6	6	7	**0.438**$^{n.s.}$	**0.688**$^{n.s.}$	**0.656**$^{n.s.}$	S1I2
											**Ctrl vs. EtOH10**	**Ctrl vs. EtOH30**		
											**p-value**			
**10**	**FR [%]**	88	29	104	46	142	104	6	6	7	0.349$^{n.s.}$	0.221$^{n.s.}$		S1F3
**50**		116	32	115	12	161	107	6	6	7	0.948$^{n.s.}$	*0.339*$^{n.s.}$		S1G3
**100**		99	30	100	16	125	60	6	6	7	0.897$^{n.s.}$	*0.362*$^{n.s.}$		S1H3
**200**		88	37	85	25	108	52	6	6	7	**0.563**$^{n.s.}$	*0.440*$^{n.s.}$		S1I3

Paired 2-tailed t-test; unpaired 2-tailed t-test in *italic*. If bold: Wilcoxon test. If underlined: Mann-Whitney test. Bonferroni post hoc correction (k = 2, except of S1F2-I2 k = 1).

Supplementary Table S7 (related to Supplementary Figure S2). Synaptic transmission in the presence of Bafi or EtOH alone

**Comparison Bafi and EtOH**		**Ctrl EtOH**		**Ctrl Bafi**		**Ctrl EtOH**	**Ctrl Bafi**	**Ctrl EtOH vs. Ctrl Bafi**	
**Stimulation frequency [Hz]**		**mean**	**SEM**	**mean**	**SEM**	**Number of cells**		**p-value**	**Figure**
**10**	**Chal s_{50-60} [pA]**	597	93	634	62	6	11	0.742$^{n.s.}$	S2A2
**50**		257	64	266	48	6	11	<u>0.808</u>$^{n.s.}$	
**100**		70	11	86	21	6	11	<u>>0.999</u>$^{n.s.}$	
**200**		19	4	29	14	6	11	<u>0.961</u>$^{n.s.}$	
**10**	**Recov s_{50-60} [pA]**	947	143	1023	95	6	11	0.656$^{n.s.}$	S2A3
**50**		1110	184	1036	109	6	11	0.717$^{n.s.}$	
**100**		1002	191	899	91	6	11	0.589$^{n.s.}$	
**200**		862	186	812	71	6	11	<u>0.591</u>$^{n.s.}$	
		**EtOH10**		**Bafi10**		**EtOH10**	**Bafi10**	**EtOH10 vs. Bafi10**	
**Stimulation frequency [Hz]**		**mean**	**SEM**	**mean**	**SEM**	**Number of cells**		**p-value**	**Figure**
**10**	**Chal s_{50-60} [pA]**	683	180	505	70	6	11	<u>0.733</u>$^{n.s.}$	S2B2
**50**		354	127	238	46	6	11	0.310$^{n.s.}$	
**100**		97	40	62	19	6	11	<u>0.350</u>$^{n.s.}$	
**200**		20	4	18	5	6	11	<u>0.525</u>$^{n.s.}$	
**10**	**Recov s_{50-60} (pA)**	1046	322	802	85	6	11	0.362$^{n.s.}$	S2B3
**50**		1128	342	762	87	6	11	0.200$^{n.s.}$	
**100**		1052	329	700	58	6	11	0.178$^{n.s.}$	
**200**		820	188	598	83	6	11	0.229$^{n.s.}$	
		**EtOH30**		**Bafi30**		**EtOH30**	**Bafi30**	**EtOH30 vs. Bafi30**	
**Stimulation frequency [Hz]**		**mean**	**SEM**	**mean**	**SEM**	**Number of cells**		**p-value**	**Figure**
**10**	**Chal s_{50-60} [pA]**	634	91	202	43	7	9	<0.001***	S2C2
**50**		319	98	88	29	7	9	<u>0.040</u>*	
**100**		110	32	25	9	7	9	<u>0.030</u>*	
**200**		33	9	2	0.4	7	9	0.001**	
**10**	**Recov s_{50-60} [pA)**	1063	150	305	54	7	9	<0.001***	S2C3
**50**		1242	222	278	70	7	9	<0.001***	
**100**		1152	227	287	68	7	9	0.003**	
**200**		1024	220	227	50	7	9	0.001**	

Unpaired 2-tailed t-test, if underlined: Mann-Whitney test. Bonferroni post hoc correction (k = 2).

Supplementary Table S8 (related to Supplementary Figure S3). Synaptic transmission in the presence of 1 µM Foli

**1 µM Foli**		**Ctrl (ACSF)**		**Foli10**		**Foli30**		**Ctrl (ACSF)**	**Foli10**	**Foli30**	**Ctrl vs Foli10**	**Ctrl vs Foli30**	
**Stimulation frequency [Hz]**		**mean**	**SEM**	**mean**	**SEM**	**mean**	**SEM**	**Number of cells**			**p-value**		**Figure**
**1**	**Baseline [pA]**	1321	176	1151	164	633	196	13	13	8	0.146$^{n.s.}$	0.002**	S3A5
**10**	**eIPSC$_1$ [pA]**	1432	178	1282	177	704	219	13	13	8	0.189$^{n.s.}$	0.003**	S3B2
	**s$_{0.9\text{-}1}$ [pA]**	1004	131	889	122	470	136	13	13	8	0.229$^{n.s.}$	0.003**	S3B3
	**s$_{9\text{-}10}$ [pA]**	956	124	818	116	414	122	13	13	8	0.164$^{n.s.}$	0.002**	S3B4
	**s$_{50\text{-}60}$ [pA]**	909	121	811	120	397	128	13	13	8	0.278$^{n.s.}$	0.005**	S3B5
**50**	**eIPSC$_1$ [pA]**	1380	192	1222	180	606	193	13	13	8	0.331$^{n.s.}$	0.005**	S3C2
	**s$_{0.9\text{-}1}$ [pA]**	807	102	712	109	300	95	13	13	8	0.248$^{n.s.}$	<0.001**	S3C3
	**s$_{9\text{-}10}$ [pA]**	661	89	569	91	220	53	13	13	8	0.171$^{n.s.}$	<0.001***	S3C4
	**s$_{50\text{-}60}$ [pA]**	504	80	447	82	126	29	13	13	8	0.421$^{n.s.}$	0.002**	S3C5
**100**	**eIPSC$_1$ [pA]**	1521	249	1315	205	537	145	13	13	8	**0.273**$^{n.s.}$	<0.001***	S3D2
	**s$_{0.9\text{-}1}$ [pA]**	618	65	512	94	177	40	13	13	8	**0.110**$^{n.s.}$	<0.001***	S3D3
	**s$_{9\text{-}10}$ [pA]**	359	48	306	61	90	20	13	13	8	**0.340**$^{n.s.}$	<0.001***	S3D4
	**s$_{50\text{-}60}$ [pA]**	144	28	109	19	31	8	13	13	8	0.134$^{n.s.}$	0.007*	S3D5
**200**	**eIPSC$_1$ [pA]**	1487	247	1202	193	485	139	13	13	8	**0.191**$^{n.s.}$	<0.001**	S3E2
	**s$_{0.9\text{-}1}$ [pA]**	254	35	193	45	57	16	13	13	8	**0.080**$^{n.s.}$	<0.001***	S3E3
	**s$_{9\text{-}10}$ [pA]**	74	21	65	18	11	2	13	13	8	**0.340**$^{n.s.}$	<0.001**	S3E4
	**s$_{50\text{-}60}$ [pA]**	51	14	32	7	10	2	13	13	8	**0.033**$^{n.s.}$	<0.001***	S3E5

Ctrl vs. Foli10: Paired 2-tailed t-test; Ctrl vs Foli30: Unpaired 2-tailed t-test. If bold: Wilcoxon test. If underlined: Mann-Whitney test. Bonferroni post hoc correction (k = 2).

Supplementary Table S10 (related to Supplementary Figure S4). Recovery from synaptic depression in the presence of 1 µM Foli

**1 µM Foli**		**Ctrl (ACSF)**		**Foli10**		**Foli30**		**Ctrl (ACSF)**	**Foli10**	**Foli30**	**Ctrl BL vs Ctrl Recov**	**Foli10 BL vs Foli10 Recov**	**Foli30 BL vs Foli30 Recov**	
**Stimulation frequency [Hz]**		**mean**	**SEM**	**mean**	**SEM**	**mean**	**SEM**	**Number of cells**			**p-value**			**Figure**
**10**	**RecovA [%]**	92	12	87	29	52	31	13	13	8	**0.040**$^{n.s.}$	0.097$^{n.s.}$	**0.023***	S4A2
**50**		100	18	87	30	46	22	13	13	8	**0.685**$^{n.s.}$	**0.132**$^{n.s.}$	**0.008****	S4B2
**100**		96	16	80	30	42	22	13	13	8	**0.748**$^{n.s.}$	0.067$^{n.s.}$	**0.008****	S4C2
**200**		93	16	72	25	43	22	13	13	8	**0.273**$^{n.s.}$	0.011*	0.007**	S4D2
											**Ctrl Chal vs. Ctrl Recov**	**Foli10 Chal vs. Foli10 Recov**	**Foli30 Chal vs. Foli30 Recov**	
											**p-value**			
**10**	**RecovB [x-fold]**	1	0.1	1	0.1	2	3	13	13	8	<0.001***	<0.001***	**0.008****	S4A3
**50**		3	1	3	1	8	12	13	13	8	**<0.001*****	**<0.001*****	**0.008****	S4B3
**100**		14	11	12	6	35	49	13	13	8	**<0.001*****	<0.001***	**0.008****	S4C3
**200**		36	18	49	37	67	56	13	13	8	**<0.001*****	**<0.001*****	0.003**	S4D3
											**Ctrl vs. Foli10**	**Ctrl vs. Foli30**		
											**p-value**			
**10**	**FR [%]**	81	29	128	237	43	59	13	13	8	**0.424**$^{n.s.}$	0.01*		S4A4
**50**		103	27	90	55	40	23	13	13	8	**0.340**$^{n.s.}$	*<0.001****		S4B4
**100**		96	17	79	33	40	22	13	13	8	0.142$^{n.s.}$	<0.001***		S4C4
**200**		92	16	72	25	43	22	13	13	8	0.045$^{n.s.}$	<0.001**		S4D4
**100**	**I_{RRP} [nA]**	19	4	14	2	5	1	13	13	8	**0.057**$^{n.s.}$	<0.001***		S4E2
	**N_{RRP} [SV]**	832		620		374		13	13	8				
**1**	**q [pA]**	24	2	23	2	13	1	13	13	8	0.643$^{n.s.}$	*0.0048***		S4E3
**100**	**m [SV]**	64	6	56	5	38	6	13	13	8	**0.068**$^{n.s.}$	0.008*		S4E4
**100**	**P_v [%]**	10	1	9	1	11	1	13	13	8	0.682$^{n.s.}$	*0.422*$^{n.s.}$		S4E5

Paired 2-tailed t-test; *unpaired 2-tailed t-test* in *italic*. If bold: Wilcoxon test. If underlined: Mann-Whitney test. Bonferroni post hoc correction (S4A4, B4, C4, D4, E2-E5 k = 2).

Supplementary Table S11 (related to Figure 9). Synaptic transmission in the presence of 100 µM EIPA

**100 µM EIPA**		**Ctrl (ACSF)**		**EIPA10**		**Ctrl (ACSF)**	**EIPA10**	**Ctrl vs. EIPA10**		
**Stimulation frequency [Hz]**		**mean**	**SEM**	**mean**	**SEM**	**Number of cells**		**p-value**		**Figure**
**1**	**Baseline**	1450	205	799	251	8	8	<0.001***		9A4
**50**	**s50-60**	439	120	109	41	8	8	0.009**		9B2
**100**		131	43	34	11	8	8	0.022*		9D2
		**Ctrl (ACSF)**		**EIPA10**		**Ctrl (ACSF)**	**EIPA10**	**Ctrl BL vs. Ctrl Recov**	**EIPA10 BL vs. EIPA10 Recov**	**Figure**
								**p-value**		
**50**	**RecovA [%]**	111	22	72	18	8	8	0.087$^{n.s.}$	0.282$^{n.s.}$	9C2
**100**		104	28	58	18	8	8	0.282$^{n.s.}$	0.018*	9E2
								**Ctrl vs. EIPA10**		
								**p-value**		
**50**	**FR [%]**	119	11	68	6	8	8	0.005**		9C3
**100**		106	12	56	7	8	8	0.006*		9E3
		**Ctrl (ACSF)**		**EIPA10**		**Ctrl (ACSF)**	**EIPA10**	**Ctrl vs. EIPA10**		
**Stimulation frequency [Hz]**	**Amplitudes [pA]**	**mean**	**SEM**	**mean**	**SEM**	**Number of cells**		**p-value**		**Figure**
**100**	**I_{RRP} [nA]**	31	14	7	3	8	8	**0.008****		9F2
	**N_{RRP} [SV]**	930		254		8	8			
**1 (Recov2)**	**q [pA]**	27	3	32	4	8	8	0.077$^{n.s.}$		9F3
**100**	**m [SV]**	67	10	24	6	8	8	<0.001***		9F4
**100**	**P_v [%]**	11	2	12	2	8	8	0.881$^{n.s.}$		9F5

Paired 2-tailed t-test. If bold: Wilcoxon test. Bonferroni post hoc correction (k = 2 except for Fig. 9C2 and Fig. 9E2, where k = 1).

Supplementary Table S12 (related to Figure 10). Effect of 100 µM EIPA on the AP fidelity of MNTB neurons

**100 µM EIPA**		**Ctrl (ACSF)**		**EIPA10**		**Ctrl (ACSF)**	**EIPA10**	**Ctrl vs. EIPA10**					
**Stimulation frequency [Hz]**	**AP Fidelity [%]**	**mean**	**SEM**	**mean**	**SEM**	**Number of cells**		**p-value**					**Figure**
**50**	s_{50-60}	86	10	24	13	9	9	**0.004****					10E
**100**		74	13	11	11	9	9	**0.023***					
		**Ctrl (ACSF)**		**Bafi10**		**Bafi30**		**Ctrl (ACSF)**	**Bafi10**	**Bafi30**	**Ctrl vs. Bafi10**	**Ctrl vs. Bafi30**	
**Stimulation frequency [Hz]**	**AP Fidelity [%]**	**mean**	**SEM**	**mean**	**SEM**	**mean**	**SEM**	**Number of cells**			**p-value**		
**10**	s_{50-60}	100	0	89	11	88	8	7	7	8	**>0.999**$^{n.s.}$	0.20$^{n.s.}$	10G
**50**		84	12	82	13	66	14	7	7	8	**>0.999**$^{n.s.}$	0.368$^{n.s.}$	
**100**		70	13	61	19	44	14	7	7	8	**0.875**$^{n.s.}$	*0.213*$^{n.s.}$	
**200**		33	14	35	13	19	11	7	7	8	**0.863**$^{n.s.}$	0.885$^{n.s.}$	

Paired 2-tailed t-test; unpaired 2-tailed t-test in *italic*. If bold: Wilcoxon test. If underlined: Mann-Whitney test. Bonferroni post hoc correction (Fig. 10E: k = 1, Fig. 10G: k = 2).

Supplementary Table S13 (related to Figure 11). Synaptic transmission in the presence of Bafi or EIPA after AP failure correction

AP failure correction		Ctrl (ACSF)	Bafi10	Bafi30	Bafi10/Ctrl [%]	Bafi30/Ctrl [%]	
**Stimulation frequency [Hz]**	**Mean amplitudes**	**mean**	**mean**	**mean**			**Figure**
**10**	**Corrected [pA]**	633	567	230	90	**<u>36</u>**	not shown
	**Uncorrected [pA]**	633	505	202	80	32	
**50**	**Corrected [pA]**	317	290	133	91	**<u>42</u>**	11A1
	**Uncorrected [pA]**	267	238	88	89	33	11A2
**100**	**Corrected [pA]**	123	102	57	83	**<u>46</u>**	11C1
	**Uncorrected [pA]**	86	62	25	72	29	11C2
**200**	**Corrected [pA]**	88	51	53	59	60	not shown
	**Uncorrected [pA]**	29	18	10	62	34	
		**Ctrl (ACSF)**	**EIPA10**		**EIPA10/Ctrl**		
**Stimulation frequency [Hz]**	**Mean amplitudes**	**mean**	**mean**		**[%]**		
**50**	**Corrected [pA]**	510	454		89		11B1
	**Uncorrected [pA]**	439	109		25		11B2
**100**	**Corrected [pA]**	177	309		175		11D1
	**Uncorrected [pA]**	131	34		26		11D2

Comparison of the effects of Bafi or EIPA obtained without and with correction for AP failures occurring in MNTB neurons at high stimulation frequencies. These AP failures were determined from MNTB neurons whose axons were antidromically stimulated at various frequencies ranging from 10 to 200 Hz (cf. and Figure 10E,G1-G4). The drug effects were then compared with the corresponding Ctrl results and needed to fulfill the following arbitrary threshold criterion: a drug caused synaptic depression when the ratio $\frac{drug}{Ctrl} < 50\%$. This criterion was fulfilled only by the Bafi30/Ctrl ratios at stimulation frequencies of 10, 50 and 100 Hz (see numbers in bold and underlined). A drug caused synaptic facilitation when the ratio $\frac{drug}{Ctrl} > 200\%$. This criterion was never fulfilled. Upon AP failure correction, the originally observed EIPA10 effects (25% and 26%) were considered unreliable and were therefore rejected. The corrected mean amplitudes of the eIPSCs were derived from Figure 11A1,B1,C1,D1, whereas the uncorrected mean amplitudes of the eIPSCs were obtained from Figure 3B1,C1,D1,E1 and Figure 9B1,D1. They represent the $s_{50\text{-}60}$ period.
