# Supplementary material for: Robust inhibitory glycinergic transmission and the effect of bafilomycin, folimycin and EIPA: lessons from the auditory brainstem
Source: Front Cell Neurosci. 2025 Oct 15;19:1625868. doi: 10.3389/fncel.2025.1625868 (PMC12568497; doi:10.3389/fncel.2025.1625868)
Supplement: Supplementary file 3 [file Data_Sheet_2.PDF]

Supplementary Figures

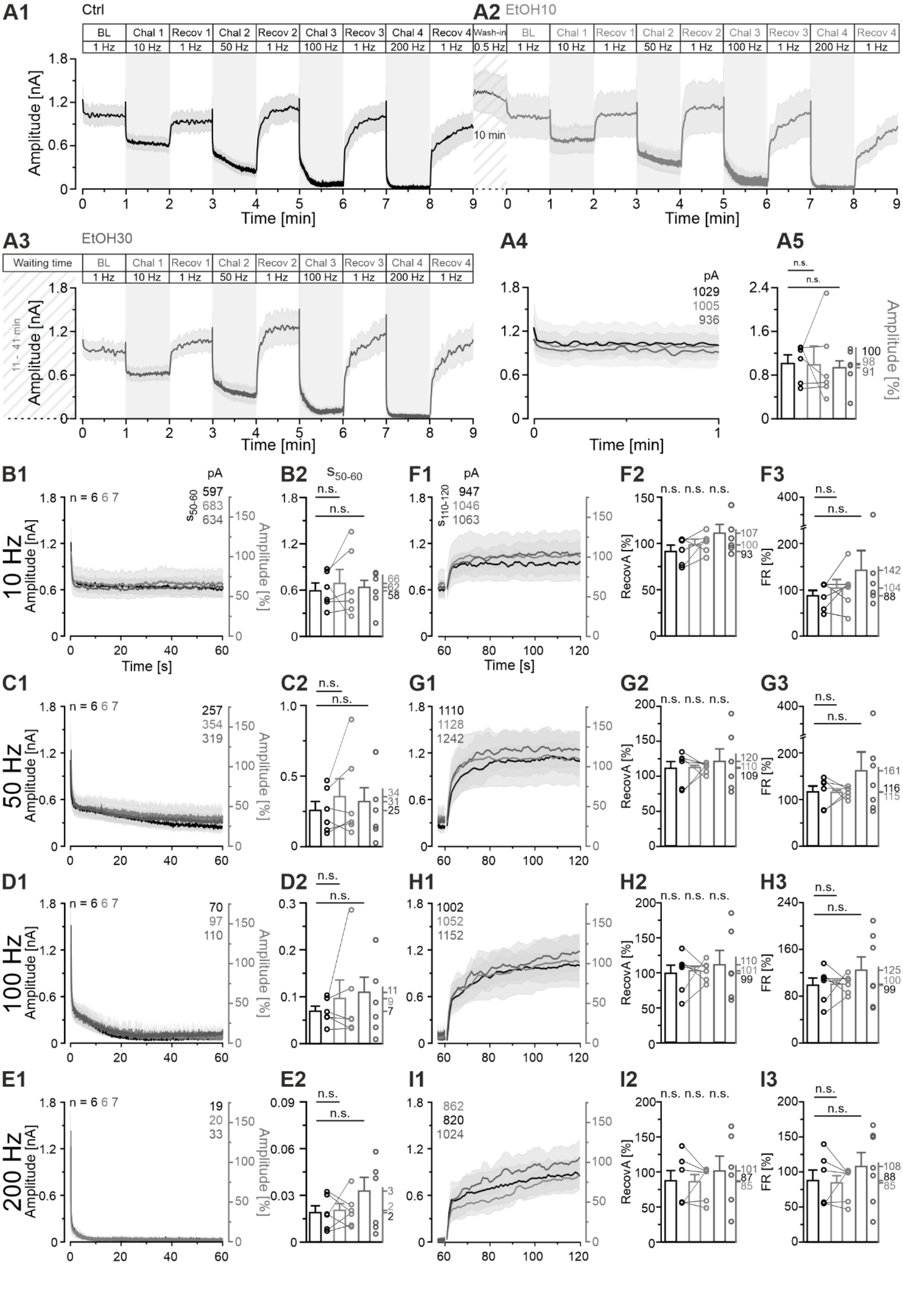

Supplementary Figure S1 - Synaptic transmission is unaffected when EtOH, the solvent for Bafi, is applied alone. (A1-A2) eIPSC peak amplitudes (mean ± SEM) for Ctrl (black) and 0.1% EtOH10

(light gray) obtained from paired recordings with Protocol 1 (see Figure 1C) in sham control experiments. **(A3)** Time course for 0.1% EtOH30 (dark gray). Synaptic performance after EtOH treatment is indistinguishable from the Ctrl. Data in A3 are from a different neuron than in A1 and A2. Diagonally striped regions indicate perfusion periods of 0.1% EtOH. **(A4)** Close-up of the superimposed BL traces. Mean values are shown in the upper right corner. **(A5)** Statistics for BL analysis. EtOH10 and EtOH30 BL are not statistically different from the Ctrl. **(B1)** Time course of absolute and normalized eIPSC peak amplitudes during the 10-Hz|60-s challenge period. Numbers in the upper right indicate mean amplitudes at $s_{50\text{-}60}$. **(B2)** Statistics of amplitudes at $s_{50\text{-}60}$. **(C-E)** Same as B, but for 50 **(C1-C2)**, 100 **(D1-D2)** and 200-Hz challenge **(E1-E2)**. eIPSC amplitudes at $s_{50\text{-}60}$ in EtOH10 and EtOH30 do not differ statistically from the Ctrl. **(F1)** Time course of absolute and normalized amplitudes during 1-Hz|60-s recovery after 10-Hz challenge. Upper left numbers indicate the mean amplitudes at $s_{110\text{-}120}$. Recovery from synaptic depression remains robust and efficient, even after 9 min of stimulation with 21,900 stimulus pulses in the presence of EtOH. **(F2-F3)** Statistics for RecovA **(F2)** and *FR* **(F3)**. Mean percentages shown on right y-axis. **(G-I)** Same as F, but for recovery after 50 **(G1-G3)**, 100 **(H1-H3)** and 200-Hz challenge **(I1-I3)**. RecovA does not differ significantly from the BL, indicating complete recovery. Furthermore, *FR* in EtOH is statistically indistinguishable from the Ctrl, indicating unaffected replenishment. Variables on the left and right y-axes in C1-E1, B2-E2 and F1-I1 are the same as in B1. Variables on the left y-axes in G2-I2 and G3-I3 are the same as in F2 and F3, respectively. Time courses are weighted moving averages (SEM lightly shaded). Ctrl and EtOH10, n = 6; EtOH30, n = 7. See Supplementary Table S6 for details, including statistics.

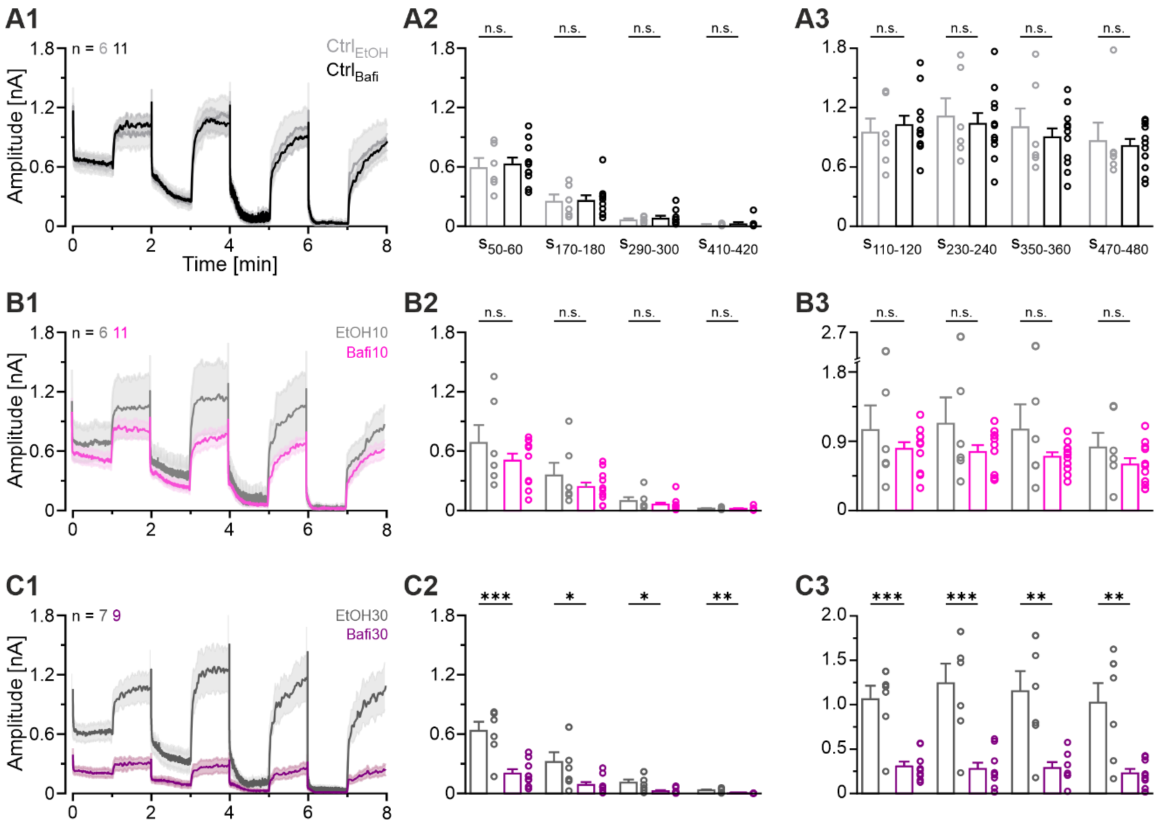

Supplementary Figure S2 - The decrease in eIPSC amplitudes upon Bafi application is drug-specific and not time-dependent. (A1) Time course of eIPSC peak amplitudes (mean ± SEM) for $Ctrl_{EtOH}$ (before EtOH application, light gray) and $Ctrl_{Bafi}$ (before Bafi application, black; see Figure 3) obtained with Protocol 1 (see Figure 1C). Traces are superimposed for better comparison. Note the virtual overlap of the two traces, demonstrating very close reproducibility. **(A2)** Statistics for the last 10 s of the steady-state depression levels shown in A1. None of the four comparisons showed a significant difference. **(A3)** Statistics for the last 10 s of the recovery periods shown in A1. None of the four comparisons showed a significant difference. $Ctrl_{EtOH}$, n = 6; $Ctrl_{Bafi}$, n = 11. **(B1-B3)** Same as A1-A3, but for EtOH10_vs_Bafi10 (light gray and magenta, respectively). In none of the eight comparisons did we find a significant difference, indicating that Bafi was not yet effective after a 10-min wash-in. EtOH10, n = 6; Bafi10, n = 11. **(C1-C3)** Same as A1-A3, but for EtOH30_vs_Bafi30 (dark gray and purple, respectively). Here, each of the eight comparisons showed a significant difference, indicating that Bafi was effective after a 30-min perfusion. EtOH30, n = 7; Bafi30, n = 9. Variables on the left y-axis in A2-C3 are the same as in A1. Time courses represent weighted moving averages (SEM lightly shaded). See Supplementary Table S7 for details, including statistics.

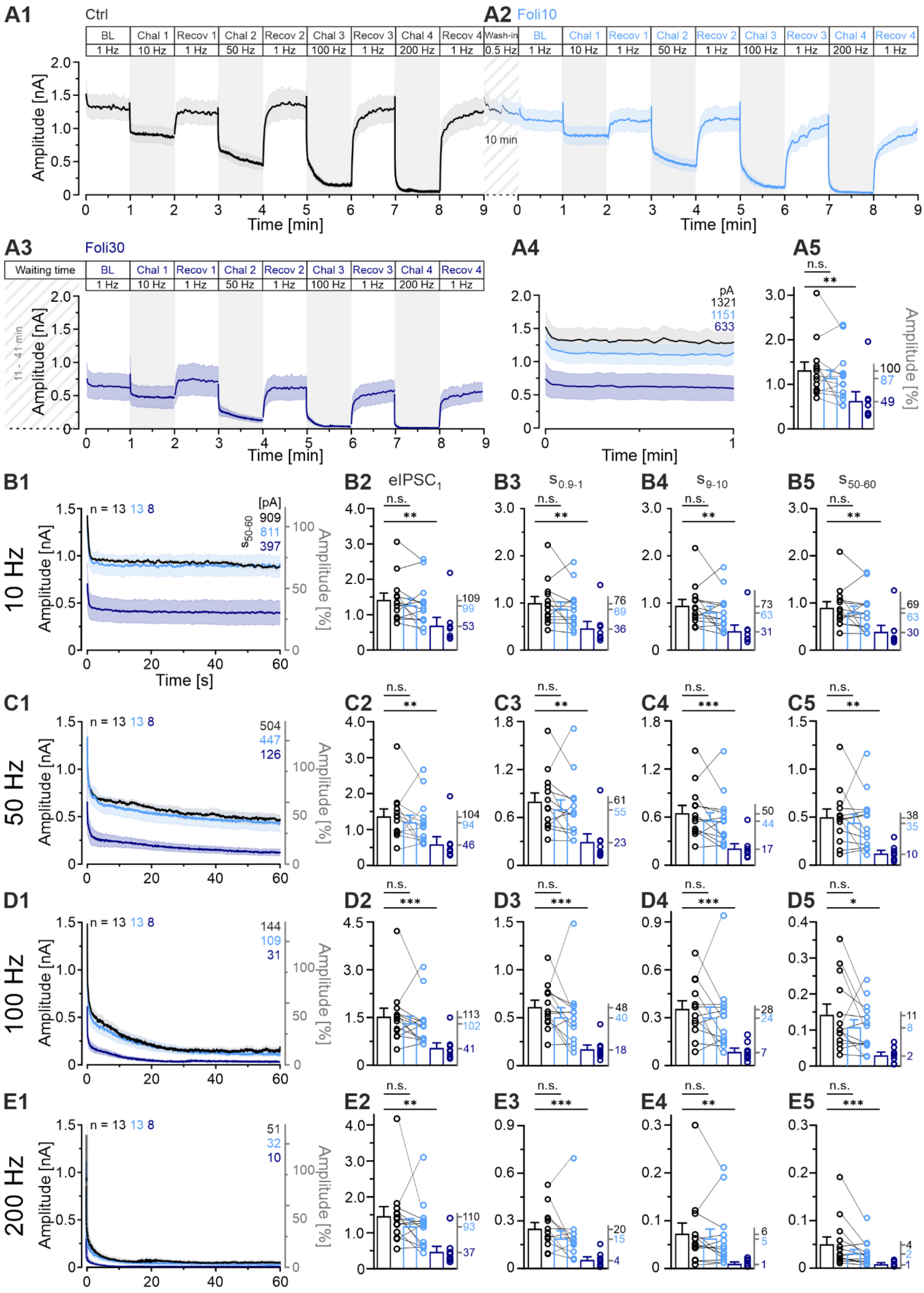

Supplementary Figure S3 - Bafilomycin effects are reproduced by folimycin. (A1 - A2) Time course of eIPSC peak amplitudes (mean ± SEM) for Ctrl (black) and Foli10 (light blue) obtained from paired recordings using Protocol 1 (see Figure 1C). **(A3)** Time course for Foli30 (blue). Data in A3 are from a different neuron than in A1 and A2. Diagonally striped regions indicate perfusion periods for 1 µM Foli. **(A4)** Close-up of the superimposed BL traces. Mean values are shown in the upper right corner. **(A5)**

Statistics for BL (mean: Ctrl: 1,321 pA = 100%; Foli10: 1,151 pA = 87%; Foli30: 633 pA = 49%). Similar to Bafi, BL levels are comparable between Ctrl and after 10-min wash-in of Foli, but they are significantly reduced after 30-min perfusion. **(B1)** Time course of absolute and normalized eIPSC peak amplitudes during 10-Hz|60-s challenge. Upper right numbers indicate mean amplitudes at s_{50-60}. Foli10 is not sufficient to substantially reduce synaptic transmission, whereas Foli30 induces a significant decrease at all stimulation frequencies. **(B2-B5)** Statistics at four time windows ($eIPSC_1$, $s_{0.9-1}$, s_{9-10}, s_{50-60}). Mean percentages shown on right y-axis. **(C-E)** Same as B, but for 50 Hz **(C1-C5)**, 100 Hz **(D1-D5)** and 200 Hz **(E1-E5)**. None of the 16 comparisons between Ctrl and Foli10 show significant differences. In contrast, Foli30 shows significantly reduced eIPSC amplitudes in all comparisons. Variables on the left and right y-axes are the same as in B1. Time courses are weighted moving averages (SEM lightly shaded). Ctrl and Foli10, n = 13; Foli30, n = 8. See Supplementary Table S8 for details, including statistics.

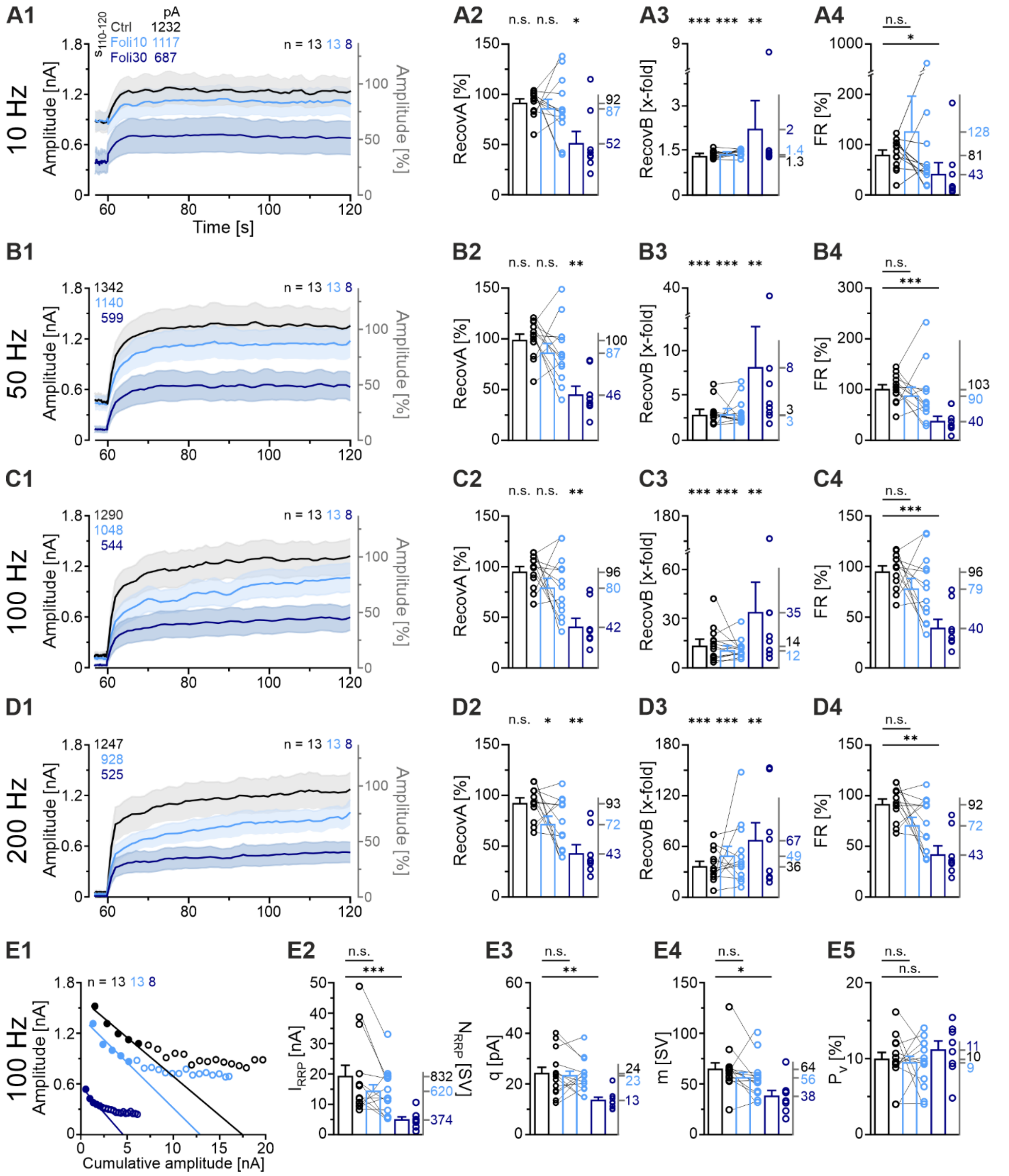

Supplementary Figure S4 - Recovery from depression is impaired, and I_{RRP}, m, and q are decreased upon Foli30 treatment. These results support the findings obtained with Bafi. **(A1)** Time course of eIPSC amplitudes during 1-Hz|60-s recovery after 10-Hz challenge (see Supplementary Figure S3). Upper left numbers show mean amplitudes at s110-120. **(A2-A4)** Statistics for RecovA **(A2)**, RecovB **(A3)**, and FR **(A4)**. Mean percentages shown on right y-axis. **(B-D)** Same as A, but for recovery after 50 Hz **(B1-B4)**, 100 Hz **(C1-C4)**, and 200 Hz challenge **(D1-D4)**. Compared to Ctrl, recovery remains unchanged after Foli10 treatment. In contrast, it decreases ~2-fold after Foli30 treatment. **(E)** Analysis of synaptic parameters. **(E1)** Elmqvist & Quastel plots used to determine I_{RRP} of the three cohorts. **(E2)**

Statistics for I_{RRP}. Mean values of N_{RRP} on the right y-axis. **(E3-E5)** Statistic for q (obtained from a 300 s period over BL and Recov1-Recov4), **(E3)**, m **(E4)**, and P_v **(E5)**. Mean values on the right y-axis. I_{RRP}, N_{RRP}, q, and m remain unchanged after Foli10 treatment, but they are significantly reduced after Foli30 treatment. P_v shows no change in either condition. Variables on the y-axes in B1-D1 are the same as in A1, and variables on the y-axes in B2-D4 are the same as in A2-A4. Time courses are weighted moving averages (SEM lightly shaded). Ctrl and Foli10, n = 13; Foli30, n = 8. See Supplementary Table S10 for details, including statistics.
