# Supplementary material for: Robust inhibitory glycinergic transmission and the effect of bafilomycin, folimycin and EIPA: lessons from the auditory brainstem
Source: Front Cell Neurosci. 2025 Oct 15;19:1625868. doi: 10.3389/fncel.2025.1625868 (PMC12568497; doi:10.3389/fncel.2025.1625868)
Supplement: Supplementary file 4 [file Data_Sheet_3.PDF]

Low frequency sustained stimulation in the absence and presence of bafilomycin (1 Hz | ~40 min)

MNTB-LSO synapses were stimulated at 1 Hz, and recordings lasting > 40 minutes were analyzed from four control neurons and two bafilomycin-treated neurons. Each episode began with a 2-min baseline (BL) period, which was normalized to 100%. The absolute values were BL_{Ctrl} = 714 pA, BL_{Bafi} = 678 pA, yielding a Bafi:Ctrl ratio of 95%.

Following BL, three consecutive 15-min periods were recorded, with brief (few-second) interruptions for quality checks. During these periods, the slice chamber was continuously perfused with ACSF + solvent (Ctrl) or ACSF + solvent + 2 µM bafilomycin (Bafi).

Preliminary results:

Figure 1 shows the time course of eIPSC peak amplitudes. The Ctrl group exhibited stable, robust synaptic transmission, with the mean amplitude of the last 10 pulses at 104% of baseline (746 pA/714 pA). In contrast, the Bafi group showed a progressive decline, reaching 21% of baseline by the end of the recording (165 pA/678 pA). Despite this reduction, synaptic transmission persisted, with a final Bafi:Ctrl ratio of ~20%.

The slope of the Bafi curve during minutes 20-40 was -2.35 %/min, and linear extrapolation predicts an intersection with the y-axis at ~51 min. This corresponds to complete depletion of the vesicle pool after ~3,060 stimulus pulses applied at 1 Hz.

At the end of the recordings, the total integrated current was 1,773 nA for the Ctrl group and 1,015 nA for the Bafi group, yielding a Bafi:Ctrl ratio of ~60%.

Based on previous experiments reported in the main document, we know that bafilomycin requires more than 10 min exert its full effect in brainstem slices. Therefore, we quantified synaptic transfer capacity during minutes 20-40 by integrating the current over that interval. This yielded cumulative values of 976 nA (Ctrl) and 158 nA (Bafi). Assuming a quantal size of 20 pA (see Figure 6C3), these correspond to 48,800 and 7,900 synaptic vesicles, respectively. Similarly, using a quantal size of ~25 pA (see Figure 6C3), the estimates become 39,040 (Ctrl) and 6,320 (Bafi) vesicles. In both cases, the Bafi:Ctrl ratio is 16%.

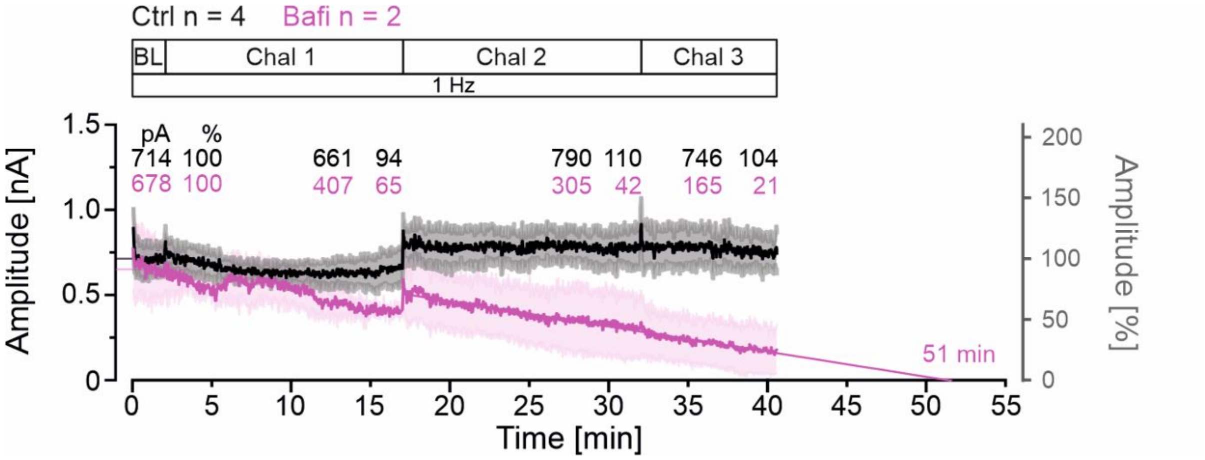

Figure 1
Effect of bafilomycin on eIPSC amplitudes during sustained low-frequency stimulation (1 Hz for ~40 min). Baseline was determined during minutes 1-2, after which drug wash-in began (2 µM Bafi). Two 15-min challenge periods followed: Chal 1 (minutes 3-17) and Chal 2 (minutes 18-32). The third challenge period (Chal 3) was discontinued after 40 min 35 s due to instability in one neuron. A linear extrapolation predicts that the y-axis intersection would occur at ~51 min, suggesting depletion of the SV pool after this time.

Interpretation of the (preliminary) results

We consider three possible explanations for the persistence of synaptic transmission under bafilomycin treatment:

1. Incomplete inhibition of V-ATPase by Bafi.

   Although we argue in the manuscript that incomplete inhibition of V-ATPase is unlikely, it remains a formal possibility. If true, this would represent a significant limitation of Bafi as a pharmacological tool. Moreover, the validity of numerous studies that relied on Bafi to block the vesicular H^+ pump would need to be reconsidered. Preliminary experiments indicate that a Bafi concentration of 2 µM is sufficient to abolish transmission when MNTB-LSO synapses are stimulated a 1 Hz for nearly one hour (Supplementary Figure S5). However, the number of recordings in these experiments must be increased before any definitive conclusions can be drawn.

2. A sufficiently large vesicle pool at MNTB-LSO synapses.

   It cannot be excluded that MNTB-LSO synapses contain a vesicle pool large enough to sustain transmission for the full 40-min recording, even in the absence of ongoing SV refilling via V-ATPase. However, we find this unlikely. In Figure 1, the extrapolated Bafi curve intersects the y-axis at ~51 min, corresponding to ~3,060 stimulus pulses. For comparison, a 50-Hz challenge involved a similar number of pulses (3,000) delivered

within just 1 min, yet the steady-state transmission remained at 88 pA (7%; cf. Figures 3C1 and 3C5), demonstrating that transmission was maintained. Moreover, as indicated in Figure 3A1-A3, the full protocol for transmission analysis in the presence of Bafi consisted of two 8-min blocks (Bafi10 and Bafi30), each containing 10-Hz, 50-Hz, 100-Hz, and 200-Hz challenge periods interleaved with four recovery periods. In total, this protocol encompassed 43,200 stimulus pulses during the challenge periods plus 480 stimulus pulses during the recovery. Considering challenge periods only, the cumulative current amplitude amounted to 3,183 nA (2,256 nA for Bafi10, 927 nA for Bafi30; see Figure 4E2). Assuming a quantal size of 25 pA, this corresponds to 127,320 released vesicles (159.150 SVs if q = 20 pA). We reason that these numbers are too high to be explained solely by a large vesicle pool. Rather, ongoing re(filling) of vesicles, despite V-ATPase blockade, provides the more likely explanation.

3. The presence of an alternative H^+ transporter, such as NHE.

   A third possibility is that synaptic vesicle acidification—and thus neurotransmitter loading—is maintained by an alternative proton source, such as a Na^+/H^+ exchanger (NHE). This mechanism could account for the sustained release observed in our challenge experiments. Future experiments using NHE(6)-specific inhibitors will be essential to test this hypothesis.
